# Identification and validation of γ-Linolenic acid as a natural FABP5 inhibitor in hepatocellular carcinoma through deep learning and experimental approaches

**DOI:** 10.3389/fimmu.2026.1700347

**Published:** 2026-01-28

**Authors:** Yuan An, Hongyu Liu, Wei Li

**Affiliations:** Department of Hepatobiliary-Pancreatic Surgery, China-Japan Union Hospital of Jilin University, Key Laboratory of Jilin Provincial for Precise Diagnosis and Therapy of Hepatobiliary-Pancreatic Tumors, Changchun, China

**Keywords:** FABP5, hepatocellular carcinoma, machine learning, molecular dynamics simulation, γ-linolenic acid

## Abstract

**Background:**

Fatty acid binding protein 5 (FABP5) is implicated in hepatocellular carcinoma (HCC) progression and represents a potential therapeutic target.

**Methods:**

We integrated machine learning–based virtual screening, molecular docking and molecular dynamics simulations to identify natural compounds with high binding affinity to FABP5. Candidate compounds were further validated by in-vitro assays in HCC cell lines, including proliferation, migration/invasion, apoptosis/ferroptosis-related readouts, and mechanistic validation.

**Results:**

The optimized models enabled efficient screening of natural products and prioritized γ-linolenic acid (GLA) as a top candidate FABP5 inhibitor. Docking and simulations supported stable binding and key residue interactions. Experimentally, GLA inhibited HCC cell proliferation and aggressiveness and promoted cell death–related pathways consistent with anti-tumor activity.

**Conclusion:**

Our deep learning–guided workflow identified γ-linolenic acid as a natural FABP5 inhibitor and supports its potential as a lead compound for HCC therapy.

## 1 Introduction

Hepatocellular carcinoma (HCC) represents an aggressive form of liver cancer that significantly contributes to global mortality, underscoring an urgent necessity for novel therapeutic interventions (1–3).A hallmark of HCC progression is metabolic reprogramming, particularly in lipid metabolism, which enables tumor cells to sustain rapid proliferation, resist stress, and acquire invasive and metastatic capabilities (4). Increasing evidence indicates that dysregulated fatty acid uptake, transport, and utilization are not only essential for tumor growth but also critically contribute to tumor dissemination and metastasis, positioning lipid metabolic pathways as attractive therapeutic targets in HCC.

Fatty Acid Binding Proteins (FABPs) constitute a family of intracellular lipid-binding proteins essential for transporting hydrophobic molecules, including fatty acids, throughout cellular compartments (5–7). Among them, FABP5 has emerged as a key mediator linking lipid metabolism to cancer aggressiveness. FABP5 expression is markedly elevated in HCC tissues and is strongly associated with poor prognosis, increased invasiveness, and metastatic potential (8). Beyond serving as a passive lipid carrier, FABP5 actively participates in oncogenic signaling by facilitating lipid-mediated activation of downstream pathways. Canonically, FABP5 enhances fatty acid delivery to nuclear receptors such as PPARβ/δ, thereby promoting transcriptional programs that support cell survival, proliferation, and migration. In addition, emerging evidence suggests that FABP5 also functions through non-canonical mechanisms, including modulation of oxidative stress responses, cytoskeletal dynamics, and membrane-associated signaling pathways, further amplifying malignant phenotypes. Genomic analyses reveal frequent co-amplification of FABP5 with other FABP family members across multiple cancer types, contributing to its aberrant overexpression in malignant cells. Functionally, inhibition or silencing of FABP5 significantly suppresses HCC cell viability, proliferation, and motility (9, 10). Collectively, these findings establish FABP5 as a central regulator of lipid-driven oncogenic and metastatic processes in HCC, thereby justifying its selection as a therapeutic target in this study.

γ-Linolenic Acid (GLA), a polyunsaturated fatty acid, has concurrently attracted considerable attention due to its documented anticancer and antibiotic properties (11, 12). GLA reportedly exhibits selective anticancer activity with minimal systemic toxicity, partly through modulating steroid hormone receptors. Preclinical studies revealed that GLA treatment inhibited the growth of Huh7 cells, a human HCC cell line, by inducing reactive oxygen species (ROS) generation, lipid peroxidation, and mitochondrial dysfunction leading to apoptosis (13). Moreover, GLA has been shown to enhance the efficacy of conventional anticancer agents and to induce apoptotic cell death across a variety of cancer models, suggesting that it may act through multiple, interconnected mechanisms (14, 15). This broad activity spectrum positioned GLA as a promising candidate for a multifaceted therapeutic approach, potentially involving FABP5 inhibition.

Considering the pivotal role of FABP5 in lipid metabolic reprogramming, tumor progression, and metastasis in HCC, together with the established anticancer activity of GLA, there is a compelling rationale to explore GLA as a natural modulator of FABP5. Identifying bioactive compounds from dietary and medicinal sources offers promising opportunities for developing safer and more accessible therapeutic strategies. In this study, we integrate computational prediction with experimental validation to investigate the potential of GLA as an FABP5-targeting agent in HCC. Specifically, we employ machine learning–based models and virtual screening of a drug–food homology library to identify candidate FABP5 inhibitors, followed by experimental evaluation of GLA in Huh7 cells. The effects of GLA on cell viability, migration, cytoskeletal organization, apoptosis, cellular morphology, and biomechanical properties are systematically examined, alongside assessment of FABP5 protein expression. We hypothesize that GLA-mediated modulation of FABP5 disrupts lipid-driven oncogenic signaling, thereby impairing HCC cell proliferation, migration, and survival, and inducing phenotypic alterations characteristic of a less aggressive malignant state.

## 2 Materials and methods

### 2.1 Computational methods

#### 2.1.1 Data collection and standardization

Data collection and standardization were performed using CHEMBL, PUBCHEM, and literature searches, yielding information on FABP5 inhibitor molecules (16, 17). A total of 682 inhibitors and non-inhibitor records were initially included. Given the presence of 490 inhibitor molecules, we randomly extracted additional molecules from PUBCHEM to balance the dataset, resulting in a negative dataset of 490 molecules. Subsequently, we standardized the SMILES format by removing charges, small fragments, tautomers, ions, metals, and hydrogen atoms (18). The dataset was further divided into a training set and a testing set at an 8:2 ratio, with all data stored in the file data.csv.

#### 2.1.2 Dataset evaluation and clustering

The SMILES strings for each molecule were parsed using the RDKit cheminformatics library (19) and converted into molecular objects. We computed molecular descriptors including molecular weight (MolWt) (20), topological polar surface area (TPSA) (21), number of hydrogen bond donors (NumHDonors) (22), number of hydrogen bond acceptors (NumHAcceptors), LogP (MolLogP) (23), and molecular volume (MolMR) (24) using RDKit’s Descriptors and MoleculeDescriptors modules. Histograms showing the distribution of these properties were generated using the Seaborn histplot function, categorized by inhibitory activity.

To analyze the structural similarity among toxic small molecules, clustering analysis was conducted. Initially, SMILES strings were extracted from the raw data and converted into molecular objects using the rcdk package. Each molecule was encoded using an extended fingerprint type, resulting in binary vector representations (2048 bits) reflecting specific structural features. For visualization of the high-dimensional fingerprint data, we applied the t-SNE algorithm to reduce the 2048-dimensional fingerprint vectors to three dimensions. The parameters for t-SNE were set as follows: dimensions (dims) = 3, perplexity = 40, and θ (theta) = 0.0. Hierarchical clustering using Ward.D2 was subsequently performed on the t-SNE dimensionality-reduced data. By calculating the Euclidean distances among samples and constructing a hierarchical clustering tree, molecules were grouped into 3 clusters, each assigned distinct labels. Within each cluster, a Tanimoto similarity matrix was computed to select representative molecules. The representative molecule was determined by the highest average similarity within its cluster and was uniquely identified in each cluster. Finally, the 3D visualization of the t-SNE data was presented using the plotly package, with colors distinguishing different clusters, and representative molecules labeled as “REP” for clarity.

#### 2.1.3 Model construction and evaluation

In this study, molecular fingerprints employed included Morgan fingerprints (1024 bits, similar to extended connectivity fingerprints) (25), RDKit fingerprints (path-based fingerprints capturing substructures), MACCS keys (26) (166 bits, predefined structure keys commonly used for rapid screening), AtomPairs fingerprints (27) (1024 bits, encoding relationships between atom pairs and their topological distances), and topological pharmacophore-based fingerprints (28) (TPBFP, 1024 bits, encoding atom triplet interactions based on pharmacophore features).

To enhance predictive performance, four machine learning models were selected with hyperparameter optimization conducted for each. The Random Forest (RF) model (29), an ensemble recursive partitioning method, optimized hyperparameters including number of trees (n_estimators: 50, 100, 200), splitting criteria (criterion: “gini” and “entropy”), maximum tree depth (max_depth: None, 5, 10, 15), minimum samples per leaf (min_samples_leaf: 1-10), and number of features considered per split (max_features: “log2”, “auto”, “sqrt”). The RF model improves accuracy and robustness by constructing multiple decision trees based on random subsets of training data. The Support Vector Machine (SVM) model (30) identifies the optimal hyperplane by maximizing class boundaries in feature space, optimizing kernel function coefficients (gamma: ‘scale’, ‘auto’) and error penalty parameters (C: 0.01, 0.1, 1, 10). The Multi-Layer Perceptron (MLP) (31) neural network optimized hidden layer sizes (hidden_layer_sizes: (50), (100), (50, 50)), activation functions (activation: ‘tanh’, ‘relu’), solvers (solver: ‘sgd’, ‘adam’), initial learning rates (learning_rate_init: 0.001, 0.01), and maximum iterations (max_iter: 10000). Finally, the Extreme Gradient Boosting (XGBoost) model (32), an advanced gradient boosting framework, optimized hyperparameters such as maximum tree depth (max_depth: 3, 5, 7, 10, 15).

The dataset was initially divided into training and testing sets using an 8:2 ratio. To further ensure model robustness and reduce the risk of overfitting, a five-fold cross-validation strategy was subsequently applied to the training dataset. In this procedure, the data were randomly partitioned into five subsets, with four folds used for model training and one fold reserved for validation in each iteration, and performance metrics were averaged across all folds. Model performance was comprehensively evaluated using multiple metrics, including the area under the receiver operating characteristic curve (AUC), sensitivity (SE), specificity (SP), Matthews correlation coefficient (MCC), accuracy (ACC), precision (P), F1 score (F1), and balanced accuracy (BA). These metrics provide a robust and detailed assessment of predictive performance, particularly for binary classification tasks involving limited or potentially imbalanced datasets.

#### 2.1.4 Virtual screening and molecular docking of bioactive compounds

From the Traditional Chinese Medicine Systems Pharmacology (TCMSP) database (33), 3178 standardized molecules from 91 medicinal and edible sources were obtained. These molecules were initially predicted for FABP5 inhibitory activity using the optimal Morgan-XGBoost model.

For molecular docking, the FABP5 structure was obtained from the Protein Data Bank (PDB ID: 1B56). The protein was prepared by retaining chain A, removing other chains and ligands, removing charges, and adjusting protonation states to pH 7.4. The hydrogen-bond network was optimized, followed by energy minimization using the amber14/protein.ff14SB force field. Semi-flexible docking was performed using the AI-based molecular docking method, CarsiDock. A complementary docking approach was also employed using AutoDock Vina (34). For this, the FABP5 protein structure was sourced from PDB (1b56). Small molecule structures, Ganolucidic Acid E and Linolenic acid, were obtained from PubChem as 3D.sdf files. Tetracosanoic acid was obtained as a 2D.sdf from PubChem and converted to 3D via ChemDraw, with all small molecules subsequently exported to.pdb format via PyMOL. The docking box settings for AutoDock Vina were specified as: center_x = 49.137, center_y = 21.547, center_z = 32.93, size_x = 44.25, size_y = 47.25, size_z = 43.5.

### 2.2 Experimental methods

#### 2.2.1 Cell culture and reagents

Huh7 human HCC cells were utilized for all experimental procedures. These cells are adherent and typically grow as 2D monolayers. All cell culture operations were conducted under strict sterile conditions, with experimental benches and equipment disinfected with 75% ethanol and sterilized by UV irradiation for 30 minutes prior to use. The culture medium consisted of DMEM high glucose medium supplemented with 1% penicillin-streptomycin (double antibiotic) and 10% fetal bovine serum (FBS). For cell resuscitation, Huh7 cell aliquots were rapidly thawed from liquid nitrogen in a 37 °C water bath. The thawed cells were then transferred to a centrifuge tube, 3 mL of prepared culture medium was added, followed by centrifugation at 1300 rpm for 5 minutes, and the supernatant was discarded. Cells were resuspended and uniformly seeded into 60 mm cell culture dishes, then incubated in a 37 °C incubator with 5% CO2. Routine cell passaging was performed when Huh7 cells reached 80-90% confluency under microscopic observation, ensuring stable cell growth for subsequent experimental use (35).

#### 2.2.2 Cell viability assessment

Huh7 cells were seeded at a density of 7 x 10^4 cells/mL, with 100 µL per well, into 96-well plates and incubated until adherence. After cell attachment, the culture medium was replaced with DMEM containing GLA at various concentrations (0.15, 0.2, 0.25, 0.3, 0.35 mM) for 24 hours (36). A control group, consisting of Huh7 cells cultured in normal medium for 24 hours, was included. Each concentration and the control group were set up with five replicate wells. Following the 24-hour treatment period, MTT solution was added to each well. Cell viability was then assessed by detecting the optical density (OD) values at a wavelength of 490 nm.

#### 2.2.3 Cell migration assay

Huh7 cells were uniformly seeded into 6-well plates to achieve 80-90% confluence and further cultured until a stable 80% confluent monolayer was formed. A linear “wound” or scratch was then created on the confluent monolayer using a 20 µL pipette tip. This method is commonly referred to as a scratch assay. Two groups were established: a control group (Huh7 cells cultured in normal medium) and an experimental group (Huh7 cells cultured with 0.25 mmol/L GLA). Images of the wound were captured and wound distances were measured at 0 hours (immediately after scratching and treatment addition), 12 hours, and 24 hours. The migration distances of Huh7 cells were then compared between the experimental and control groups over time to assess the effect of GLA on cell migration.

#### 2.2.4 Cytoskeleton staining and imaging

Glass coverslips were prepared by soaking in 75% ethanol overnight, followed by 30 minutes of sonication, and then dried under UV sterilization in a sterile hood. Huh7 cells were seeded onto the prepared coverslips and allowed to grow for 12 hours. Control and experimental groups were established, with the experimental group treated with 0.25 mmol/L GLA for 24 hours. After treatment, the culture medium was removed, and cells were washed three times with PBS for 5 minutes each. Cells were then fixed with 4% glutaraldehyde for 30 minutes. The cell staining procedure was performed in the dark. Cells underwent acetone dehydration for 5 minutes, followed by three PBS washes for 10 minutes each. A 200 µL volume of diluted phalloidin-FITC solution was applied to cover the samples and incubated in the dark for 30 minutes. After subsequent PBS washes, DAPI stain was applied for 30 seconds, followed by another PBS wash. For fluorescence microscopy, samples were placed on the microscope stage under dark conditions. Nuclei were imaged using blue excitation light at 20 nms. After confirming cell position, the excitation light was switched to green, and exposure was adjusted to 600 ms to capture cytoskeleton images. Images were saved once clear exposure was achieved. The detailed observation of cytoskeletal changes, such as irregular arrangement, structural protrusions, and cellular contraction, in response to GLA is not merely a descriptive morphological finding but directly links to the observed reduction in cell migration and viability. The cytoskeleton is fundamental for cell shape, adhesion, and movement; thus, its disruption provides a mechanistic explanation for the functional impairments observed in other assays.

#### 2.2.5 Cell death analysis (flow cytometry)

Huh7 cells were uniformly seeded into 35 mm culture dishes and allowed to grow until they reached 80-90% confluence. Cells were then treated with GLA at various concentrations (0, 0.1, 0.15, 0.2, 0.25 mM) in culture medium for 24 hours. Following treatment, Huh7 cells were resuspended and washed twice with PBS. Cells were then incubated with a flow cytometry kit, typically involving Annexin V and Propidium Iodide (PI). Annexin V detects early apoptotic cells by binding to phosphatidylserine exposed on the outer membrane, while PI stains late apoptotic or necrotic cells with compromised membrane integrity. Samples were subsequently analyzed using a flow cytometer to quantify cell death.

#### 2.2.6 SEM characterization of Huh7 cell morphology

The initial preparation steps, including cell fixation with glutaraldehyde, were performed as described in the cytoskeleton imaging protocol. Following fixation, cells underwent sequential dehydration with graded ethanol solutions: 50%, 70%, 80%, 85%, 90%, and 95% ethanol, each for 10 minutes, followed by three 5-minute immersions in absolute ethanol. Samples were then covered with tert-butanol and frozen overnight at -20°C. Subsequently, samples were vacuum-dried at room temperature for 20 minutes to remove tert-butanol. A 5nm gold coating was then applied to the sample surface using a vacuum sputter coater. For SEM scanning, samples were placed in the SEM vacuum chamber and secured with conductive adhesive. The SEM parameters were set as follows: High Voltage 5.00 kV, Spot 3.5, and Chamber Pressure 30 Pa. Scanning commenced after beam activation, with adjustments to contrast, and sequential scanning from low to high magnification until clear images were obtained. SEM provides high-resolution insights into the surface morphology of cells, complementing the internal cytoskeletal observations. The detailed visualization of nuclear protrusion, significant damage to the cell matrix and pseudopods, and partial detachment from the substrate at increasing concentrations of GLA indicates severe and widespread cellular damage, affecting external structures and adhesion. This comprehensive damage profile further underscores the potent cytotoxic effects of GLA beyond just internal structural changes.

#### 2.2.7 AFM scanning of Huh7 cells

Huh7 cells were seeded into 35 mm culture dishes and allowed to adhere. For treatment, Huh7 cells in 96-well plates were exposed to GLA at concentrations of 0.15 and 0.2 mmol/L for 24 hours, with an untreated control group. Prior to AFM scanning, the culture medium was replaced with serum-free DMEM. An MLCT probe was selected for AFM measurements, based on the specific characteristics of the cells. Cell images were acquired using the CCD camera of an optical microscope after fixing the cells on the sample stage. Force calibration was performed in liquid mode, involving adjustments to the laser, four-quadrant detector, and probe elastic coefficient. Scanning was conducted in QI mode with a resolution of 128x128 pixels.

#### 2.2.8 Western blot analysis of FABP5 expression

Treated Huh7 cell pellets were collected, washed with PBS buffer, and then lysed to extract total protein. Protein concentration was measured to ensure consistent loading amounts for each sample, followed by mixing with an appropriate volume of 5x Loading Buffer. Samples were denatured by heating in a metal bath at 100 °C for 10 minutes prior to Western blot electrophoresis. Proteins were then transferred to a PVDF membrane. The membrane was incubated with primary antibodies targeting FABP5 and β-actin (used as a loading control), followed by incubation with secondary antibodies at room temperature for 2 hours. Chemiluminescent substrate was then added, and protein bands were visualized using a multi-functional imager. The observed dose-dependent downregulation of FABP5 protein expression by γ-Linolenic Acid provides a direct and crucial mechanistic link between the compound and its proposed target. This experimental validation strongly supports the initial computational prediction, integrating the entire study’s narrative and suggesting that FABP5 modulation is a key mechanism through which GLA exerts its multifaceted anticancer effects in Huh7 cells.

#### 2.2.9 Statistical analysis and reproducibility

All computational analyses were conducted using Python (version 3.11.7). Data preprocessing and numerical operations were performed with NumPy (version 1.26.4) and Pandas (version 2.1.4). Molecular descriptor calculation and fingerprint generation were carried out using RDKit (version 2025.3.2). Machine learning model construction, training, and evaluation were implemented using scikit-learn (version 1.7.2) and XGBoost (version 3.1.2). All software packages were used with default parameters unless otherwise specified to ensure reproducibility.

Cell line were provided by Jilin University, and all cells were routinely tested and confirmed to be free of mycoplasma contamination prior to experimentation.

All biological experiments were performed with at least three independent biological replicates (n = 5). Quantitative data are presented as mean ± standard deviation (SD). Statistical analyses were conducted using Student’s t-test for comparisons between two groups and one-way analysis of variance (ANOVA) for comparisons among multiple groups. A p value of < 0.05 was considered statistically significant.

## 3 Results

### 3.1 Evaluation of molecular properties

**Figure 1:**
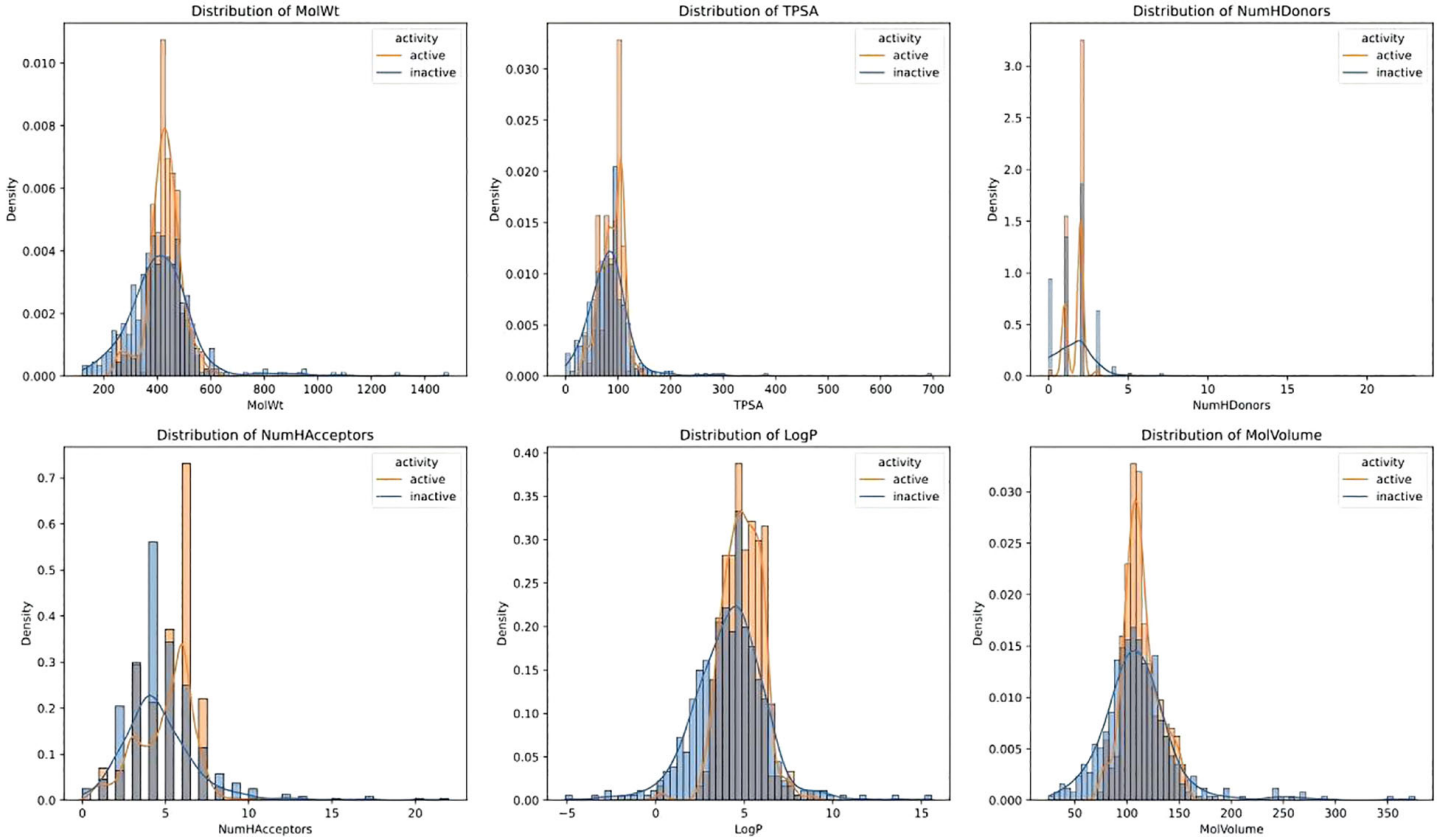
Distribution of active and inactive compounds across six molecular descriptors. Density distributions of six molecular descriptors, including molecular weight (MolWt), topological polar surface area (TPSA), number of hydrogen bond donors (NumHDonors), number of hydrogen bond acceptors (NumHAcceptors), LogP, and molecular volume (MolVolume), comparing active and inactive compounds in the dataset.

**Figure 1** illustrated the distribution differences between active and inactive compounds across six molecular descriptors: molecular weight (MolWt), topological polar surface area (TPSA), number of hydrogen bond donors (NumHDonors), number of hydrogen bond acceptors (NumHAcceptors), LogP values, and molecular volume (MolVolume). Active compounds predominantly exhibit molecular weights ranging from 300 to 500, with a peak around 400, whereas inactive compounds have a broader distribution ranging from 200 to 600. For TPSA, active compounds are mainly concentrated between 50 and 150, with a peak around 100, while inactive compounds span a wider range from 100 to 200. Regarding NumHDonors, active compounds cluster between 0 to 3, peaking between 0 and 1, while inactive compounds have a wider range, particularly dense between 0 and 5. Both active and inactive compounds peak in the range of 4 to 6 for NumHAcceptors, but inactive compounds display a more dispersed distribution. LogP distribution shows active compounds concentrated between 3 and 5, with higher density in regions of higher LogP values (>5), whereas inactive compounds have greater density in lower LogP regions (<3). In terms of molecular volume (MolVolume), active compounds cluster between 50 to 150, peaking around 100; inactive compounds have similar distributions but gradually decrease at volumes greater than 150. These findings suggest that molecular weight, TPSA, number of hydrogen bond donors, and LogP values may significantly predict active versus inactive compounds.

**Figure 2:**
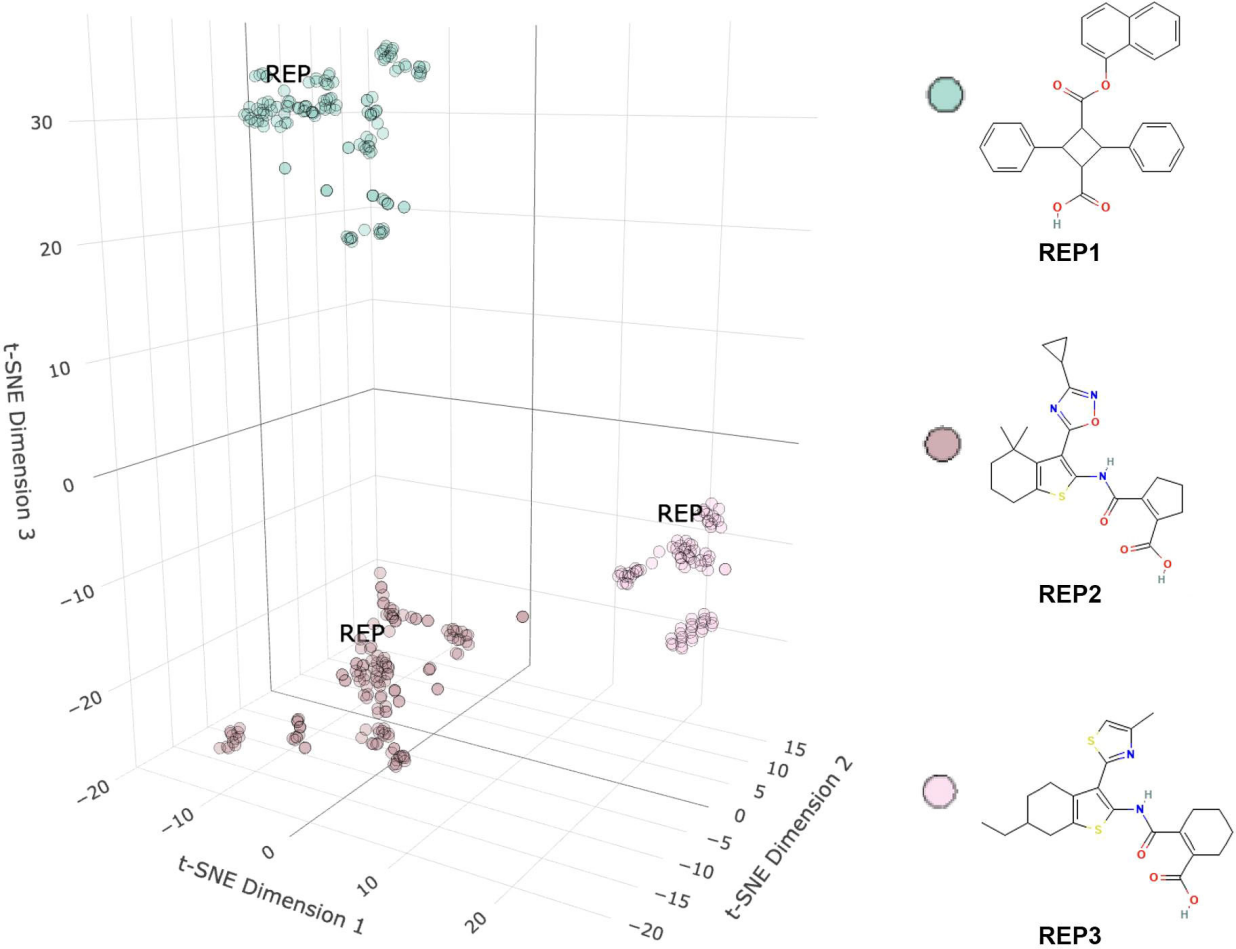
Clustering results of inhibitor molecules. t-SNE visualization of inhibitor molecules based on molecular descriptors. Each point represents one compound, and three major clusters (REP1–REP3) are identified. Representative molecular structures from each cluster are shown.

Cluster results are presented in **Figure 2**, and corresponding data can be found in the clustered_data table. Clustering resulted in three distinct groups: the first group represents molecules of 3-naphthalen-1-yloxycarbonyl-2,4-diphenylcyclobutane-1-carboxylic acid; the second group represents 2-[3-(3-cyclopropyl-[1,2,4]oxadiazol-5-yl)-4,4-dimethyl-4,5,6,7-tetrahydro-benzo[b]thiophen-2-ylcarbamoyl]-cyclopent-1-enecarboxylic acid; the third group represents 2-[6-ethyl-3-(4-methyl-thiazol-2-yl)-4,5,6,7-tetrahydro-benzo[b]thiophen-2-ylcarbamoyl]-cyclohex-1-enecarboxylic acid. Notably, the second and third groups share relatively similar molecular structures.

### 3.2 Model construction

**Figure 3:**
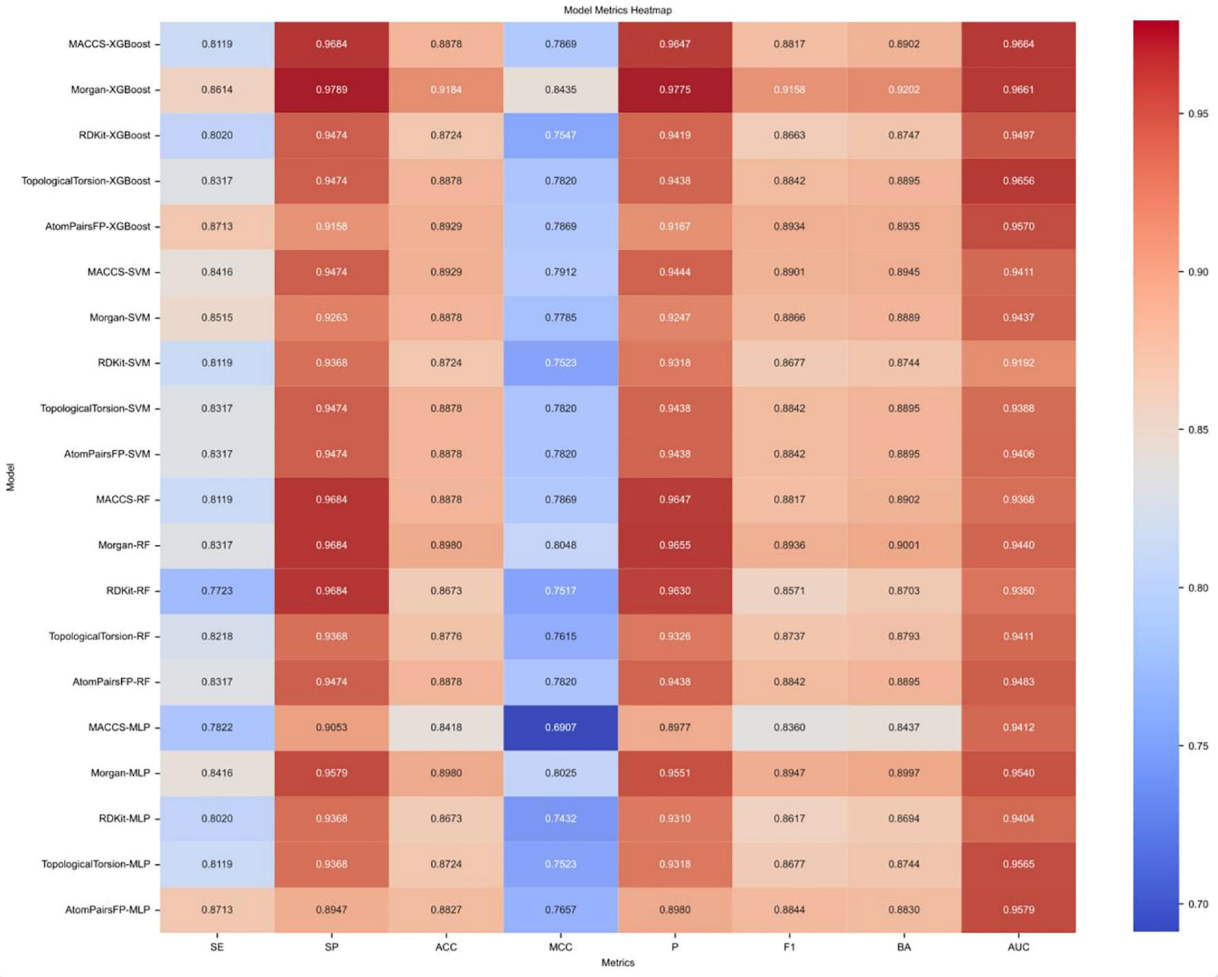
Heatmap of performance metrics for various machine learning models. Heatmap summarizing the performance of different machine learning models using various molecular fingerprints. Metrics include sensitivity (SE), specificity (SP), accuracy (ACC), Matthews correlation coefficient (MCC), precision (P), F1-score (F1), balanced accuracy (BA), and area under the ROC curve (AUC).

Performance metrics for various models were depicted in **Figure 3**. Among all algorithms evaluated, the XGBoost model demonstrated the most balanced and superior performance, achieving high levels of specificity (SP), accuracy (ACC), and area under the curve (AUC) across most molecular fingerprint combinations, with most AUC values exceeding 0.94. For example, Morgan-XGBoost achieved an AUC of 0.9661, while AtomPairsFP-XGBoost reached an AUC of 0.9570. XGBoost’s overall stability and effectiveness in handling imbalanced datasets make it the optimal choice for this task. Support Vector Machine (SVM) also performed well with certain fingerprints such as Morgan (AUC 0.9437) and RDKit (AUC 0.9192), showing high specificity and strong classification capability. Despite these strengths in specific combinations, the overall performance of SVM was slightly inferior to XGBoost, particularly regarding sensitivity (SE). Random Forest (RF) exhibited balanced performance across most fingerprint combinations, with Morgan-RF achieving an AUC of 0.9440 and specificity of 0.9684. Although RF showed strong performance in terms of specificity and AUC, it was slightly weaker in sensitivity (SE) and MCC compared to XGBoost, thus suitable for scenarios requiring stable but less complex modeling. Multi-Layer Perceptron (MLP) algorithms exhibited less stability, particularly in MCC and F1-score metrics (RDKit-MLP MCC of 0.7432, MACCS-MLP MCC of 0.6907). Despite good AUC values with certain fingerprints (AtomPairsFP-MLP AUC 0.9579), the overall performance lacked balance.

Analysis of molecular fingerprint performance indicated the Morgan fingerprint consistently performed well across multiple algorithms, particularly with XGBoost (AUC 0.9661) and RF (AUC 0.9440), reflecting its versatility and stability in cheminformatics applications. AtomPairsFP fingerprint performed notably well with XGBoost (AUC 0.9570) and MLP (AUC 0.9579), indicating good adaptability to these algorithms. Overall, AtomPairsFP demonstrated balanced performance across various algorithms. RDKit fingerprints showed average performance, with notable results in RF (AUC 0.9350) and SVM (AUC 0.9192), revealing limitations with certain algorithms. MACCS fingerprints performed moderately, achieving AUC scores between 0.93 and 0.94 across multiple models, albeit with lower sensitivity (SE) and MCC scores. TopologicalTorsion fingerprints performed well with XGBoost and RF (AUC 0.9656 and 0.9411 respectively), though slightly less effective with other algorithm combinations.

**Figure 4:**
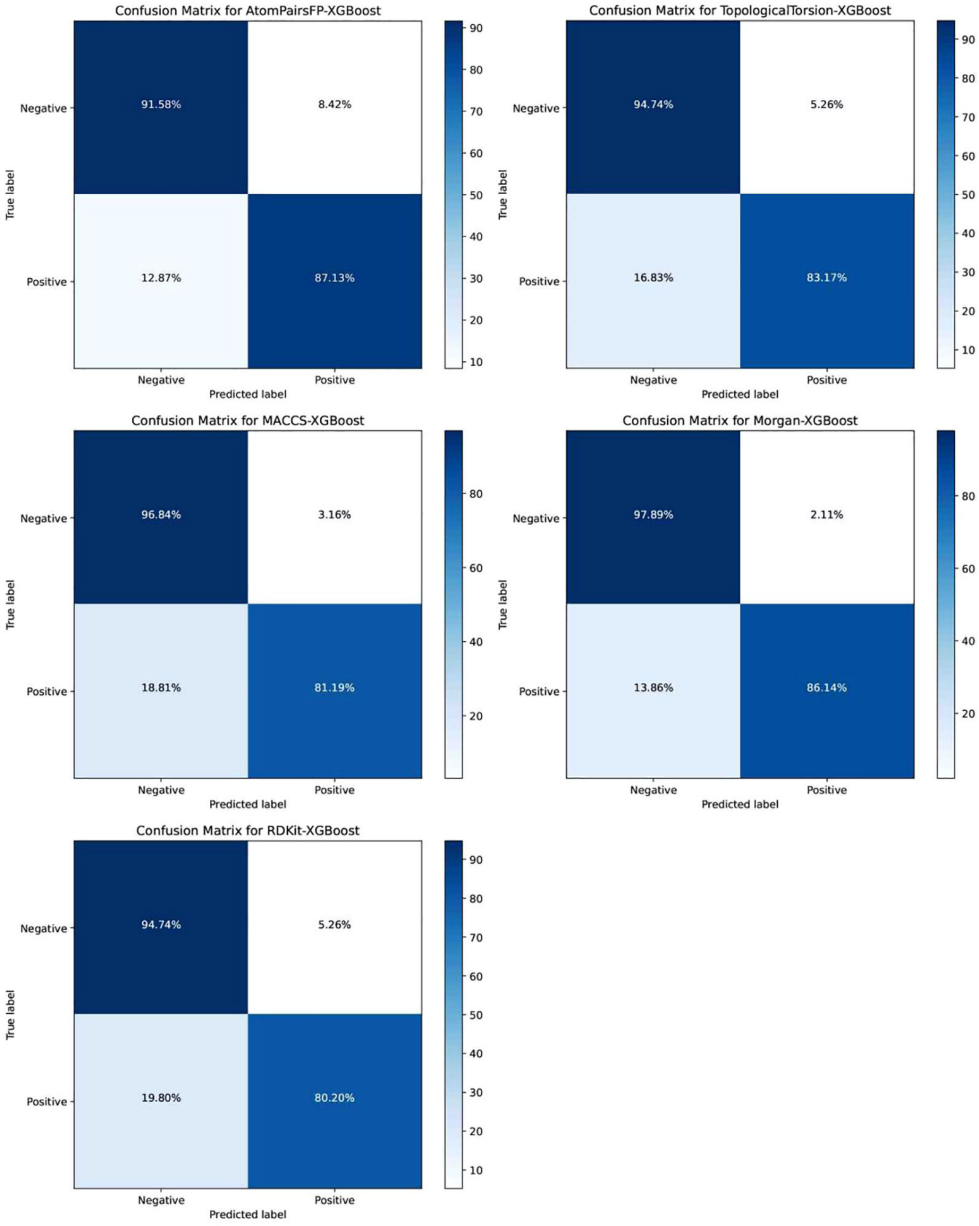
Confusion matrices of XGB models with different fingerprint inputs. Considering all metrics, Morgan-XGBoost is identified as the optimal model combination, achieving top-tier results across specificity (SP), accuracy (ACC), balanced accuracy (BA), F1-score, and AUC (0.9661), thus demonstrating outstanding classification performance and stability.

Confusion matrices for XGB models using different fingerprints as input are shown in **Figure 4**. These matrices illustrate classification performance across AtomPairsFP, TopologicalTorsion, MACCS, Morgan, and RDKit fingerprints in combination with the XGBoost model. The Morgan-XGBoost combination emerged as the best, demonstrating the highest True Negative Rate (TNR) at 97.89% and a high True Positive Rate (TPR) at 86.14%, indicating balanced and robust classification performance. MACCS-XGBoost exhibited very high TNR (96.84%) but lower TPR (81.19%), excelling in negative class identification but showing limitations in positive class performance. AtomPairsFP-XGBoost had the best positive class recognition (TPR 87.13%) but slightly weaker negative class identification (TNR 91.58%). RDKit-XGBoost had weaker positive class recognition (TPR 80.20%) but strong negative class performance (TNR 94.74%).

### 3.3 Prediction of drug-food homology molecules

A total of 70 molecules predicted as positive FABP5 inhibitors were screened from a library of drug-food homology compounds. Molecular docking results identified three potential natural FABP5 inhibitors: Ganolucidic Acid E, Tetracosanoic acid, and Linolenic acid. The docking binding energies (AutoDock Vina) for these molecules were -6.0 kcal/mol for Ganolucidic Acid E, -5.8 kcal/mol for Tetracosanoic acid, and -6.5 kcal/mol for Linolenic acid. Analysis of protein-ligand interactions revealed key hydrogen bond formations: Ganolucidic Acid E formed hydrogen bonds with SER66 and THR68 of FABP5; Tetracosanoic acid formed hydrogen bonds.

with THR56 and ARG109 of FABP5; and Linolenic acid formed a hydrogen bond with ASP79 of FABP5. The identification of specific amino acid residues involved in hydrogen bonding with the predicted inhibitors provides a crucial structural basis for their inhibitory activity. Furthermore, the quantitative binding energies and specific interactions help prioritize lead compounds, with Linolenic acid showing the most favorable binding energy, its selection for subsequent experimental validation.

**Table 1** presents the binding free energies calculated using the MM-PBSA method after 100ns molecular dynamics simulation for Ganolucidic Acid E, Tetracosanoic acid, and Linolenic acid. Among these, Linolenic acid exhibited the strongest binding affinity to FABP5, with a total binding free energy (ΔG total) of −31.50 ± 1.80 kcal/mol. This strong affinity was primarily driven by substantial van der Waals (ΔE vdW=−28.50 ± 2.30 kcal/mol) and electrostatic interactions (ΔE ele=−18.50 ± 3.20 kcal/mol). This favorable binding profile suggests Linolenic acid as the most promising candidate for further investigation.

**Table 1:** Results of MM-PBSA.

System	Ganolucidic Acid E	Tetracosanoic acid	Linolenic acid
ΔEvdW (kcal/mol)	−32.50 ± 2.11	−13.60 ± 3.10	−28.50 ± 2.30
ΔEele (kcal/mol)	−17.80 ± 1.75	−3.05 ± 2.10	−18.50 ± 3.20
ΔGgas (kcal/mol)	−50.30 ± 2.84	−16.65 ± 3.70	−47.00 ± 4.20
ΔGsolv (kcal/mol)	32.50 ± 2.40	10.10 ± 2.95	15.50 ± 3.60
ΔGtotal (kcal/mol)	−17.80 ± 1.22	−6.55 ± 1.55	−31.50 ± 1.80

### 3.4 GLA inhibits Huh7 cell viability and migration

**Figure 5:**
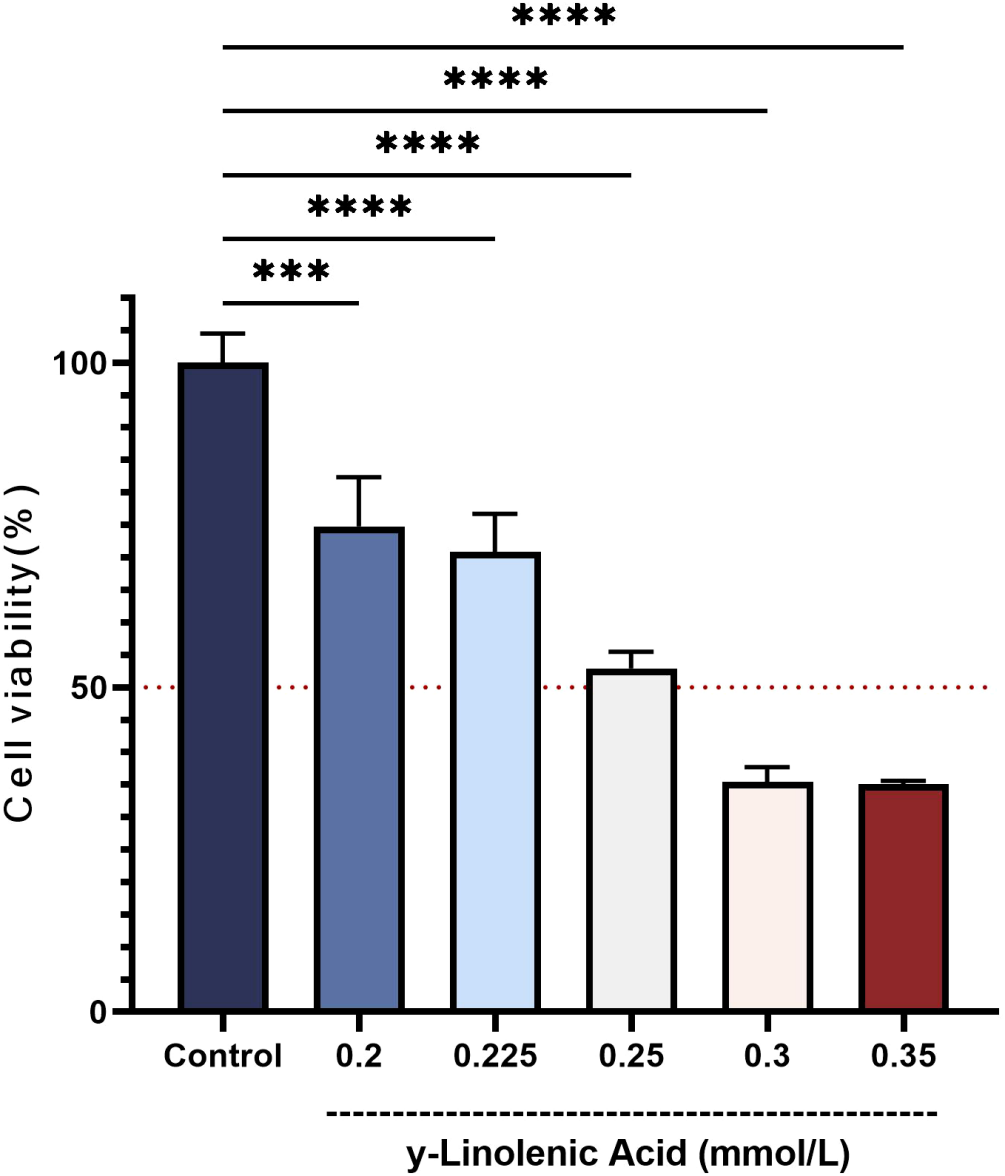
γ-Linolenic Acid Inhibits Huh7 Cell Viability. Huh7 cells were treated with various concentrations of γ-Linolenic Acid for 24 hours, and cell viability was measured by MTT assay. Data are presented as mean ± SD (n = 5). Statistical significance was determined compared with the control group (**** p < 0.0001).

The effect of GLA on Huh7 cell viability was assessed using the MTT assay (**Figure 5**). Huh7 cells treated with GLA at concentrations ranging from 0.15 to 0.35 mmol/L for 24 hours exhibited a dose-dependent reduction in cell viability compared to the control group (cells cultured in normal medium for 24 hours). As the concentration of GLA increased, the cell viability progressively decreased. A concentration of 0.25 mmol/L was selected for subsequent experiments, as it was observed to be close to the IC_50_ value.

**Figure 6:**
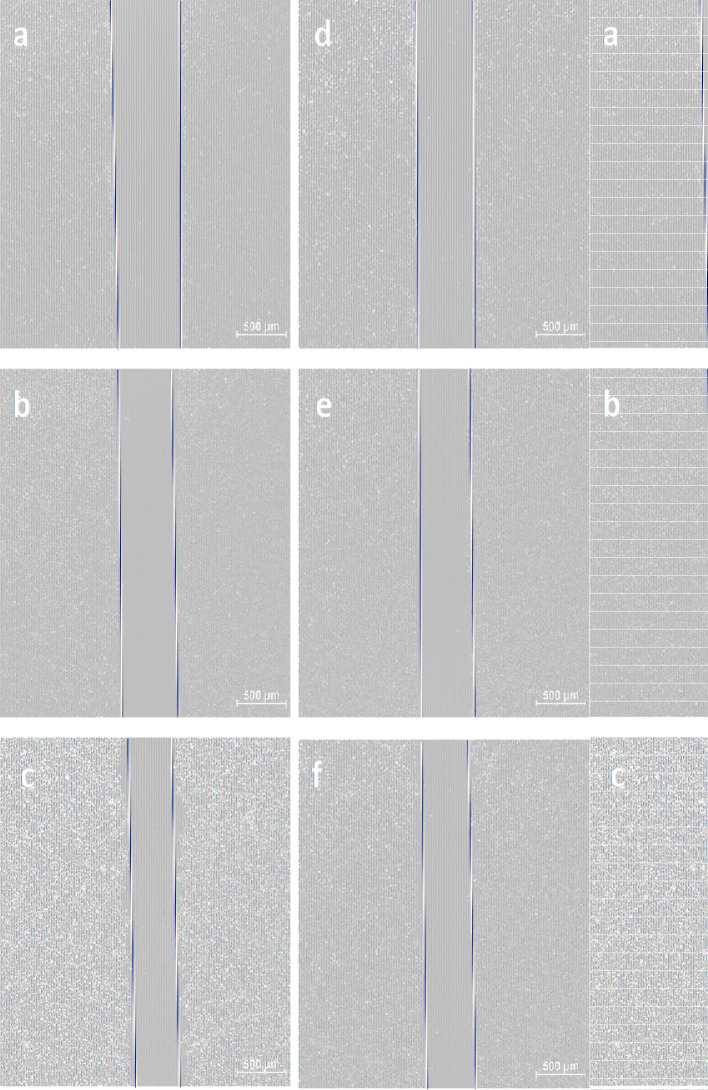
GLA Suppresses Huh7 Cell Migration. Representative images from a wound healing assay showing the migratory capacity of Huh7 cells. **(a-c)** Control group: Huh7 cells cultured in normal medium at 0 h, 12 h, and 24 h, respectively. **(d-f)** Experimental group: Huh7 cells treated with 0.25 mmol/L GLA at 0 h, 12 h, and 24 h, respectively. Red lines delineate the wound edges. Scale bar = 500 µm.

The impact of GLA on Huh7 cell migration was evaluated using a wound healing assay (**Figure 6**). Compared to the scratch width at 0 hours, both the control group (normally cultured Huh7 cells) and the experimental group (Huh7 cells treated with 0.25 mmol/L GLA) showed changes in cell migration distance at 12 hours and 24 hours. However, the experimental group exhibited significantly smaller migration distances at both 12 hours and 24 hours compared to the control group. This observation demonstrates that GLA effectively inhibits the migratory capacity of Huh7 cells, further supporting its inhibitory effect on Huh7 cell activity.

### 3.5 GLA disrupts cytoskeleton and alters cell morphology

**Figure 7:**
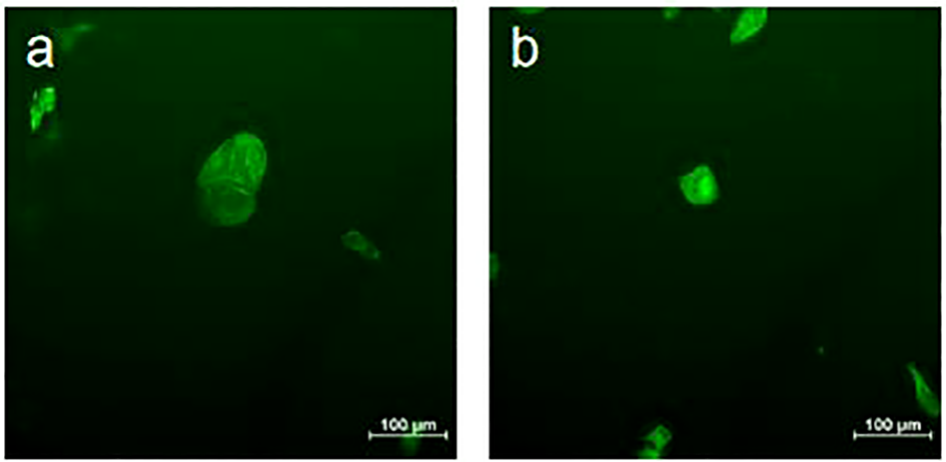
GLA Induces Cytoskeletal Damage in Huh7 Cells.Fluorescence microscopic images showing the cytoskeleton of Huh7 cells. **(a)** Control group: Huh7 cells cultured in normal medium for 24 (h) **(b)** Experimental group: Huh7 cells treated with 0.25 mmol/L γ-Linolenic Acid for 24 (h) Cytoskeleton was stained with phalloidin-FITC (green fluorescence). Scale bar = 100 µm.

Fluorescence microscopy was employed to visualize the cytoskeleton of Huh7 cells following GLA treatment (**Figure 7**). In the control group (**Figure 7a**), Huh7 cells displayed a regular and organized cytoskeletal arrangement with normal morphology. In contrast, Huh7 cells treated with 0.25 mmol/L GLA for 24 hours (**Figure 7b**) exhibited an irregular cytoskeletal arrangement, with structural protrusions, and damaged, bent, and folded structures, causing the overall cell morphology to gradually contract and become more rounded. These findings indicate that GLA has a damaging effect on the Huh7 cell cytoskeleton, which contributes to its inhibitory effect on Huh7 cell activity. The detailed observation of cytoskeletal changes is not merely a descriptive morphological finding but directly links to the observed reduction in cell migration and viability. The cytoskeleton is fundamental for cell shape, adhesion, and movement; thus, its disruption provides a mechanistic explanation for the functional impairments observed in other assays.

### 3.6 GLA induces cell death in Huh7 cells

**Figure 8:**
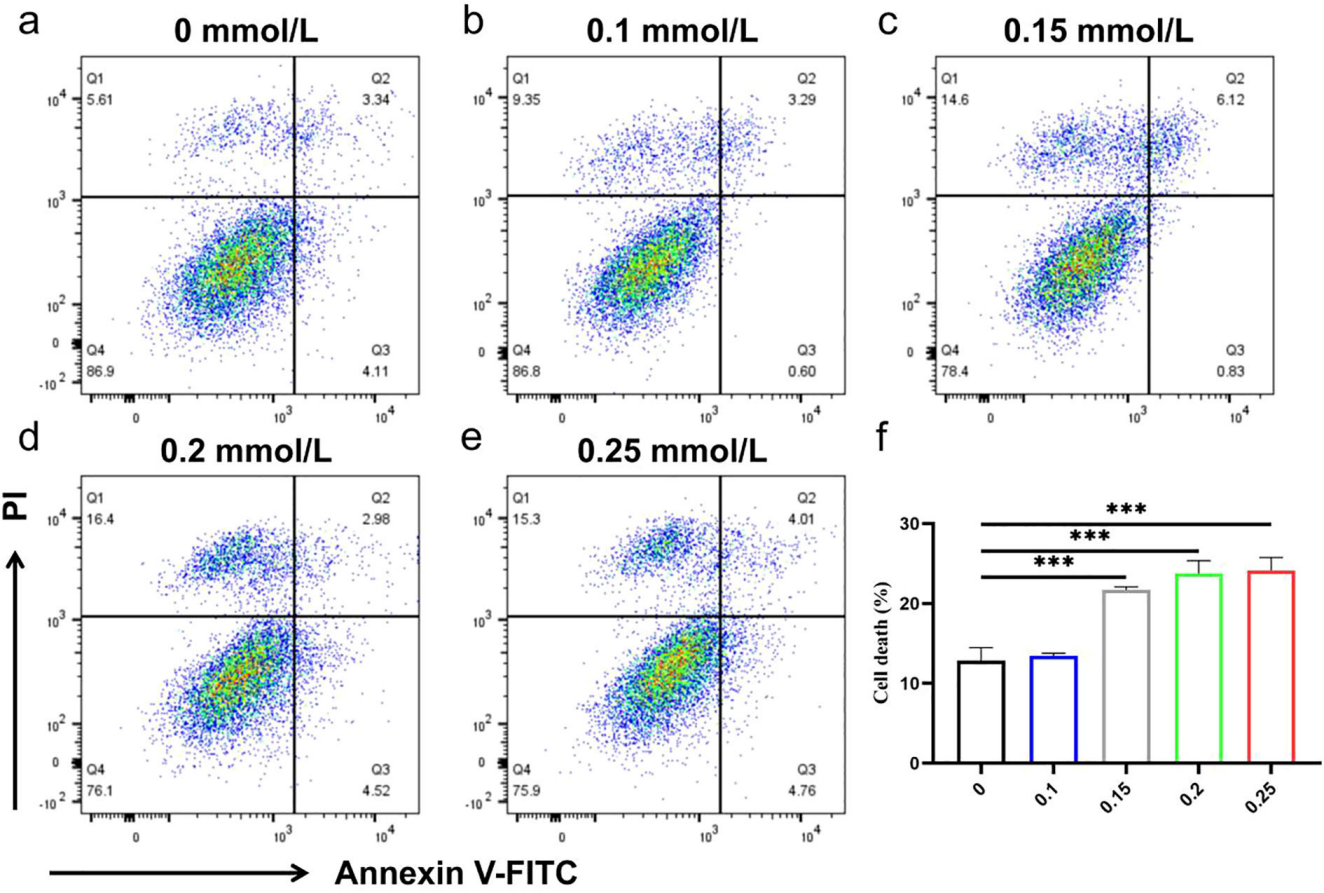
GLA Induces Huh7 Cell Death. Flow cytometry analysis of Huh7 cell death after 24 hours of treatment with various concentrations of GLA. **(a-e)** Representative Annexin V-FITC/PI dot plots for each concentration. Q1: Necrotic cells; Q2: Late apoptotic/Necrotic cells; Q3: Early apoptotic cells; Q4: Live cells. **(f)** Bar graph showing the total percentage of cell death (Q1+Q2+Q3) across different concentrations. Data are presented as mean ± SD. ***p < 0.001 versus control.

The effect of GLA on Huh7 cell death was analyzed by flow cytometry (**Figure 8**). Huh7 cells were treated with GLA at various concentrations (0, 0.1, 0.15, 0.2, 0.25 mM) for 24 hours. The percentage of cell death progressively increased with higher concentrations of GLA, particularly from 0.1 mmol/L to 0.25 mM, when compared to the 0 mmol/L control. This indicates that GLA inhibits Huh7 cell activity by inducing cell death. To further investigate the influence of GLA on cell death, two concentrations below the IC_50_ value (0.2 mmol/L and 0.15 mM) were selected for subsequent experiments. Consistent with the Annexin V/PI profiles, apoptosis-associated protein caspase3 changes support apoptosis as a major mode of cell death under these conditions.

**Figure 9:**
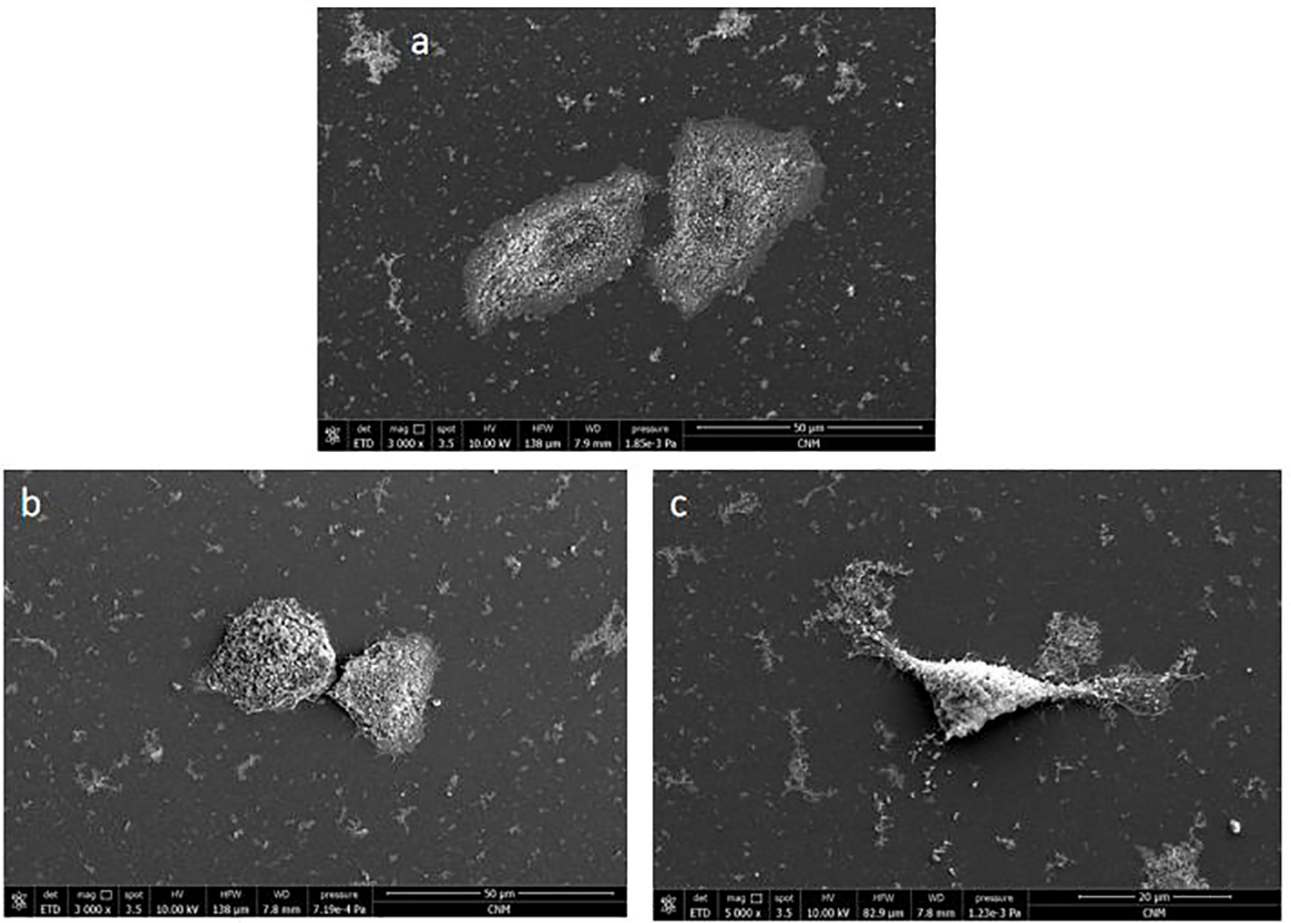
Scanning Electron Microscopy Reveals Morphological Alterations in Huh7 Cells Treated with GLA. **(a)** Control group: Huh7 cells cultured in normal medium for 24 (h) **(b)** Experimental group: Huh7 cells treated with 0.15 mmol/L GLA for 24 (h) **(c)** Experimental group: Huh7 cells treated with 0.2 mmol/L GLA for 24 (h) Scale bar = 50 µm for **(a)** and **(b)**, 20 µm for **(c)**.

SEM was used to characterize the morphological changes in Huh7 cells after GLA treatment (**Figure 9**). In the control group (**Figure 9a**), Huh7 cells displayed normal morphology with intact structures. In contrast, cells treated with 0.15 mmol/L (**Figure 9b**) and 0.2 mmol/L (**Figure 9c**) GLA exhibited significant structural deformation, including prominent nuclear protrusion, clear breakage and damage to the cell matrix and pseudopods. Some cellular structures appeared to detach from the coverslip, and these effects became more pronounced with increasing GLA concentration. These findings confirm that GLA damages the morphological structure of Huh7 cells, thereby affecting their activity. This further substantiates that GLA inhibits Huh7 cell activity. SEM provides high-resolution insights into the surface morphology of cells, complementing the internal cytoskeletal observations. The detailed visualization of nuclear protrusion, significant damage to the cell matrix and pseudopods, and partial detachment from the substrate indicates severe and widespread cellular damage, affecting external structures and adhesion. This comprehensive damage profile further underscores the potent cytotoxic effects of GLA beyond just internal structural changes.

**Figure 10:**
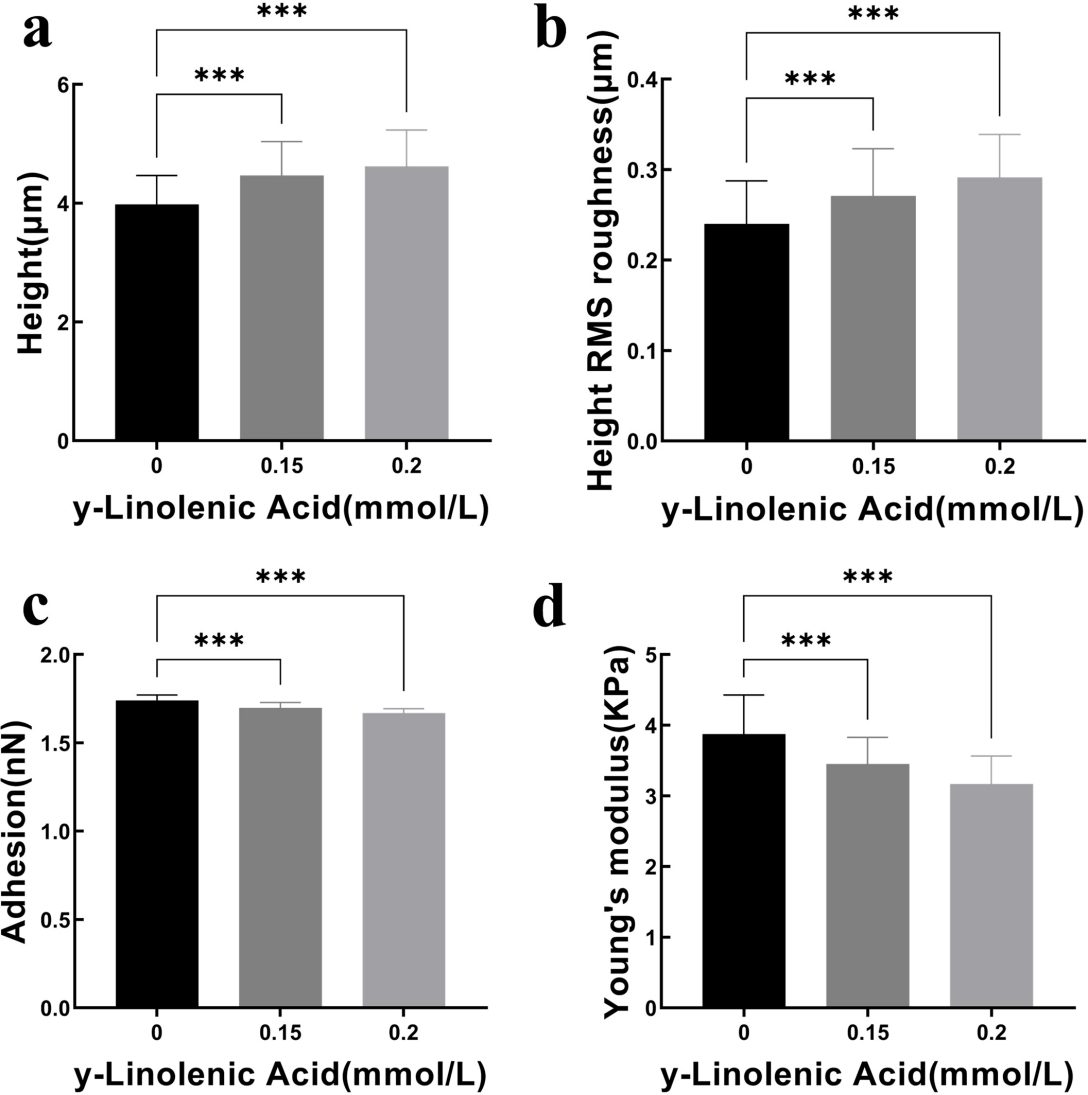
Quantitative Analysis of Huh7 Cell Biomechanical Properties by AFM. Bar graphs illustrating the changes in biomechanical properties of Huh7 cells after 24 hours of treatment with GLA. **(a)** Cell Height (µm). **(b)** Height RMS roughness (µm). **(c)** Adhesion (nN). **(d)** Young’s modulus (KPa). Data are presented as mean ± standard deviation. ***p < 0.001 indicates significant differences compared to the control.

**Figure 11:**
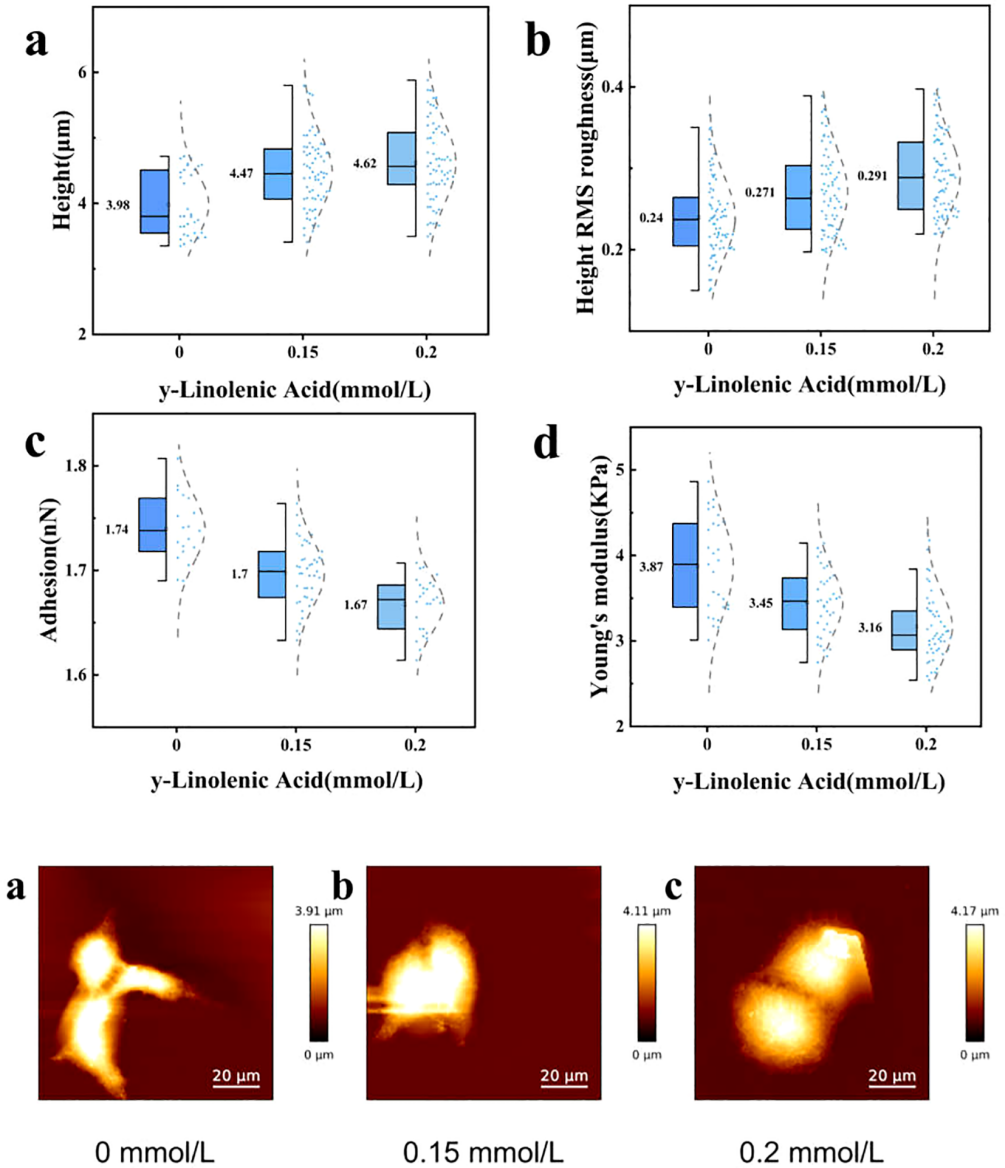
Distribution of Huh7 Cell Biomechanical Properties by AFM. Violin plots showing the distribution of biomechanical properties of Huh7 cells after 24 hours of treatment with GLA. **(a)** Cell Height (µm). **(b)** Height RMS roughness (µm). **(c)** Adhesion (nN). **(d)** Young’s modulus (KPa). Box plots within the violins indicate median and interquartile ranges.

Atomic Force Microscopy (AFM) was employed to quantitatively assess the biomechanical properties of Huh7 cells after γ-Linolenic Acid treatment (**Figures 10**, **11**). AFM results demonstrated that Huh7 cell height increased from 3.98 ± 0.49 μm in the control group to 4.47 ± 0.57 μm (***p < 0.001) and 4.62 ± 0.61 μm (***p < 0.001) after treatment with 0.15 mmol/L and 0.2 mmol/L GLA, respectively (**Figures 10a**, **11a**). Simultaneously, the surface root mean square (RMS) roughness slowly increased from 0.24 ± 0.05 μm to 0.27 ± 0.05 μm (***p < 0.001) and 0.29 ± 0.05 μm (***p < 0.001) (**Figures 10b**, **11b**). These changes suggest that GLA induces cellular swelling and promotes surface irregularities. Conversely, cell adhesion decreased from 1.74 ± 0.03 nN in the control group to 1.70 ± 0.03 nN (***p < 0.001) and 1.67 ± 0.03 nN (***p < 0.001) (**Figures 10c**, **11c**). Correspondingly, the Young’s modulus of the cells also showed a decreasing trend, from 3.87 ± 0.55 kPa in the control group to 3.44 ± 0.38 kPa (***p < 0.001) and 3.16 ± 0.40 kPa (***p < 0.001) (**Figures 10d**, **11d**). These findings indicate that GLA affects the morphology and mechanical properties of Huh7 cells, consistent with the SEM characterization and cytoskeleton imaging results. The quantitative AFM data, showing increased cell height and roughness, coupled with decreased adhesion and Young’s modulus, directly indicates a loss of cellular rigidity and integrity. This biomechanical weakening represents a critical, quantifiable aspect of cell damage that directly supports and explains the observed morphological changes (from SEM and cytoskeleton imaging) and functional impairments (such as reduced migration and viability). This adds a novel and precise dimension to understanding the multi-faceted anti-cancer mechanism of GLA.

**Figure 12:**
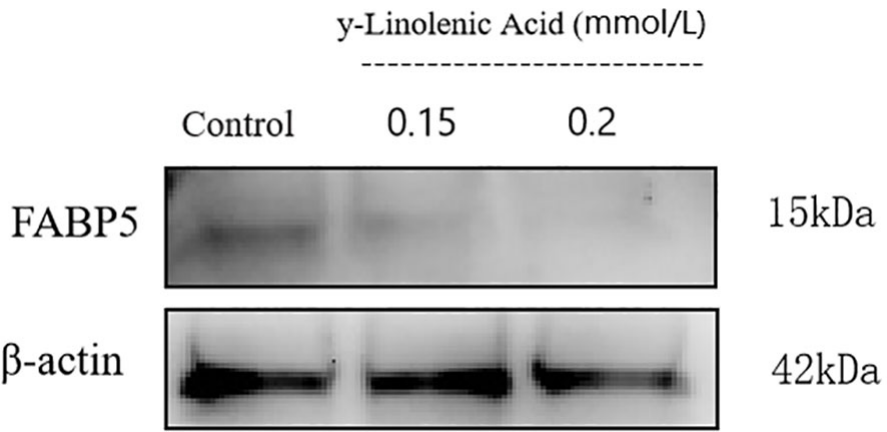
Western blot analysis of FABP5 expression in Huh7 cells treated with GLA for 24 h. β-actin was used as a loading control.

Western blot analysis was performed to examine the effect of γ-linolenic acid (GLA) on FABP5 protein expression in Huh7 cells (**Figure 12**). FABP5 expression was significantly modulated by GLA in a concentration-dependent manner. Compared with the control group, GLA treatment resulted in a progressive reduction in FABP5 protein levels with increasing concentrations. Notably, 0.2 mmol/L GLA produced a marked inhibitory effect on FABP5 expression. .

Together, the dose-dependent downregulation of FABP5 observed across multiple HCC cell lines provides experimental evidence supporting a functional association between GLA and FABP5. When considered alongside the computational binding predictions, these results suggest that FABP5 modulation is likely involved in the anticancer activity of GLA, although additional regulatory mechanisms may also contribute to its overall effects.

### 3.7 Molecular docking analysis

**Figure 13:**
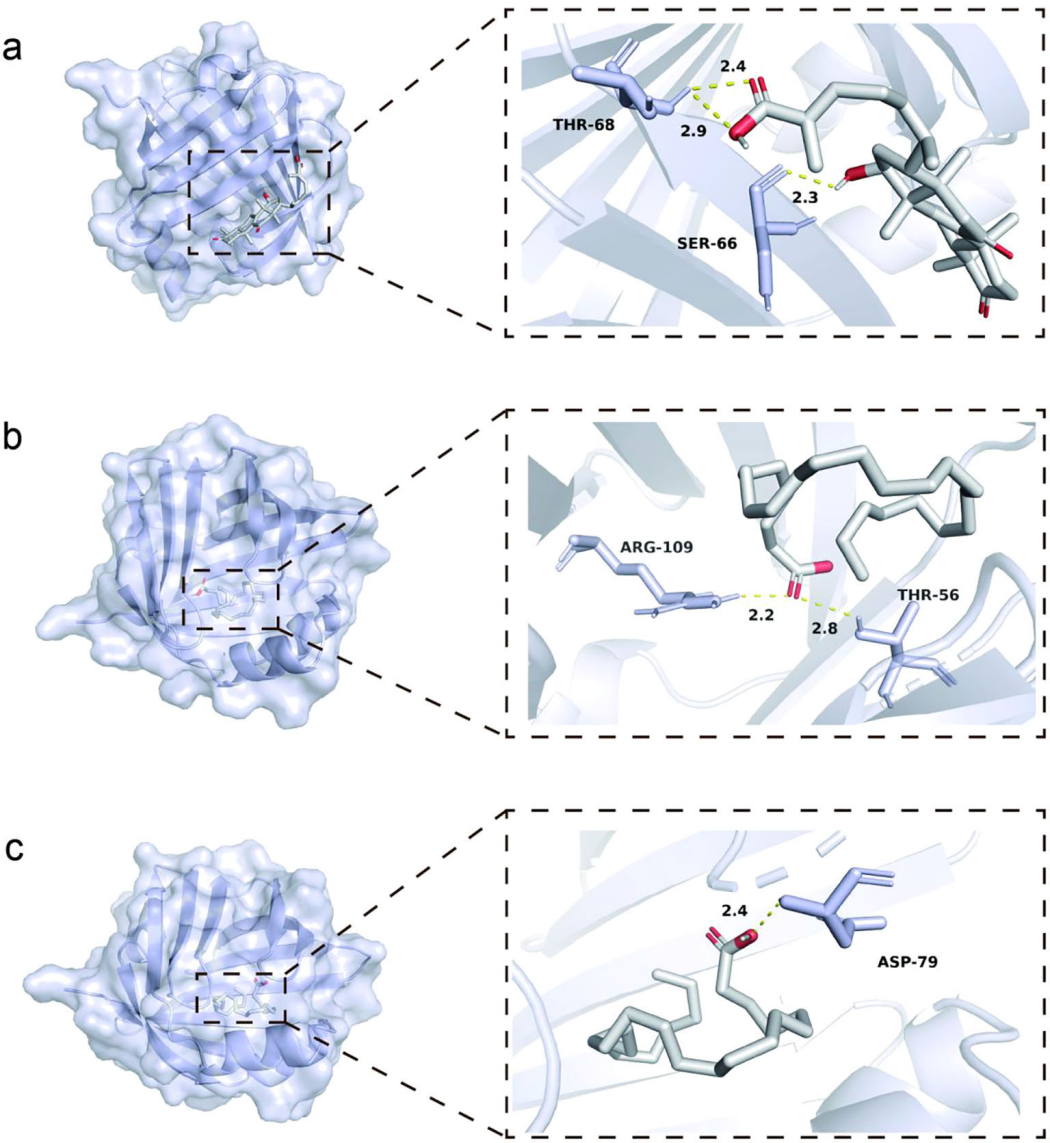
Docking conformations and structures of three active molecules. **(a)** Ganolucidic Acid E; **(b)** Tetracosanoic acid; **(c)** Linolenic acid.

The docking results showed that γ-linolenic acid formed stable interactions with key residues in the FABP5 binding pocket (**Figure 13**).

## 4 Discussion

This study employed an integrated computational and experimental framework to identify gamma-linolenic acid (GLA) as a functional modulator of fatty acid binding protein 5 (FABP5) in hepatocellular carcinoma (HCC). Machine learning–based virtual screening and molecular docking predicted GLA as a promising FABP5-interacting compound with favorable drug-likeness properties and stable binding within the FABP5 pocket. These computational findings were supported by experimental validation in Huh7 cells, where GLA treatment resulted in a dose-dependent reduction in cell viability and migration, accompanied by pronounced cytoskeletal disruption and induction of cell death. Together, these results indicate that GLA exerts multi-level inhibitory effects on HCC cells through modulation of FABP5-associated processes.

FABP5 is increasingly recognized as a key regulator of lipid metabolism and tumor aggressiveness in HCC. Compared with normal hepatocytes, HCC cells frequently exhibit elevated FABP5 expression, which supports enhanced fatty acid trafficking, metabolic reprogramming, and migratory capacity. In this context, the observed downregulation of FABP5 protein expression following GLA treatment provides a mechanistic link between target modulation and the anti-tumor phenotypes observed in Huh7 cells. Cancer cells are particularly dependent on FABP5-mediated lipid signaling, which may explain their heightened sensitivity to FABP5 inhibition. By contrast, naturally occurring polyunsaturated fatty acids such as GLA are generally well tolerated by normal hepatic cells at physiological concentrations, suggesting a degree of functional selectivity. Nevertheless, further evaluation using normal hepatocyte models will be essential to define the therapeutic window and confirm safety.

At the molecular level, linolenic acid functions primarily as a ligand that binds to FABP5 and modulates its activity rather than directly suppressing FABP5 gene transcription. Binding of GLA to FABP5 is expected to alter intracellular lipid trafficking and downstream lipid-sensitive signaling pathways, including those involving peroxisome proliferator-activated receptors. Such perturbations may secondarily influence FABP5 expression through feedback regulatory mechanisms. In the present study, the reduction in FABP5 protein levels observed by Western blot likely reflects functional inhibition and altered lipid signaling dynamics rather than direct transcriptional repression. Future transcriptomic and promoter-level studies will be necessary to clarify the long-term regulatory consequences of FABP5–ligand interactions.

Beyond its canonical role as a lipid chaperone, FABP5 has been implicated in non-canonical functions that contribute to tumor cell structure and motility. Increasing evidence suggests that FABP5 influences cytoskeletal organization, cell adhesion, and mechanical properties, which are critical for cancer cell migration and invasion. Consistent with these roles, GLA-treated Huh7 cells exhibited severe cytoskeletal disorganization, irregular morphology, loss of pseudopodia, reduced adhesion, and decreased Young’s modulus, as revealed by high-resolution SEM and AFM analyses. These structural and mechanical alterations provide a plausible explanation for the impaired migratory capacity and reduced viability observed following GLA treatment, further supporting FABP5 as a central mediator linking lipid metabolism to cellular architecture in HCC.

## 5 Conclusion

In conclusion, this study identifies gamma-linolenic acid (GLA) as a promising natural compound that targets FABP5 in hepatocellular carcinoma. Through an integrated machine learning, molecular docking, and experimental validation approach, we demonstrate that GLA interacts with FABP5, downregulates its protein expression, and disrupts FABP5-associated lipid signaling, cytoskeletal integrity, and mechanical stability in Huh7 cells. These effects culminate in reduced cell viability and migration, highlighting the functional importance of FABP5 in HCC progression. While further *in vivo* validation and safety assessment in normal hepatocytes are required, our findings support FABP5 as a biologically relevant therapeutic target and underscore the potential of GLA as a natural compound–based strategy for HCC treatment.

## Data Availability

The original contributions presented in the study are included in the article/**Supplementary Material**. Further inquiries can be directed to the corresponding author.
