# Supplementary material for: Identification and validation of γ-Linolenic acid as a natural FABP5 inhibitor in hepatocellular carcinoma through deep learning and experimental approaches
Source: Front Immunol. 2026 Jan 28;17:1700347. doi: 10.3389/fimmu.2026.1700347 (PMC12891133; doi:10.3389/fimmu.2026.1700347)
Supplement: Supplementary file 1 [file Table1.docx]

**Table 1.** Results of MM-PBSA

| System | Ganolucidic Acid E | Tetracosanoic acid | Linolenic acid |
| --- | --- | --- | --- |
| ΔEvdW (kcal/mol) | −32.50 ± 2.11 | −13.60 ± 3.10 | −28.50 ± 2.30 |
| ΔEele (kcal/mol) | −17.80 ± 1.75 | −3.05 ± 2.10 | −18.50 ± 3.20 |
| ΔGgas (kcal/mol) | −50.30 ± 2.84 | −16.65 ± 3.70 | −47.00 ± 4.20 |
| ΔGsolv (kcal/mol) | 32.50 ± 2.40 | 10.10 ± 2.95 | 15.50 ± 3.60 |
| ΔGtotal (kcal/mol) | −17.80 ± 1.22 | −6.55 ± 1.55 | −31.50 ± 1.80 |
