# Supplementary material for: Identification and validation of γ-Linolenic acid as a natural FABP5 inhibitor in hepatocellular carcinoma through deep learning and experimental approaches
Source: Front Immunol. 2026 Jan 28;17:1700347. doi: 10.3389/fimmu.2026.1700347 (PMC12891133; doi:10.3389/fimmu.2026.1700347)

Supplementary Figure 1. γ-Linolenic Acid (GLA) Induces Caspase-3 Activation in HCC Cells. Western blot analysis of Caspase-3 expression in Huh7 and HepG2 cells. Cells were treated with GLA (0.2 and 0.4 mM). The reduction of pro-caspase-3 and appearance of cleavage bands indicate the activation of apoptotic pathways in both cell lines. β-actin was used as a loading control.


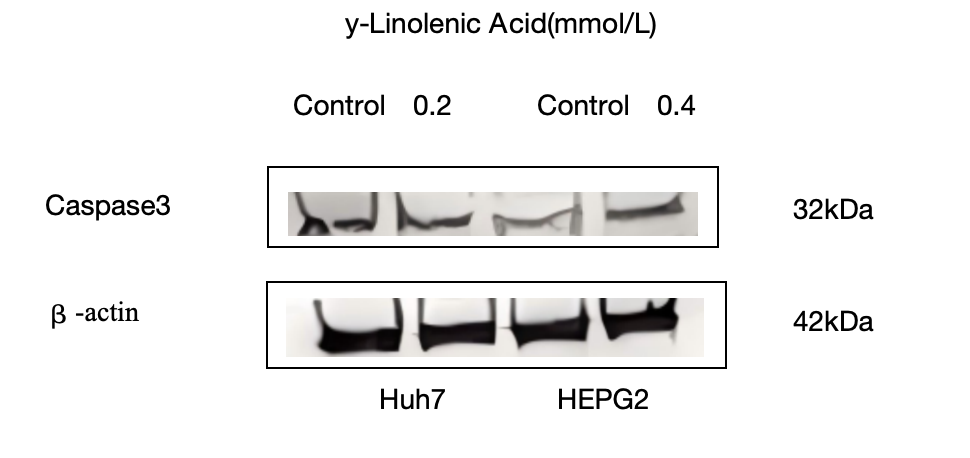


Supplementary Figure 2.

Effect of γ-linolenic acid (GLA) on cell viability in HepG2 cells. HepG2 cells were treated with increasing concentrations of GLA (0.3–0.5 mmol/L), and cell viability was assessed using a cell viability assay. Results are expressed as a percentage relative to the control group. GLA treatment resulted in a significant, dose-dependent reduction in cell viability. Data are presented as mean ± SD (n = 5). Statistical significance was determined compared with the control group (**** p < 0.0001).


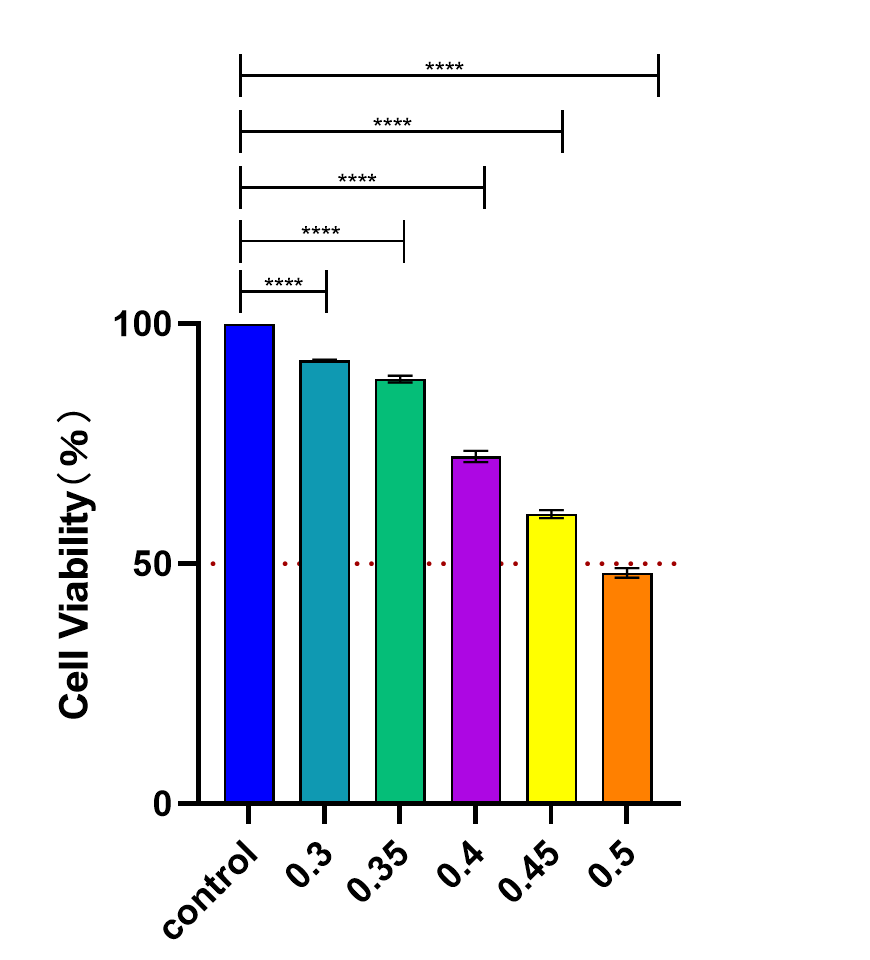

Supplement: Supplementary file 2 [file Table2.docx]
