# Supplementary figures and images for: Identification and validation of γ-Linolenic acid as a natural FABP5 inhibitor in hepatocellular carcinoma through deep learning and experimental approaches
Source: Front Immunol. 2026 Jan 28;17:1700347. doi: 10.3389/fimmu.2026.1700347 (PMC12891133; doi:10.3389/fimmu.2026.1700347)

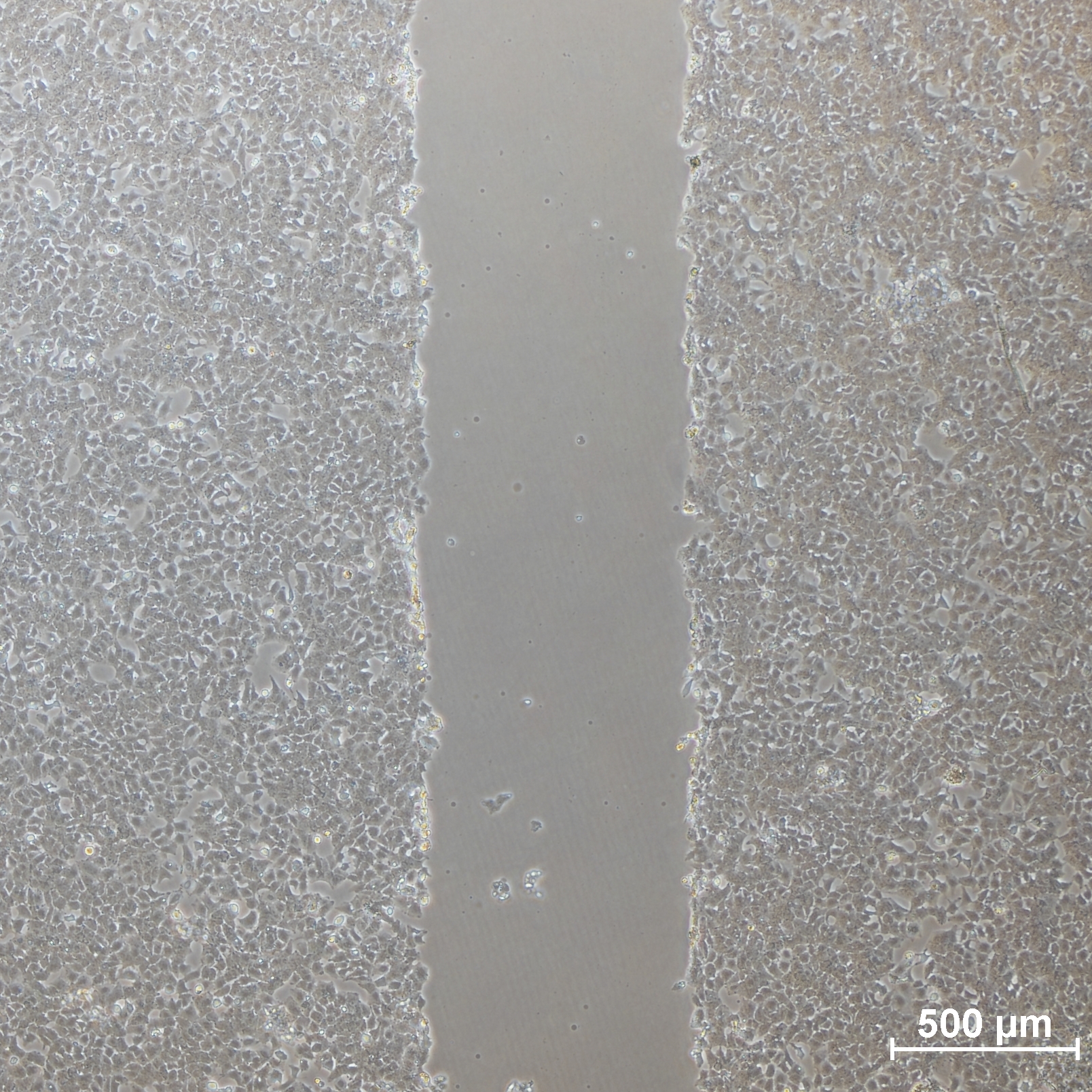

Supplement: Supplementary file 5 [file DataSheet3.zip › scratch width 0h/0-1/1.jpg]

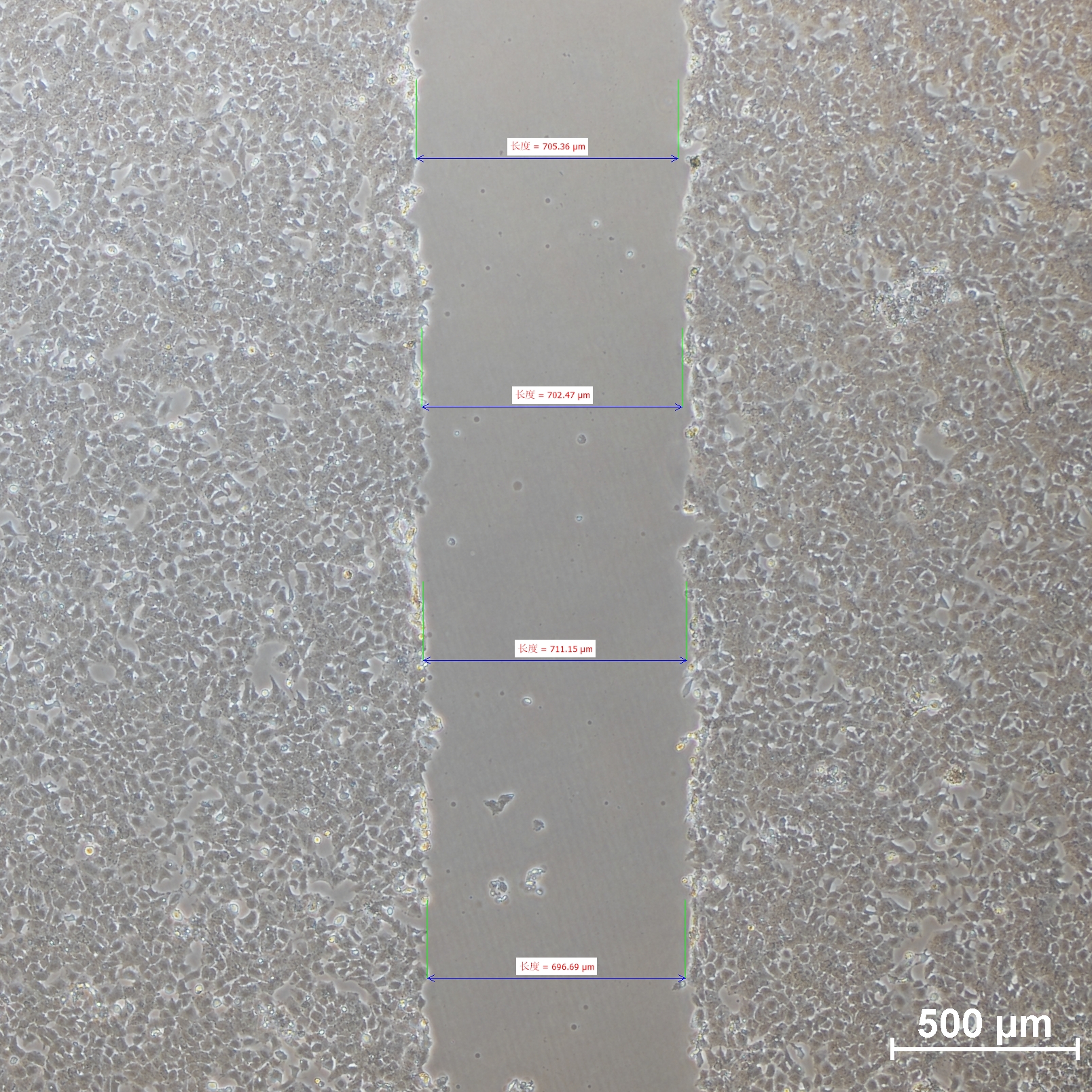

Supplement: Supplementary file 5 [file DataSheet3.zip › scratch width 0h/0-1/2.jpg]

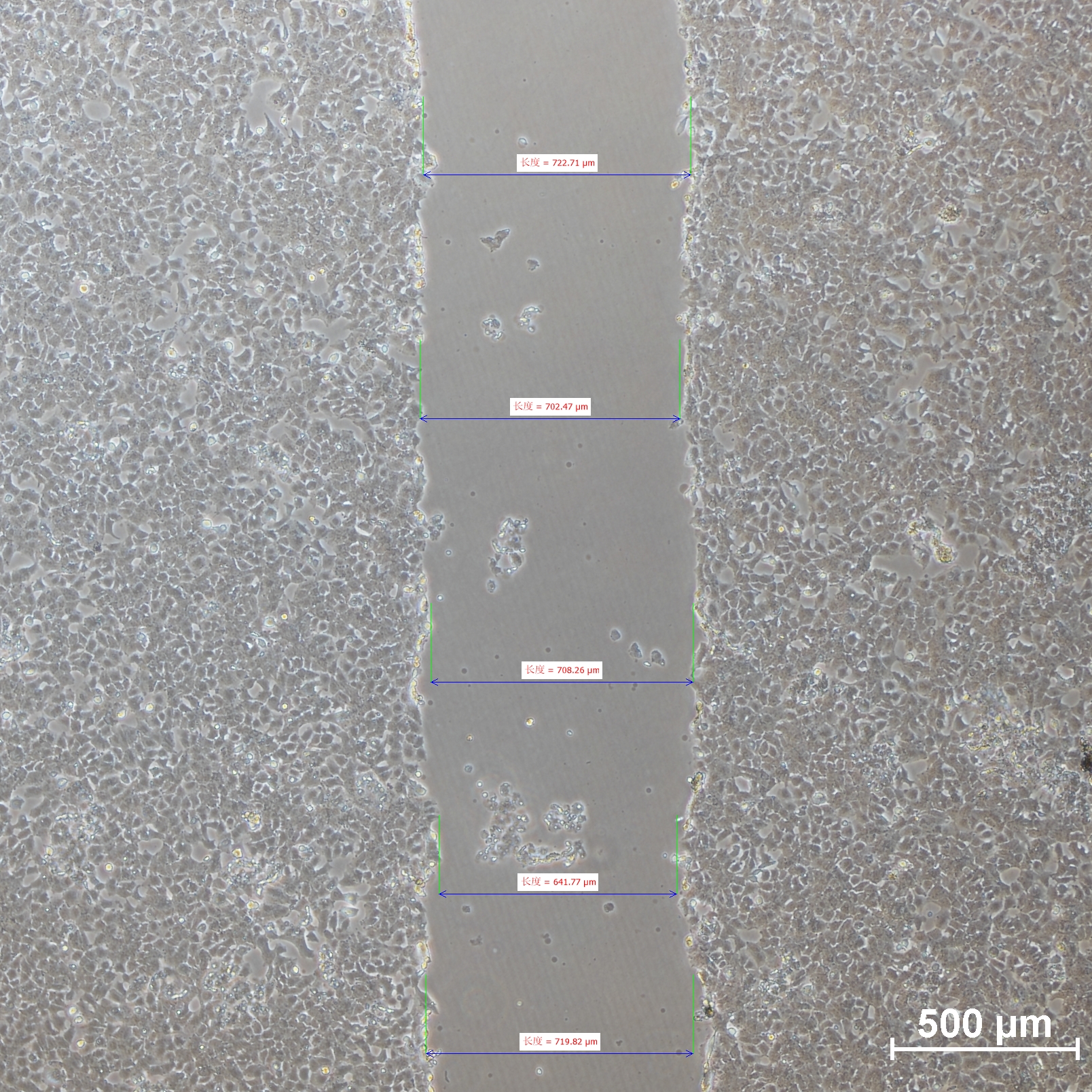

Supplement: Supplementary file 5 [file DataSheet3.zip › scratch width 0h/0-1/3.jpg]

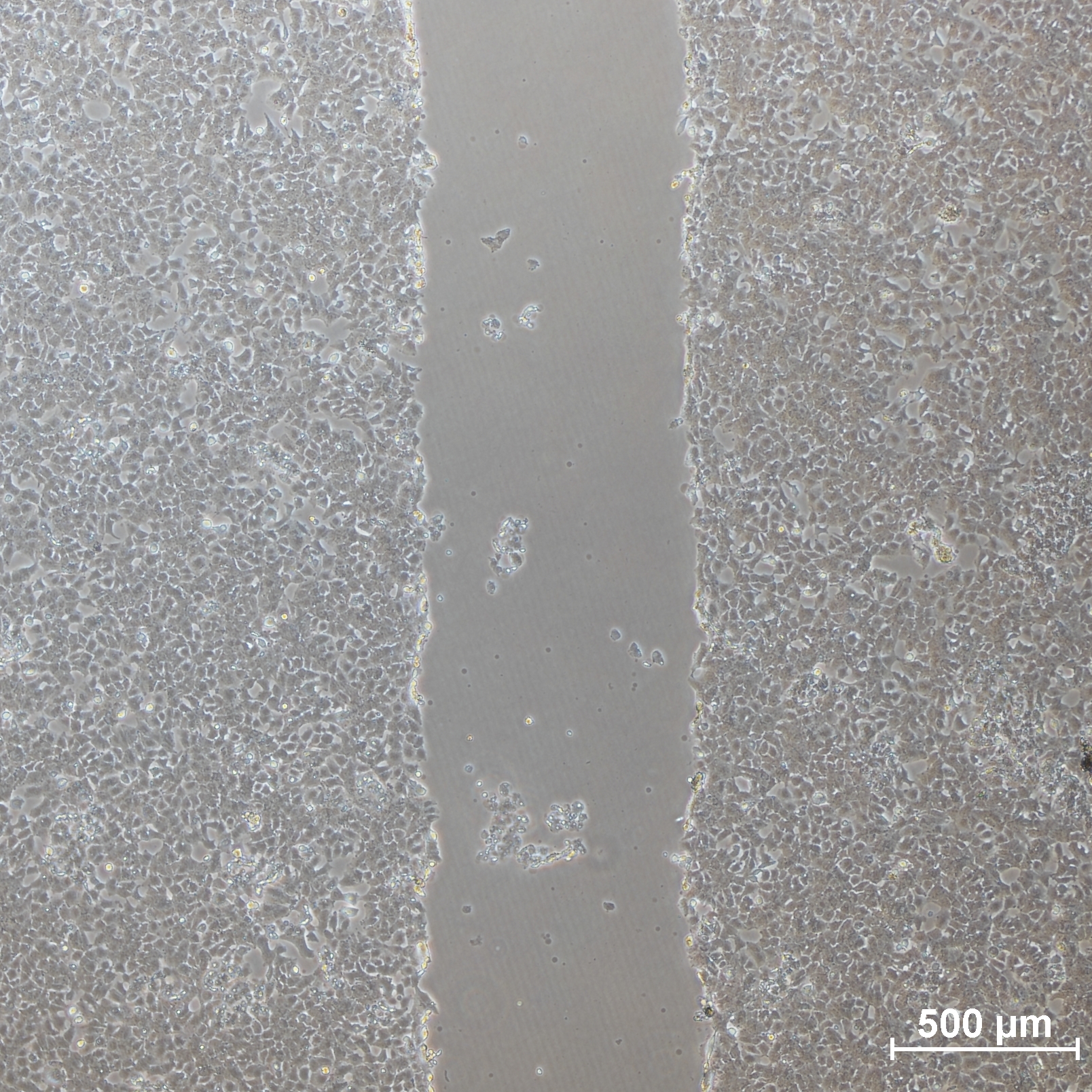

Supplement: Supplementary file 5 [file DataSheet3.zip › scratch width 0h/0-1/4.jpg]

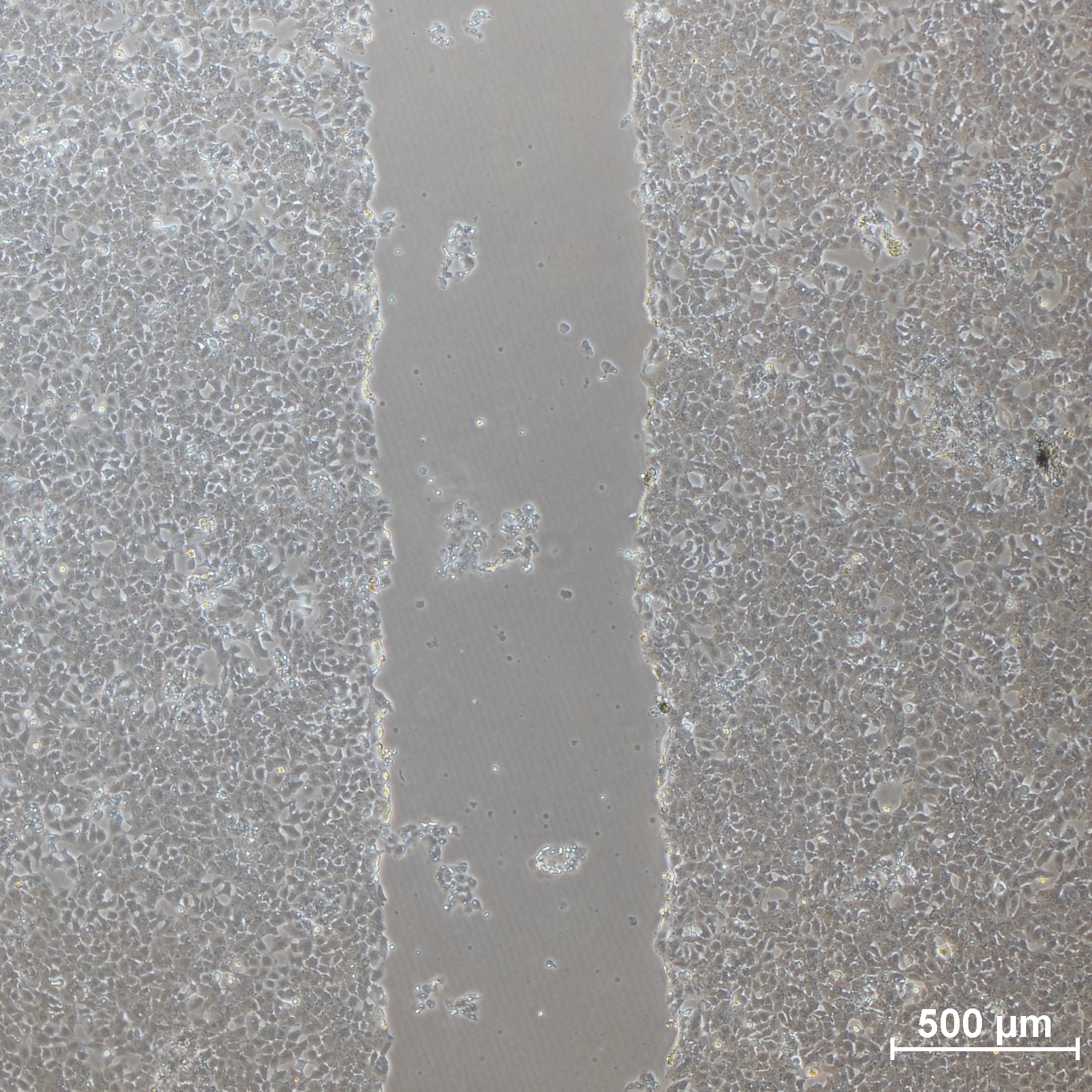

Supplement: Supplementary file 5 [file DataSheet3.zip › scratch width 0h/0-1/5.jpg]

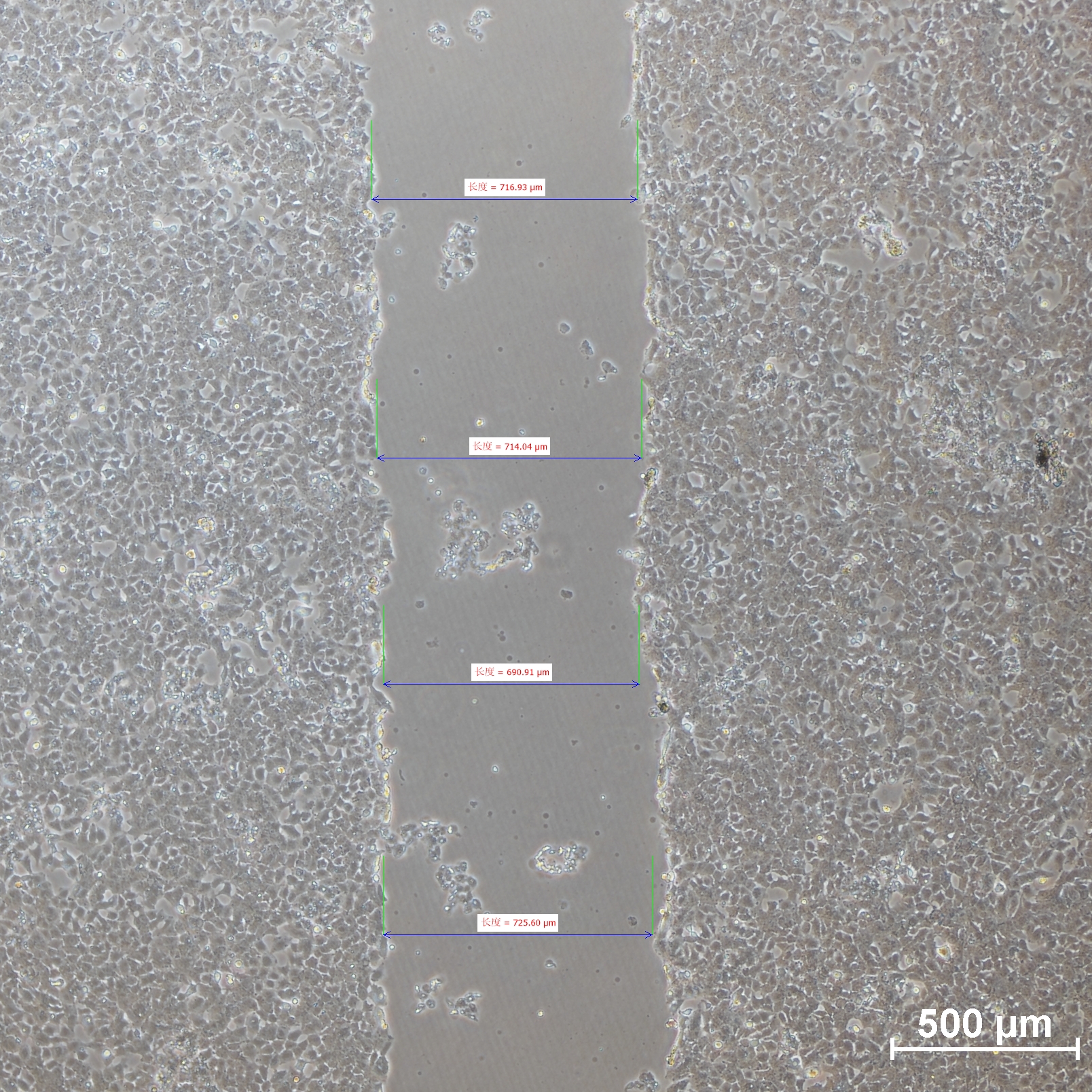

Supplement: Supplementary file 5 [file DataSheet3.zip › scratch width 0h/0-1/6.jpg]

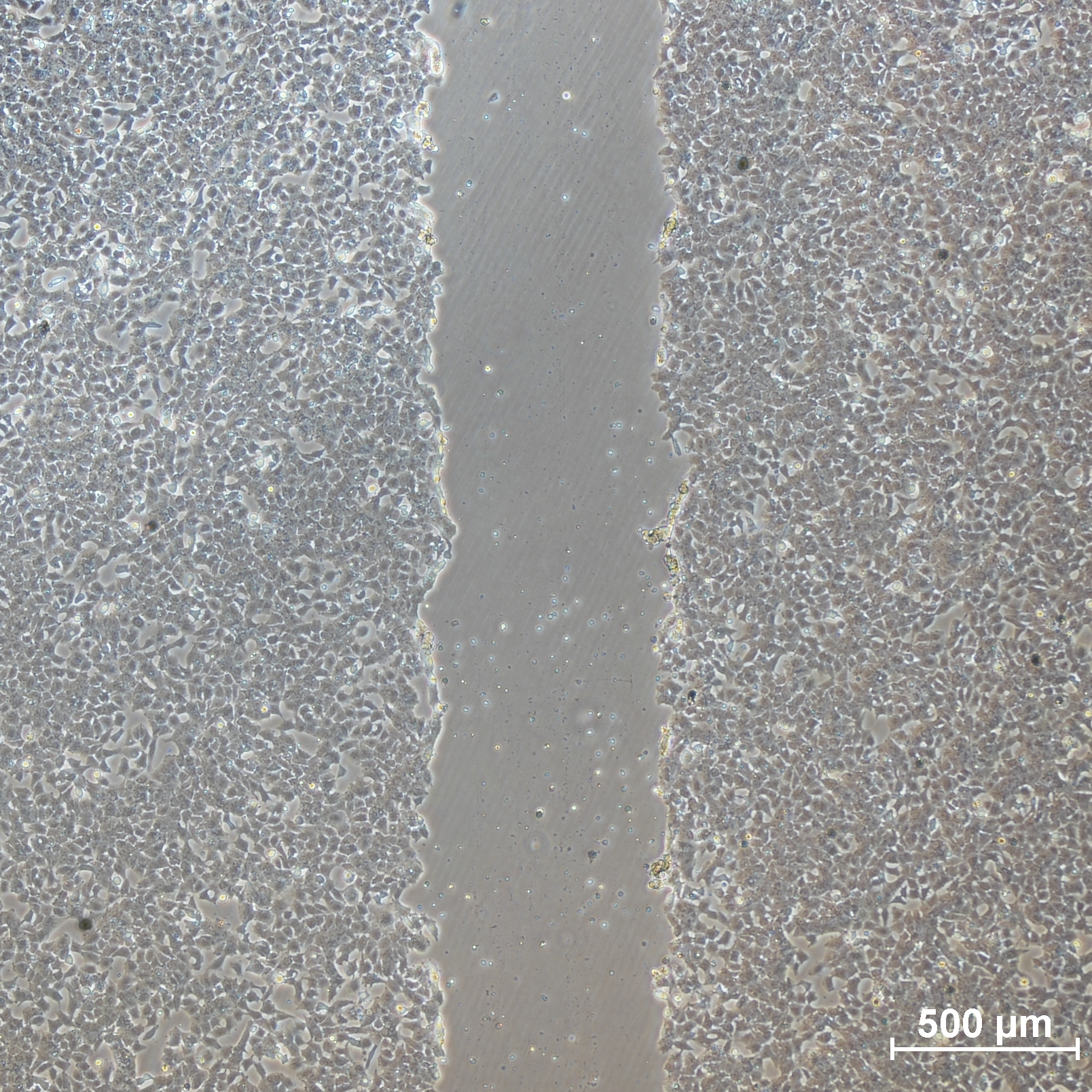

Supplement: Supplementary file 5 [file DataSheet3.zip › scratch width 0h/0.25-1/1.jpg]

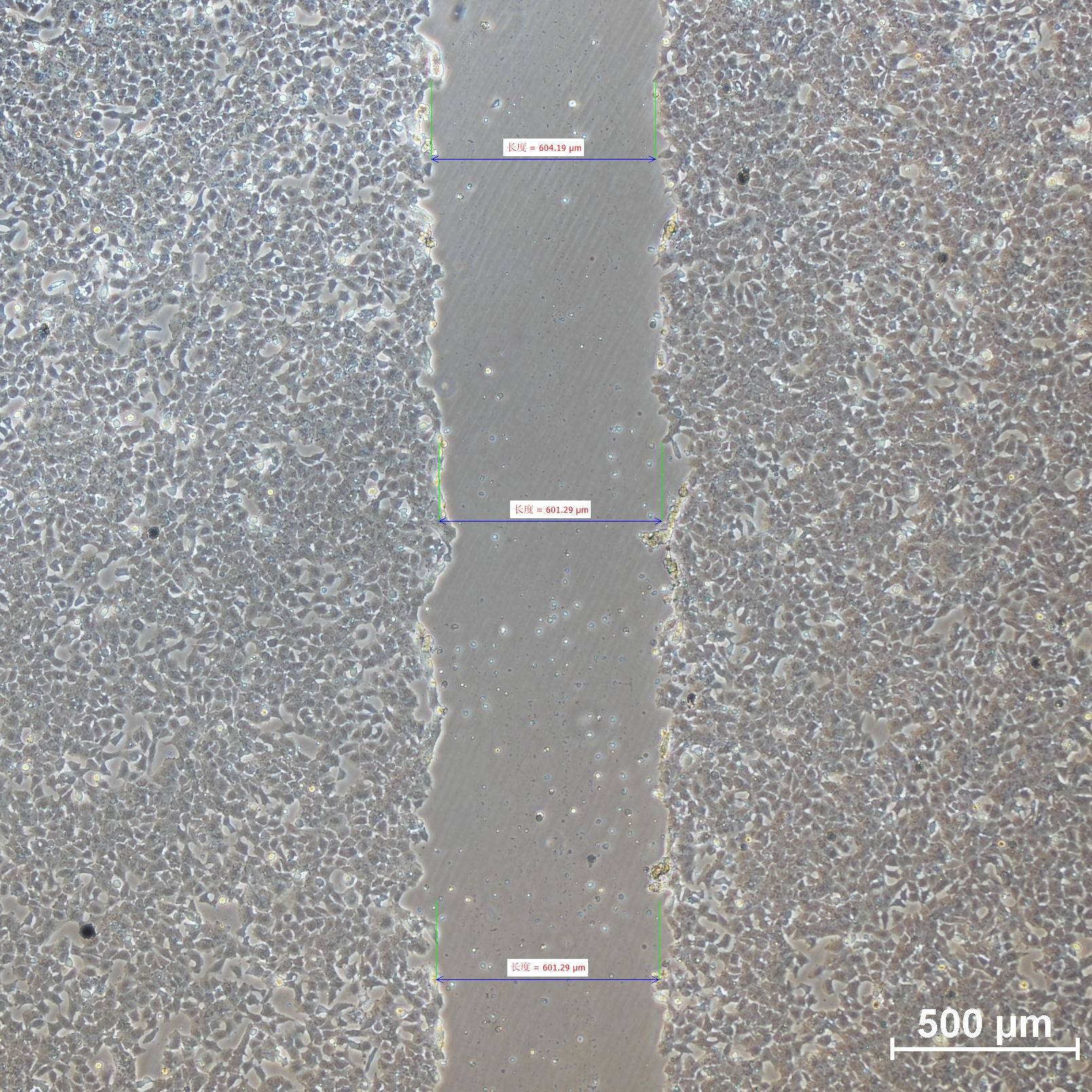

Supplement: Supplementary file 5 [file DataSheet3.zip › scratch width 0h/0.25-1/2.jpg]

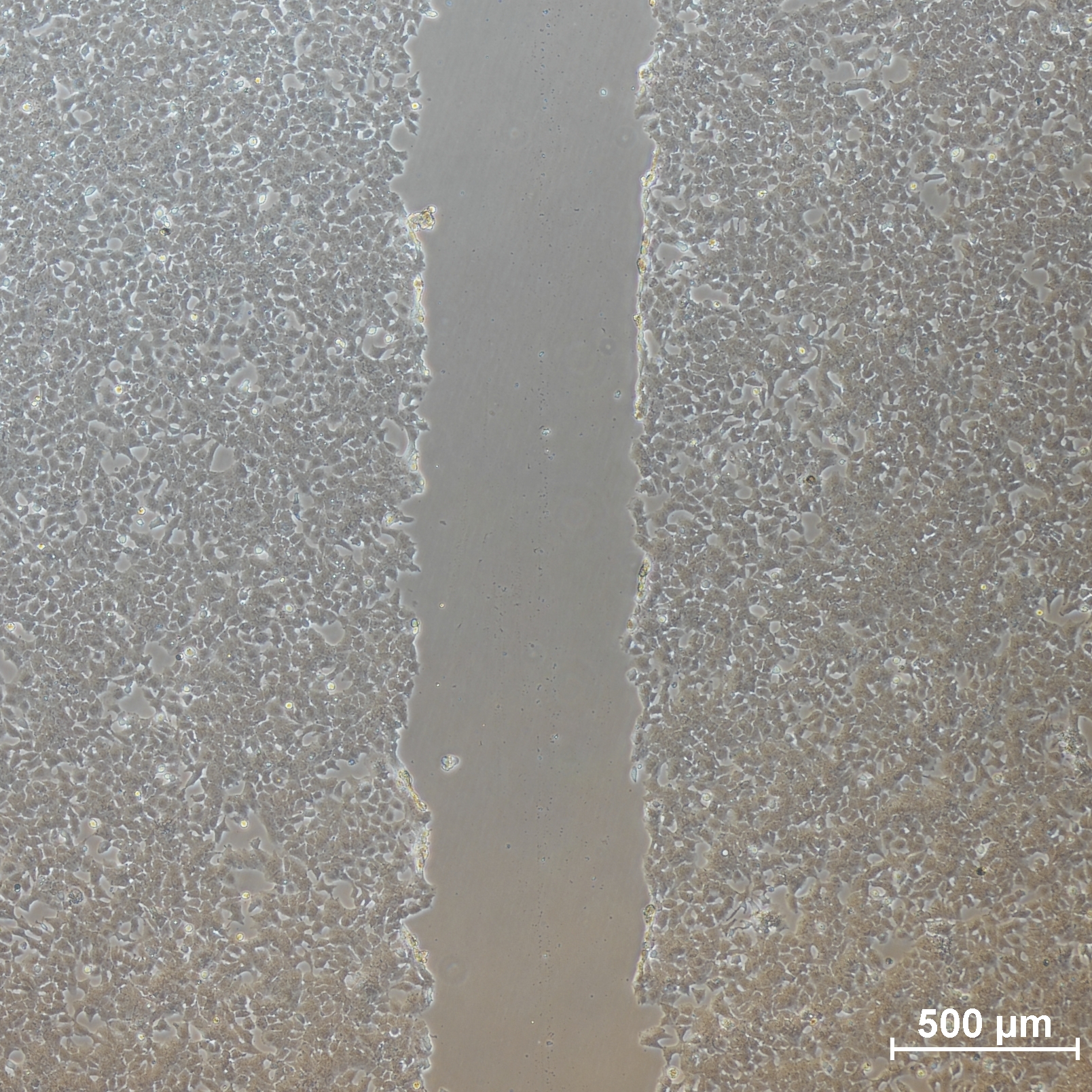

Supplement: Supplementary file 5 [file DataSheet3.zip › scratch width 0h/0.25-1/3.jpg]

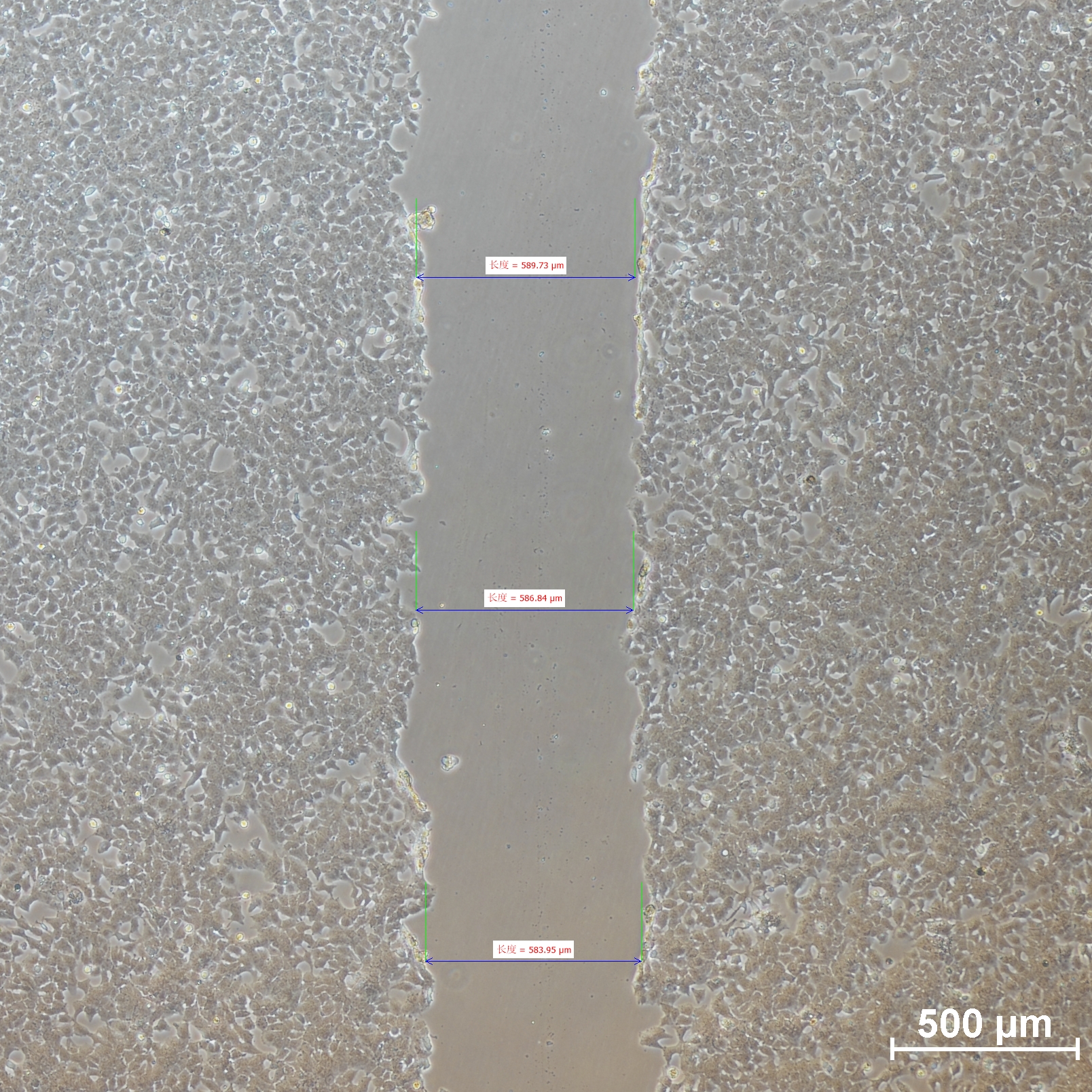

Supplement: Supplementary file 5 [file DataSheet3.zip › scratch width 0h/0.25-1/4.jpg]

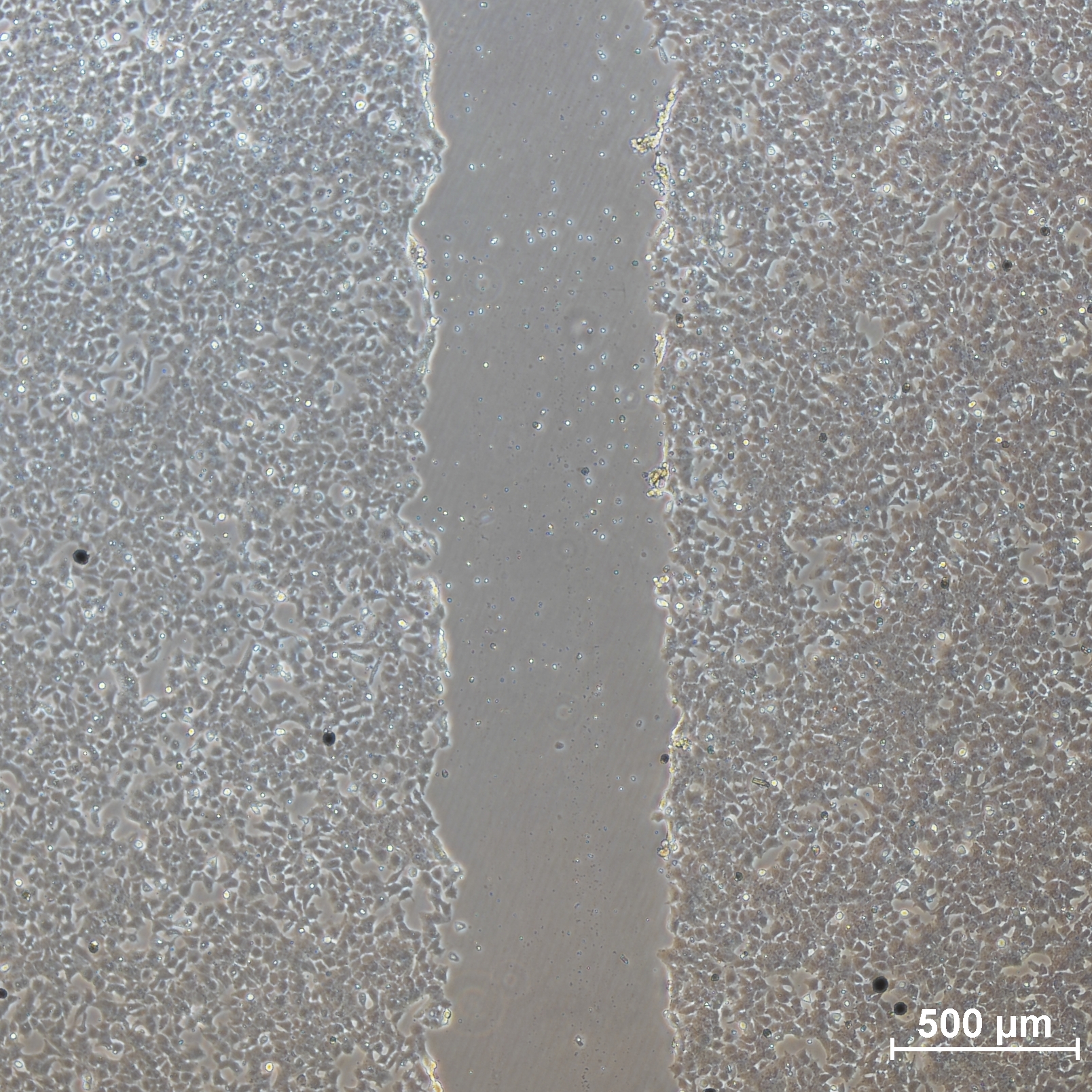

Supplement: Supplementary file 5 [file DataSheet3.zip › scratch width 0h/0.25-1/5.jpg]

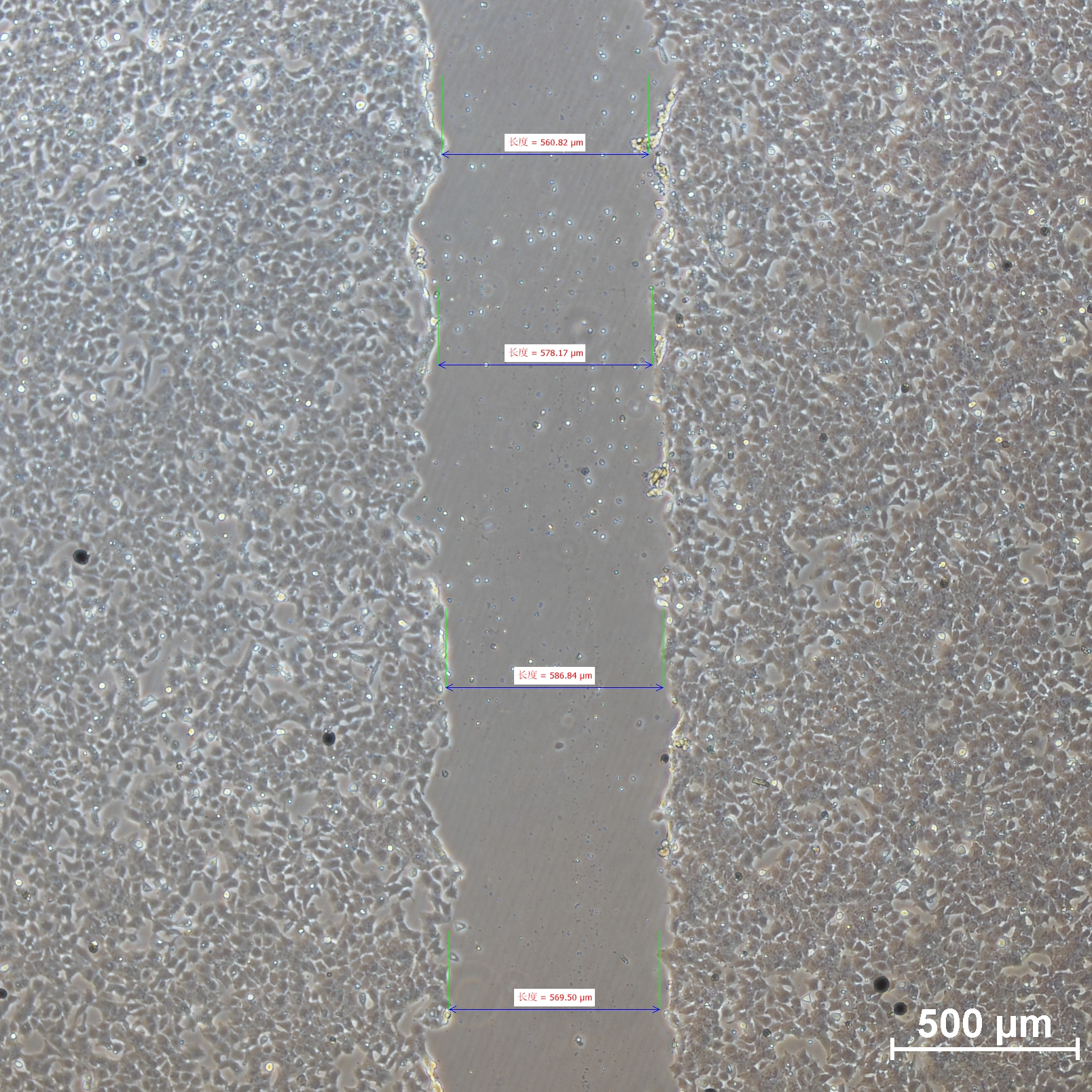

Supplement: Supplementary file 5 [file DataSheet3.zip › scratch width 0h/0.25-1/6.jpg]

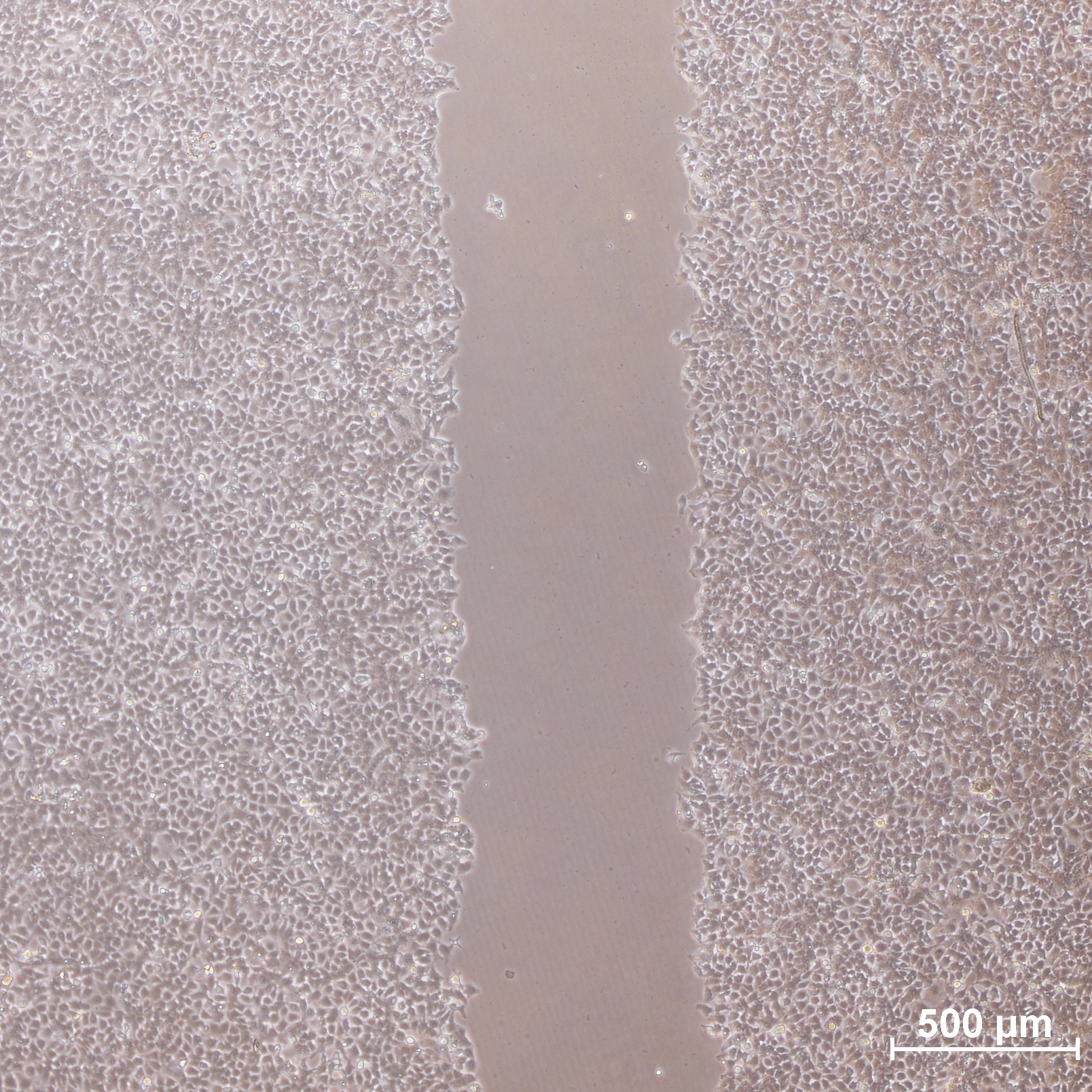

Supplement: Supplementary file 6 [file DataSheet4.zip › scratch width 12h/0-1/1.jpg]

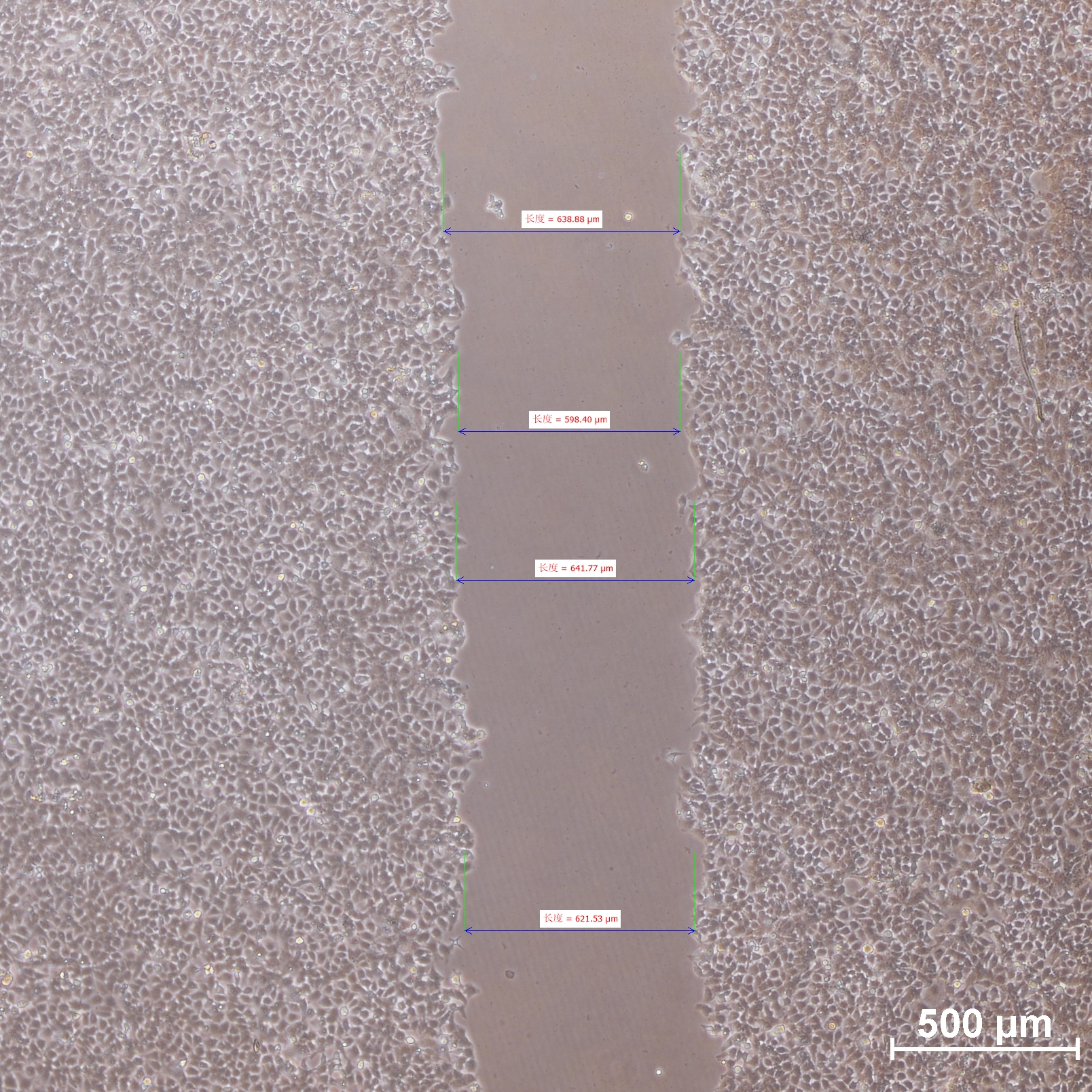

Supplement: Supplementary file 6 [file DataSheet4.zip › scratch width 12h/0-1/2.jpg]

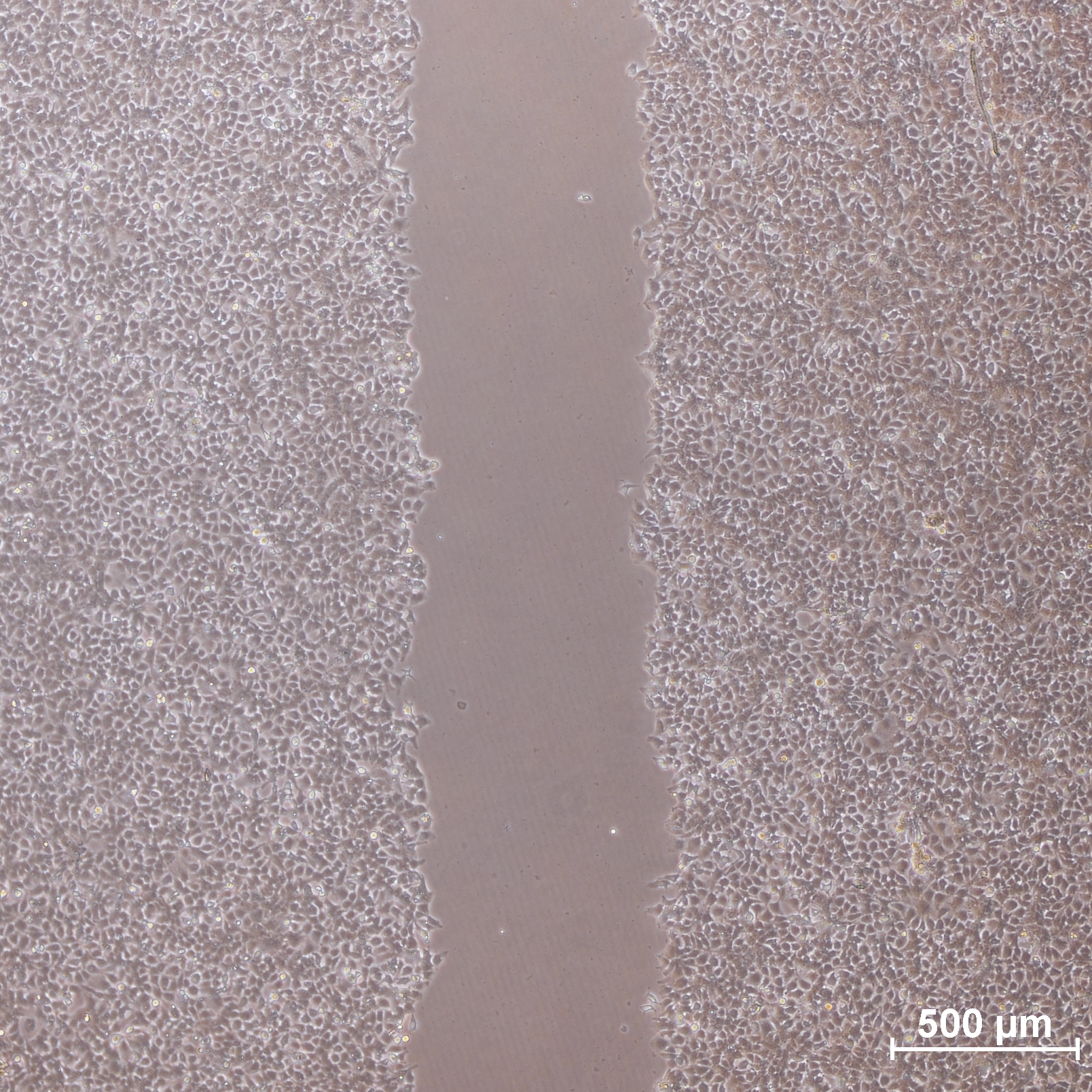

Supplement: Supplementary file 6 [file DataSheet4.zip › scratch width 12h/0-1/3.jpg]

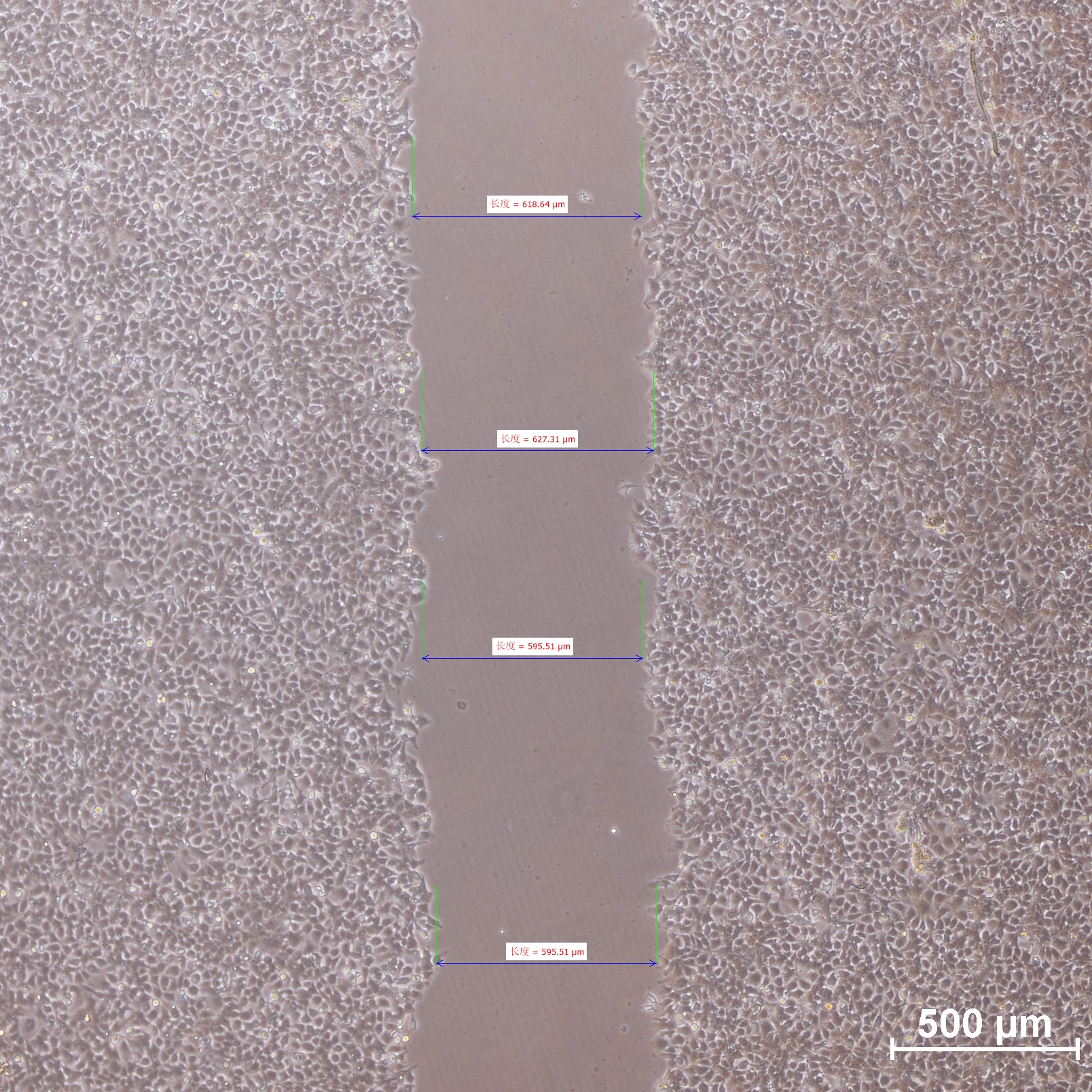

Supplement: Supplementary file 6 [file DataSheet4.zip › scratch width 12h/0-1/4.jpg]

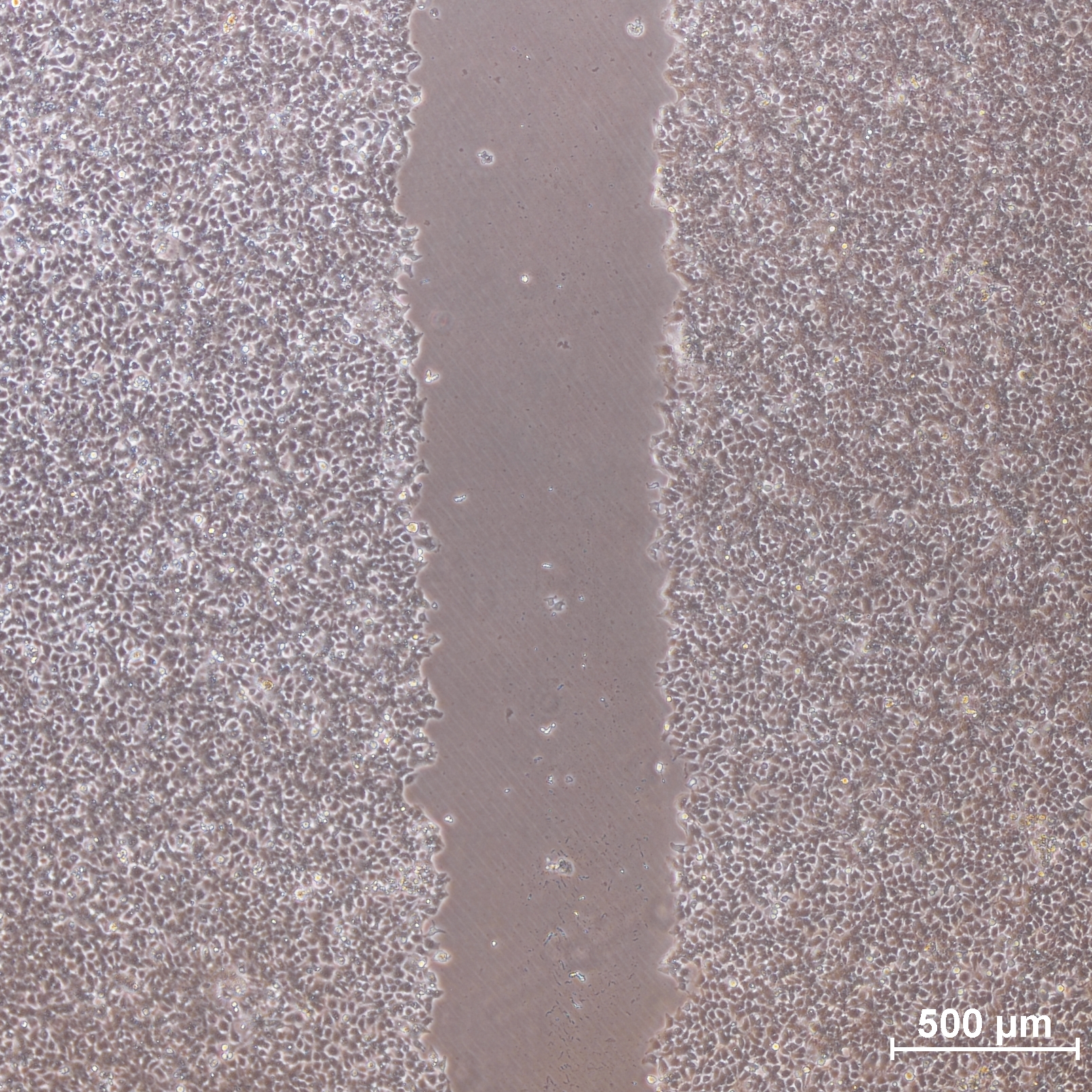

Supplement: Supplementary file 6 [file DataSheet4.zip › scratch width 12h/0-1/5.jpg]

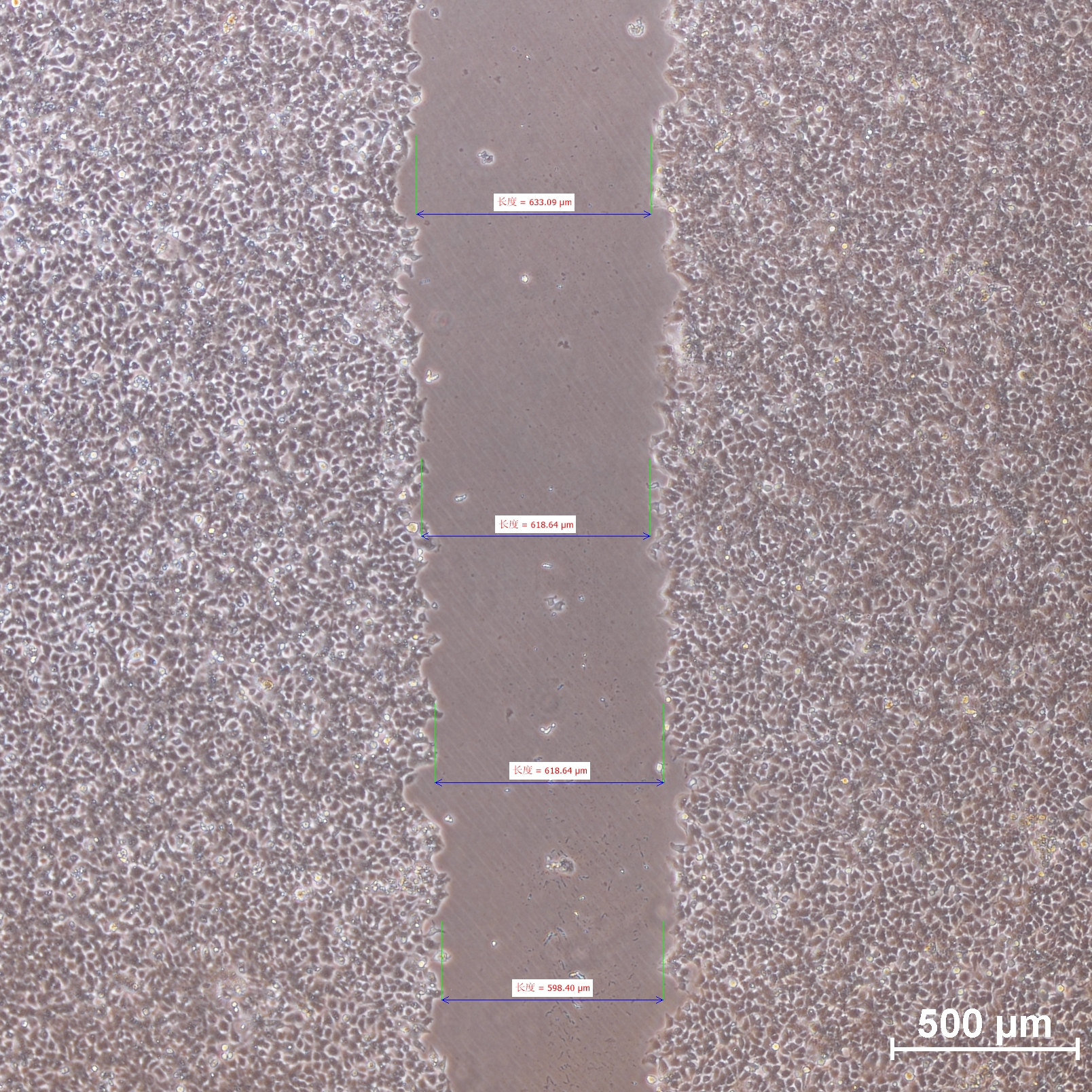

Supplement: Supplementary file 6 [file DataSheet4.zip › scratch width 12h/0-1/6.jpg]

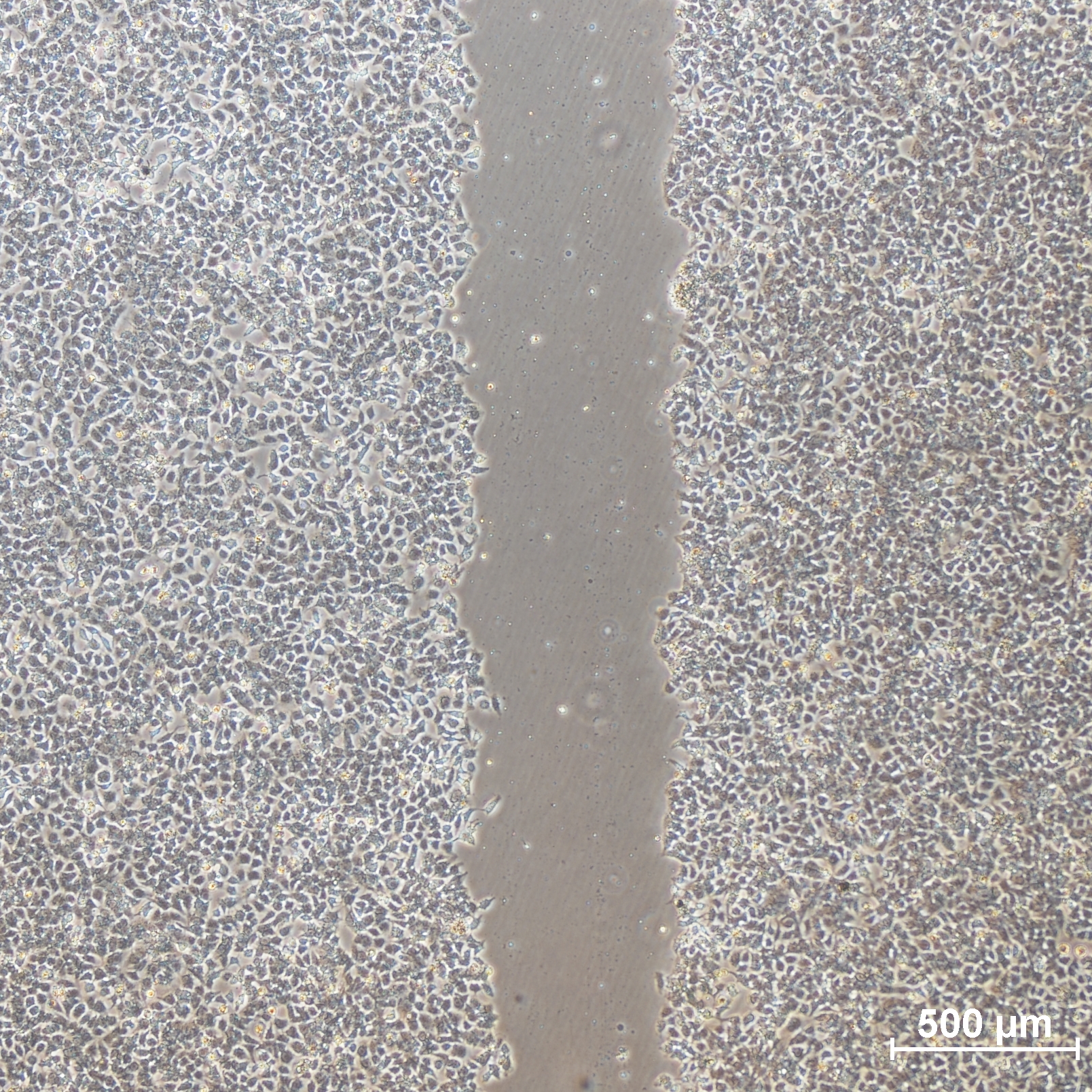

Supplement: Supplementary file 6 [file DataSheet4.zip › scratch width 12h/0.25-1/1.jpg]

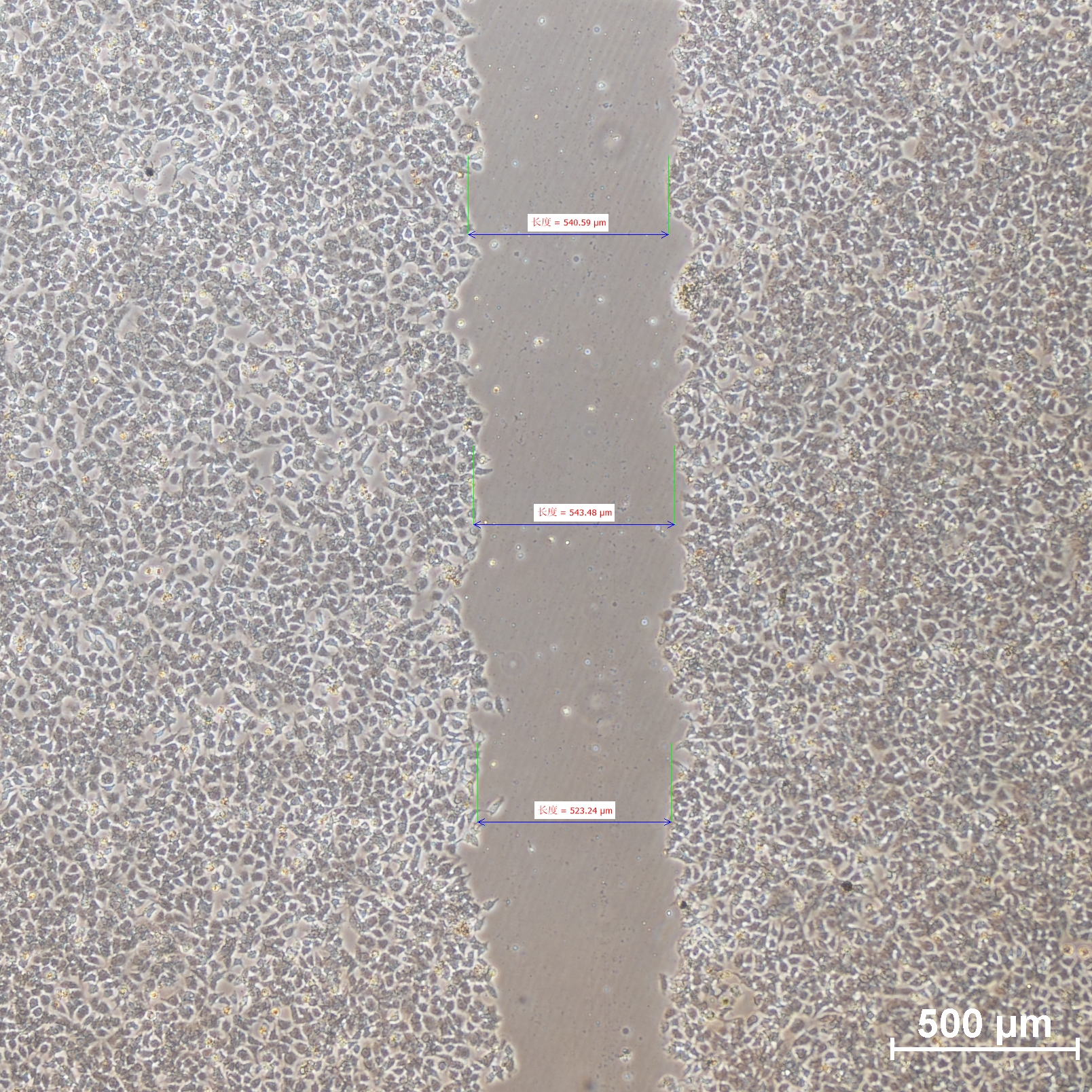

Supplement: Supplementary file 6 [file DataSheet4.zip › scratch width 12h/0.25-1/2.jpg]

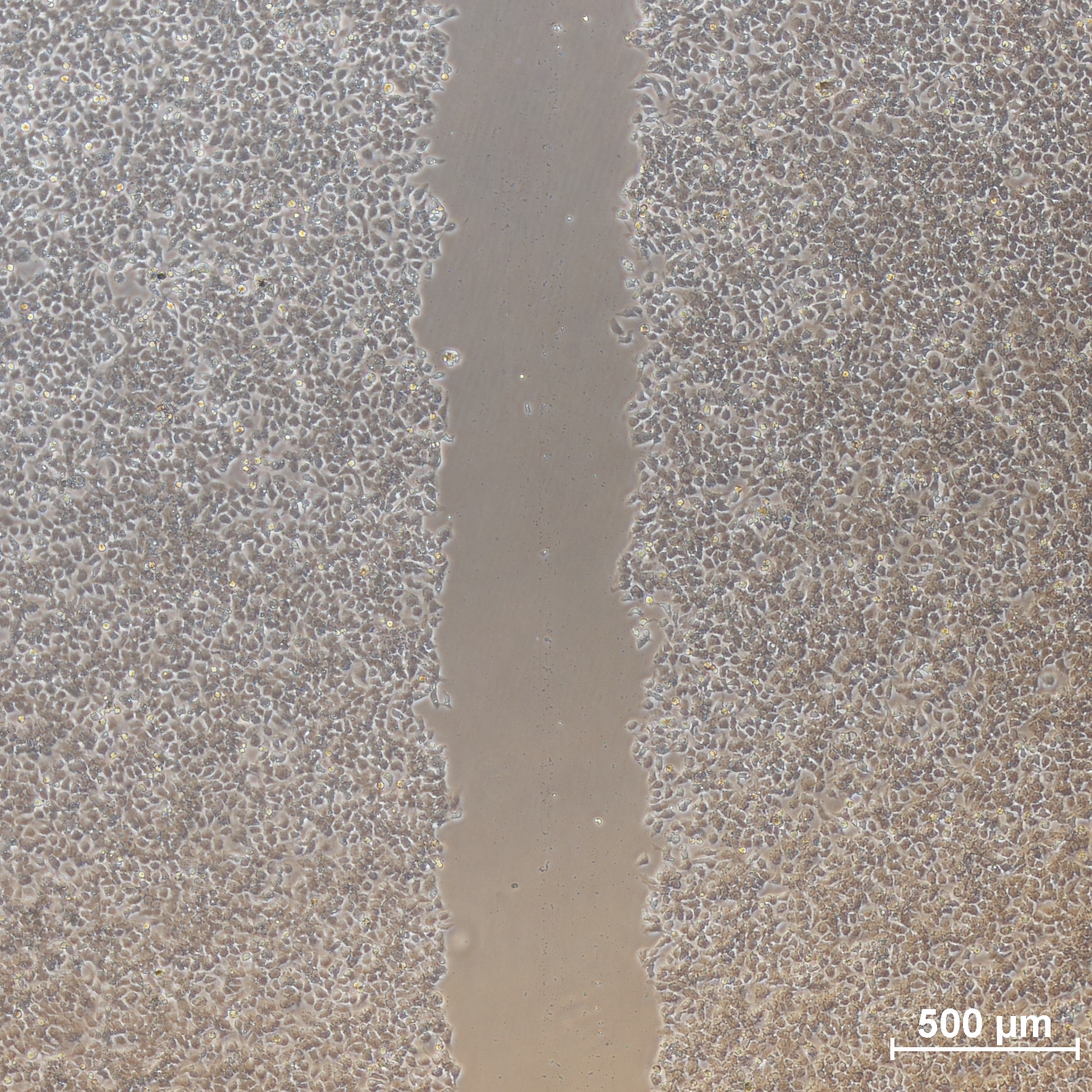

Supplement: Supplementary file 6 [file DataSheet4.zip › scratch width 12h/0.25-1/3.jpg]

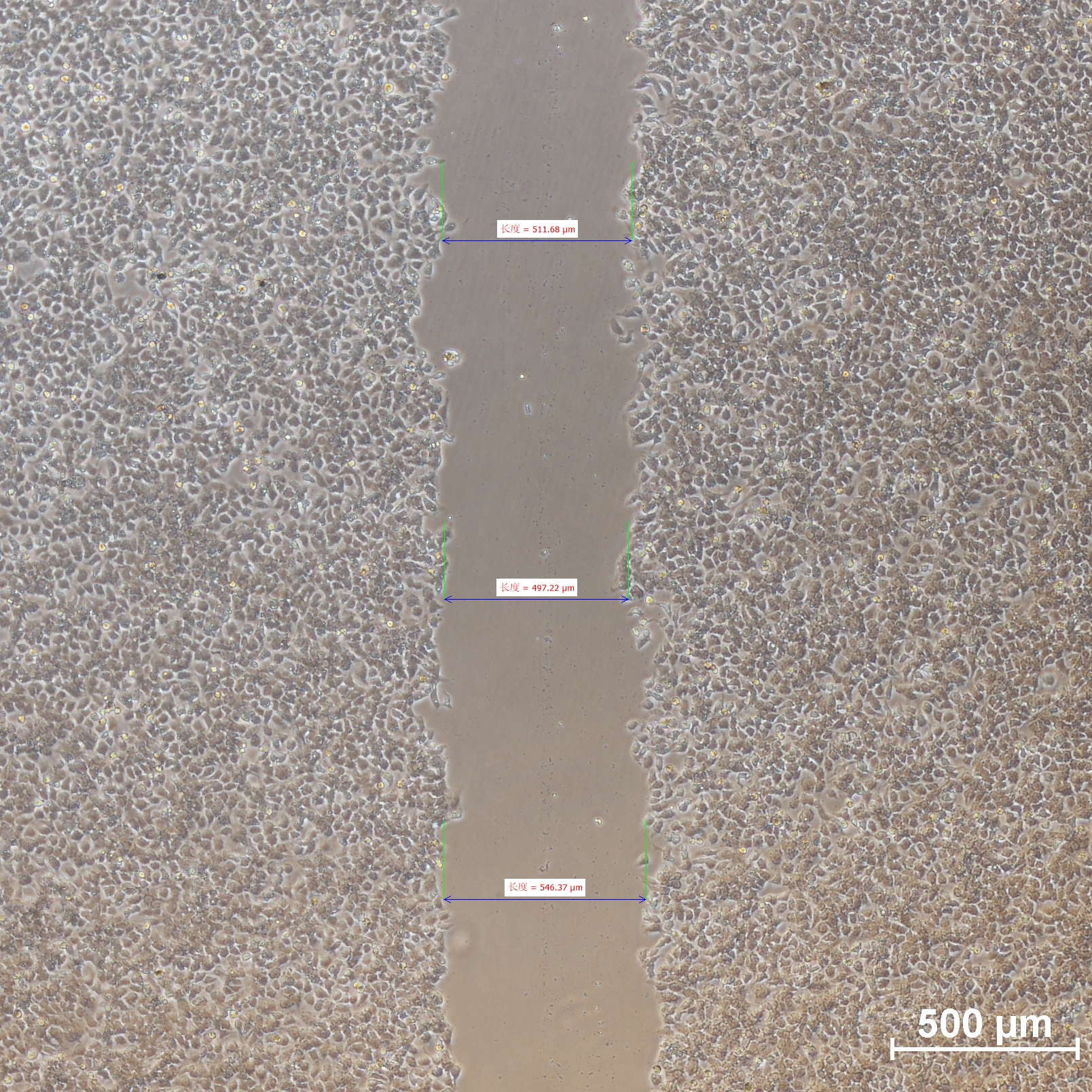

Supplement: Supplementary file 6 [file DataSheet4.zip › scratch width 12h/0.25-1/4.jpg]

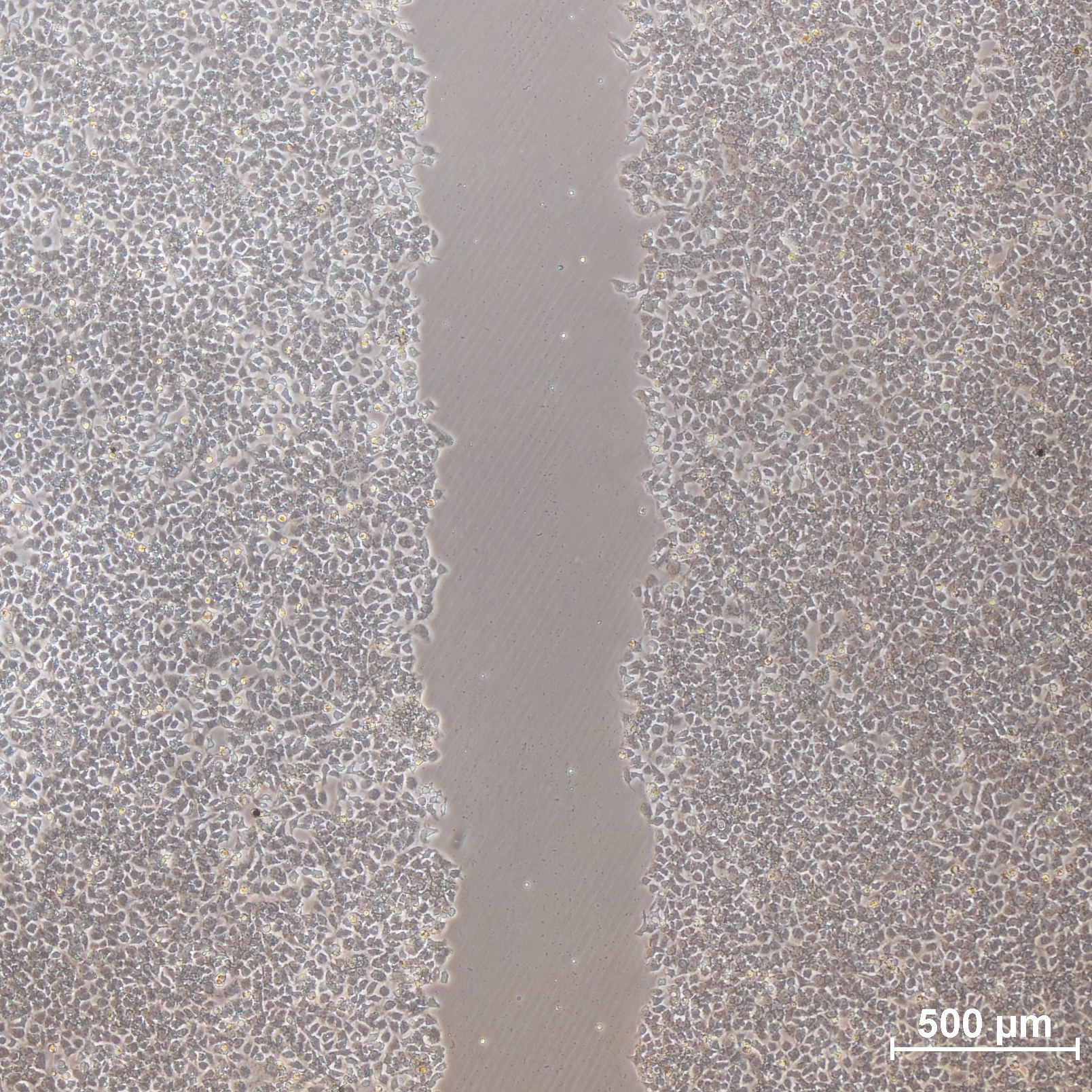

Supplement: Supplementary file 6 [file DataSheet4.zip › scratch width 12h/0.25-1/5.jpg]

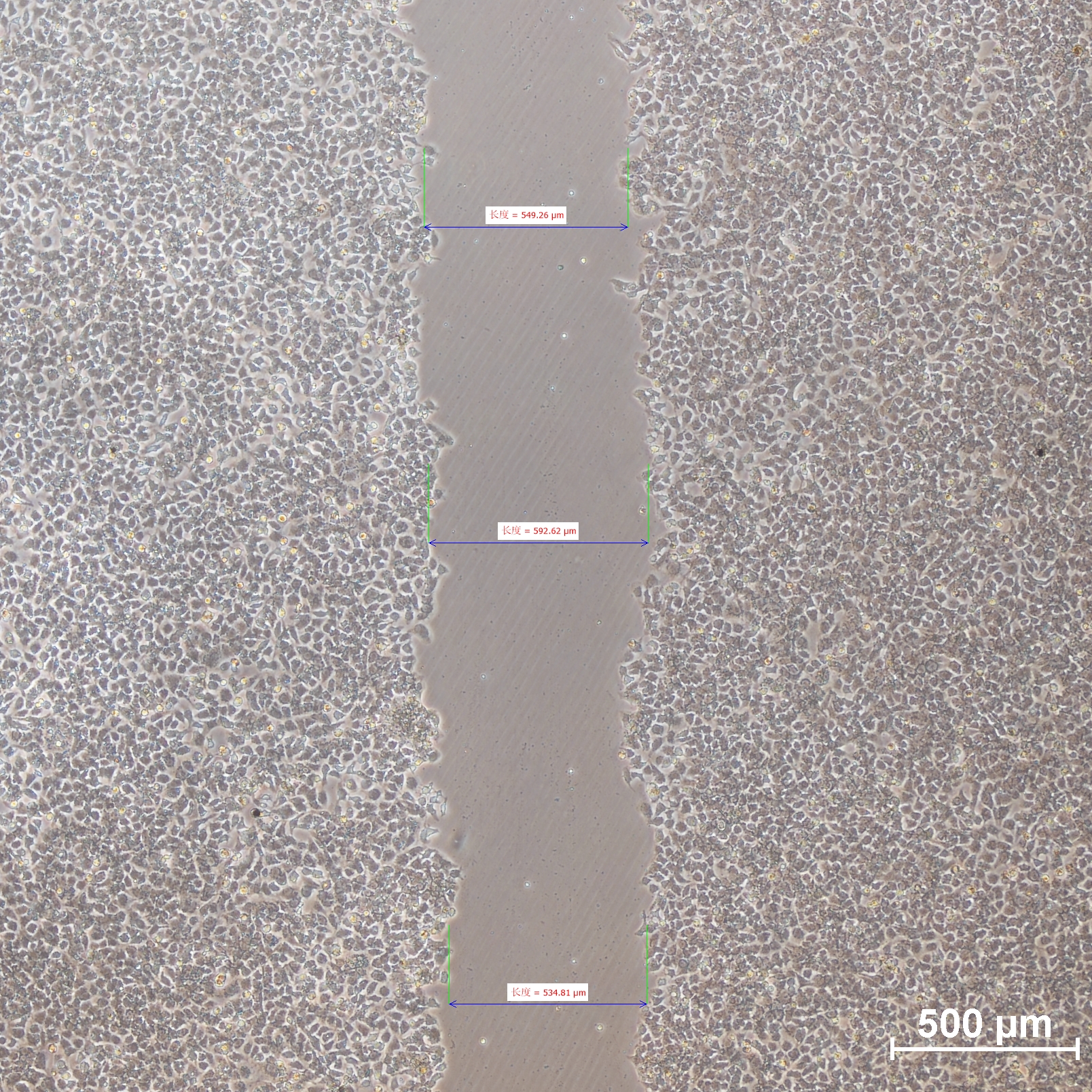

Supplement: Supplementary file 6 [file DataSheet4.zip › scratch width 12h/0.25-1/6.jpg]

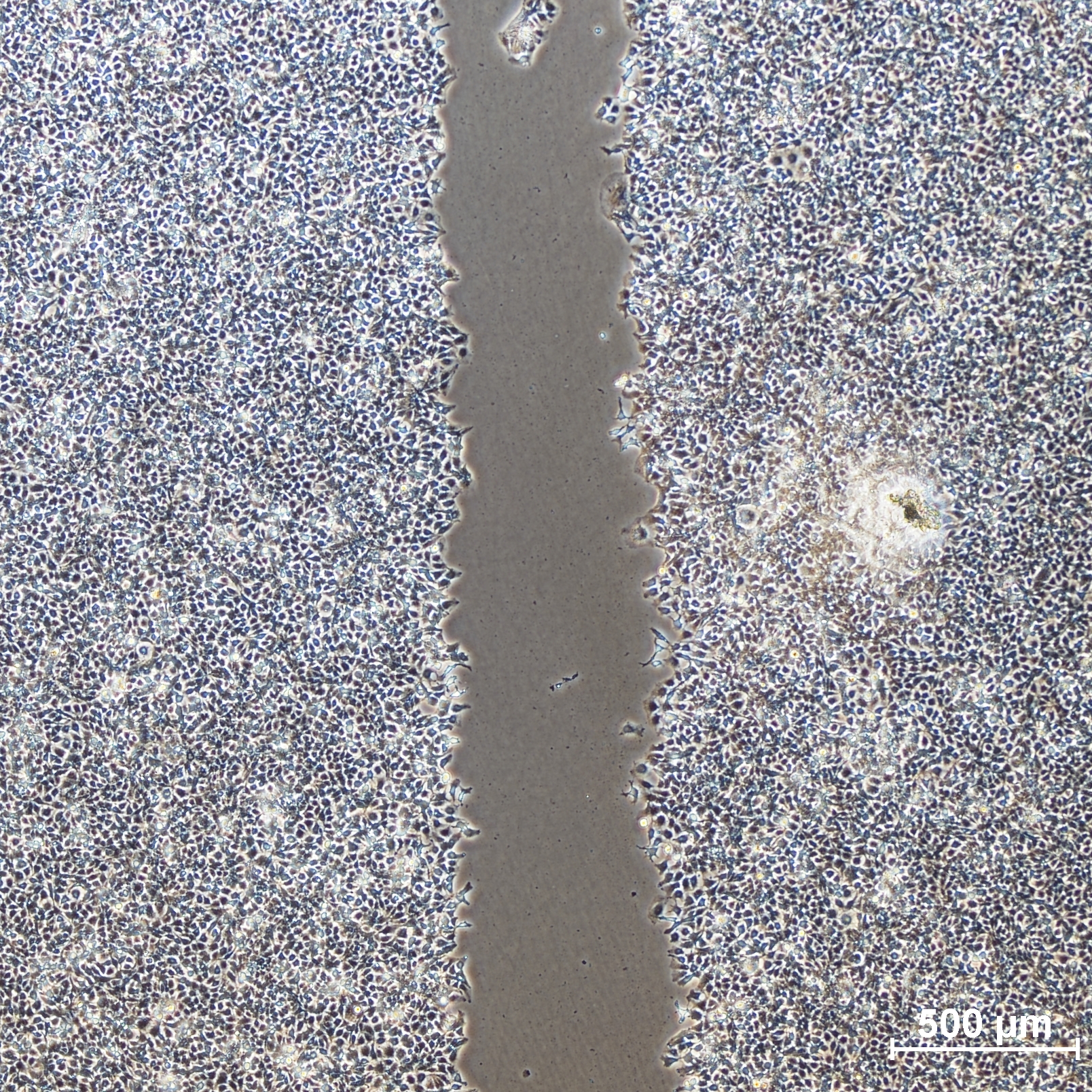

Supplement: Supplementary file 7 [file DataSheet5.zip › 0mMscratch width 24h/1.jpg]

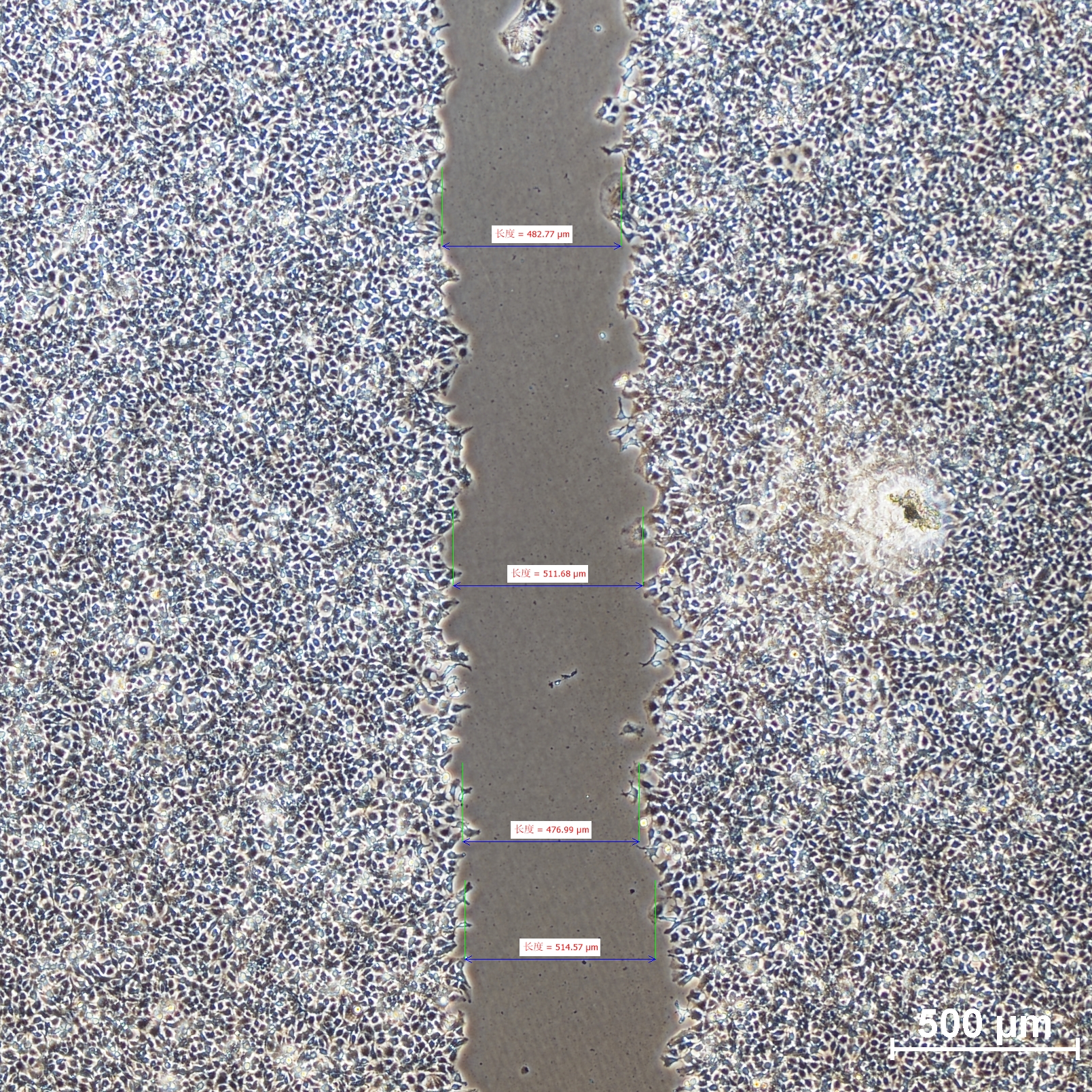

Supplement: Supplementary file 7 [file DataSheet5.zip › 0mMscratch width 24h/2.jpg]

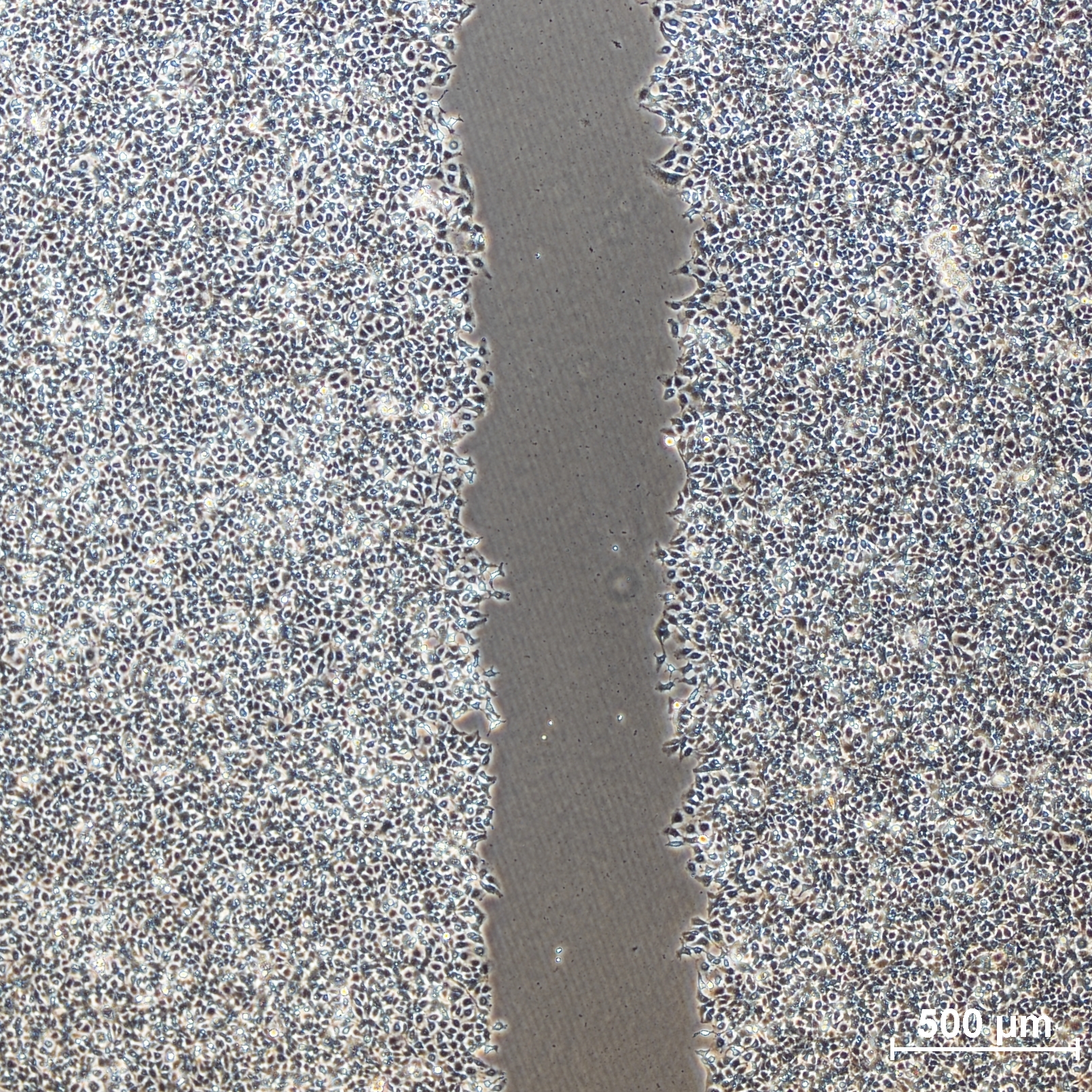

Supplement: Supplementary file 7 [file DataSheet5.zip › 0mMscratch width 24h/3.jpg]

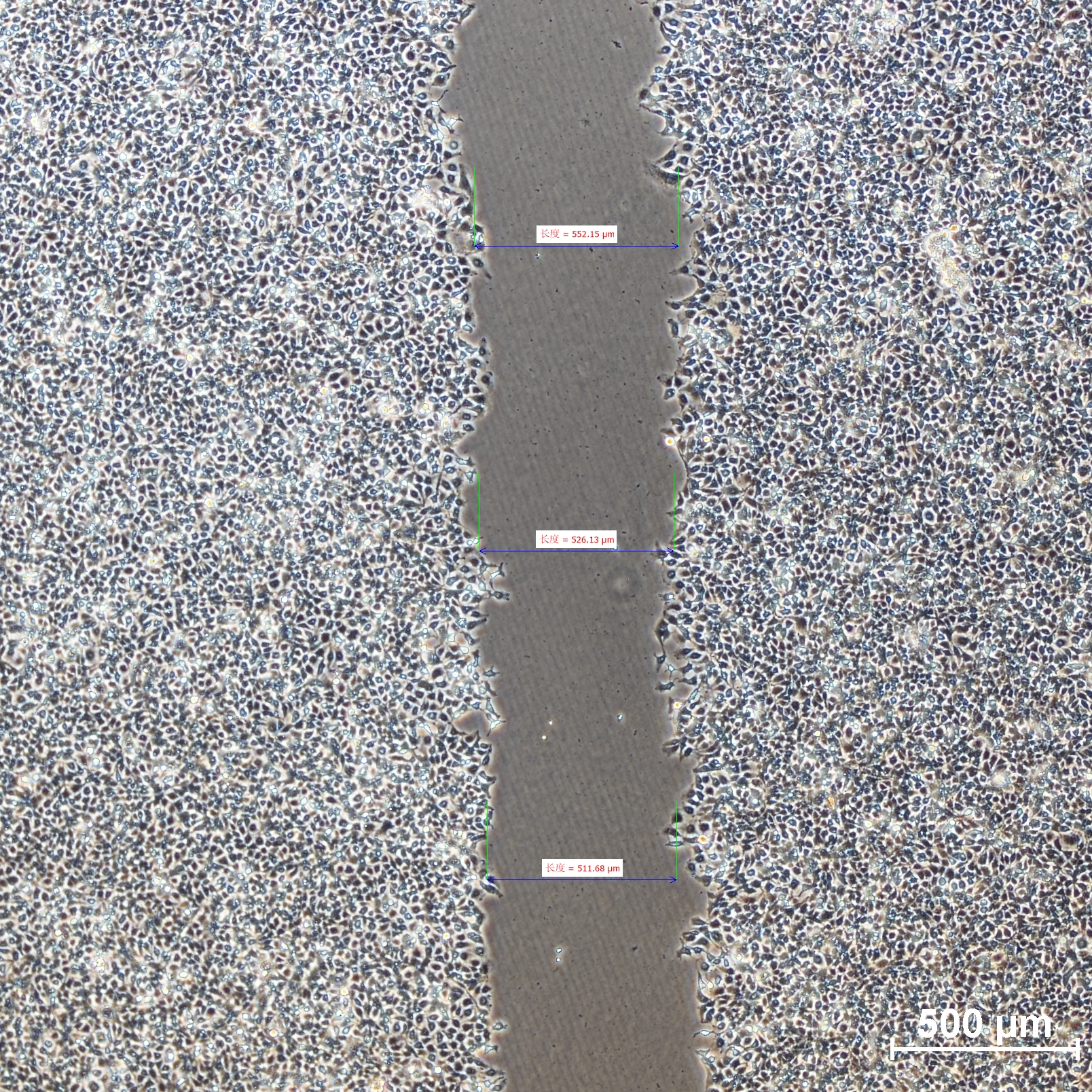

Supplement: Supplementary file 7 [file DataSheet5.zip › 0mMscratch width 24h/4.jpg]

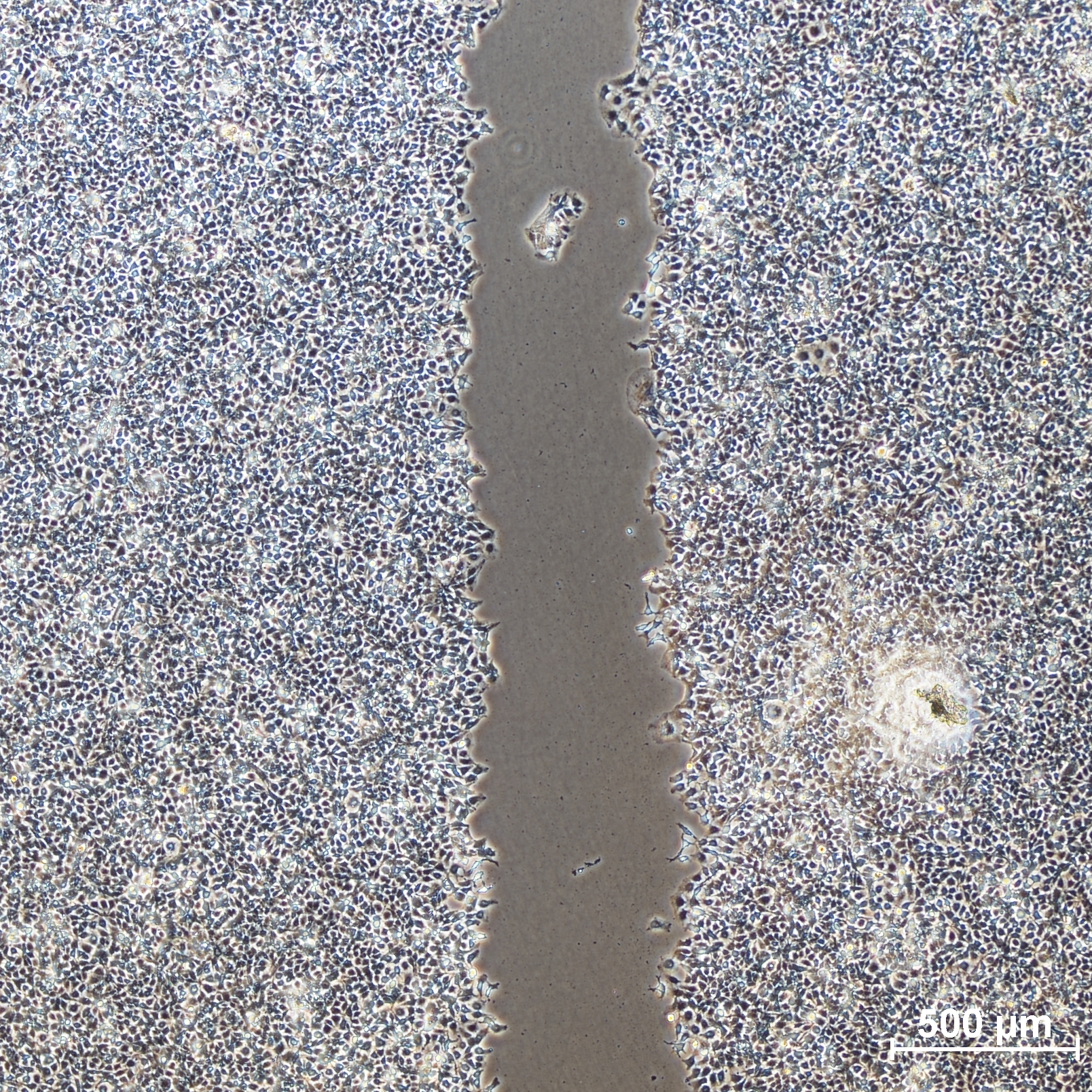

Supplement: Supplementary file 7 [file DataSheet5.zip › 0mMscratch width 24h/5.jpg]

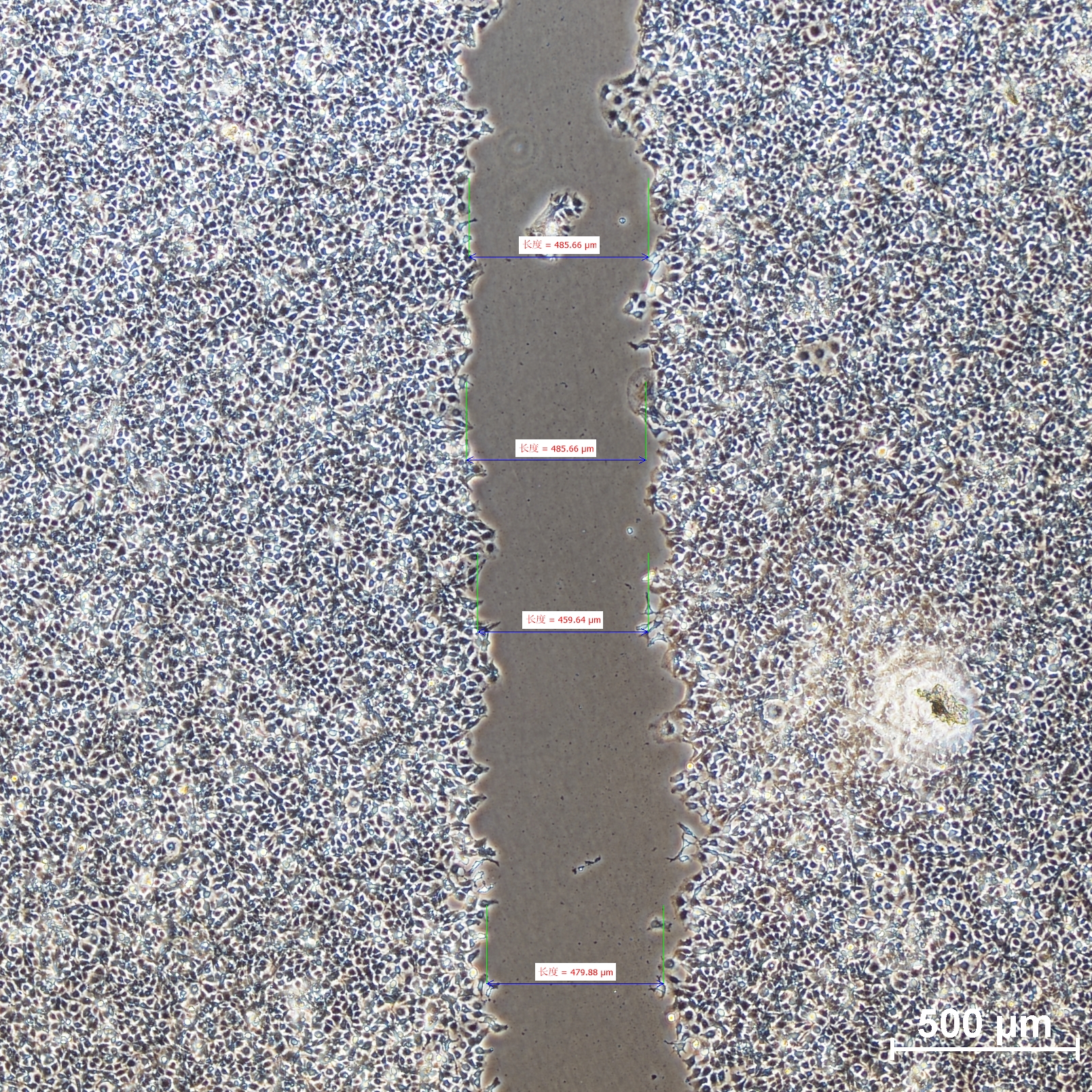

Supplement: Supplementary file 7 [file DataSheet5.zip › 0mMscratch width 24h/6.jpg]

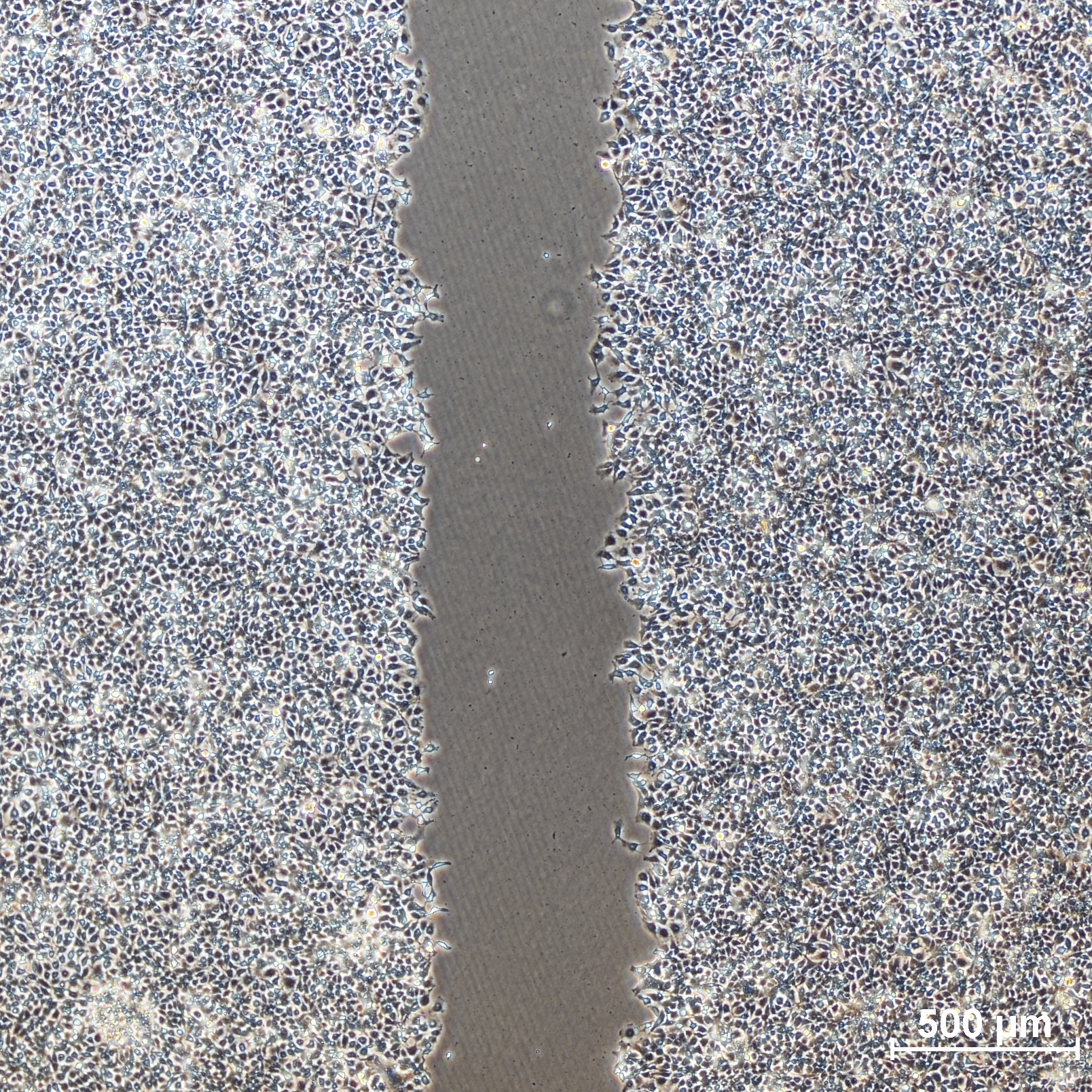

Supplement: Supplementary file 7 [file DataSheet5.zip › 0mMscratch width 24h/7.jpg]

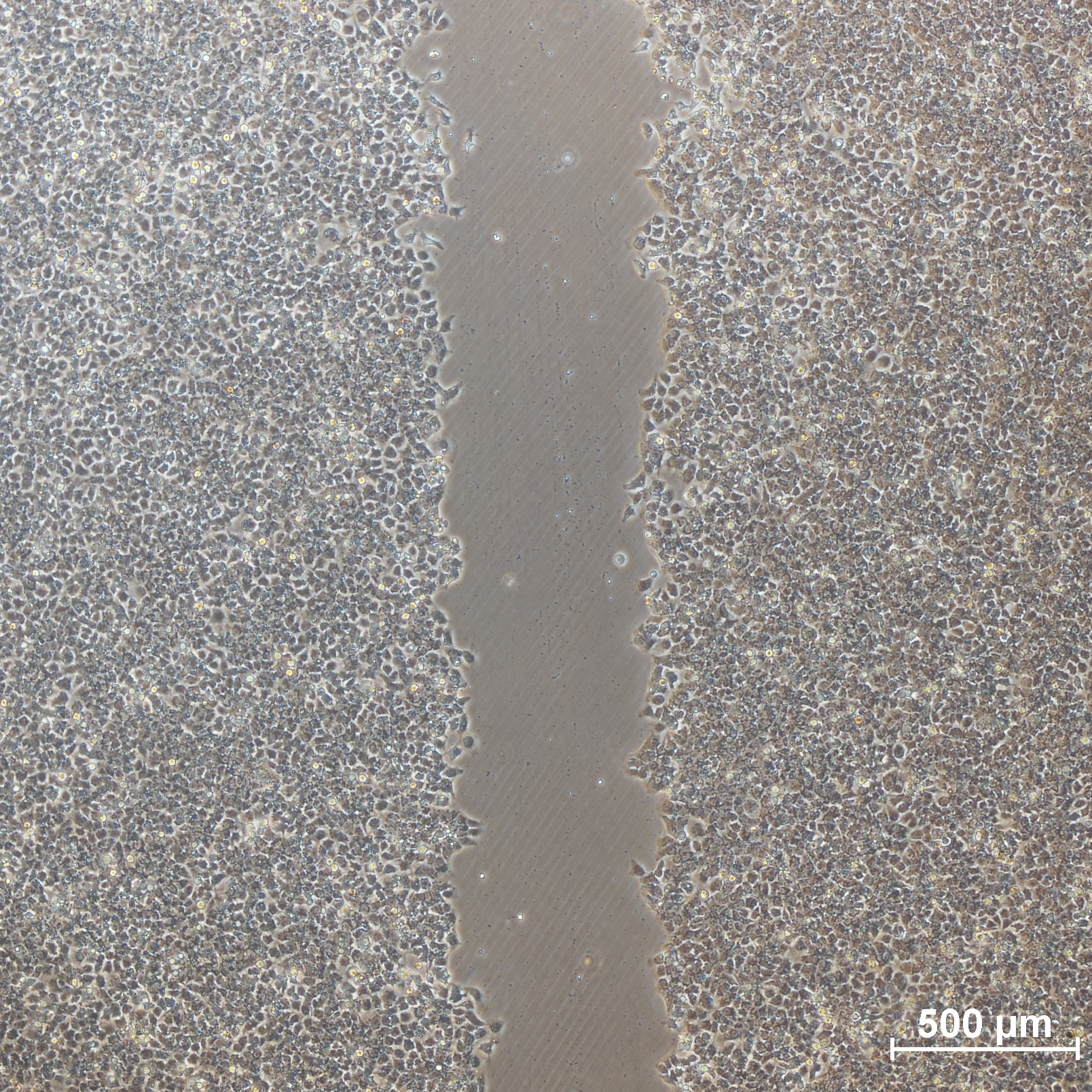

Supplement: Supplementary file 8 [file DataSheet6.zip › 0.25mMscratch width 24h/1.jpg]

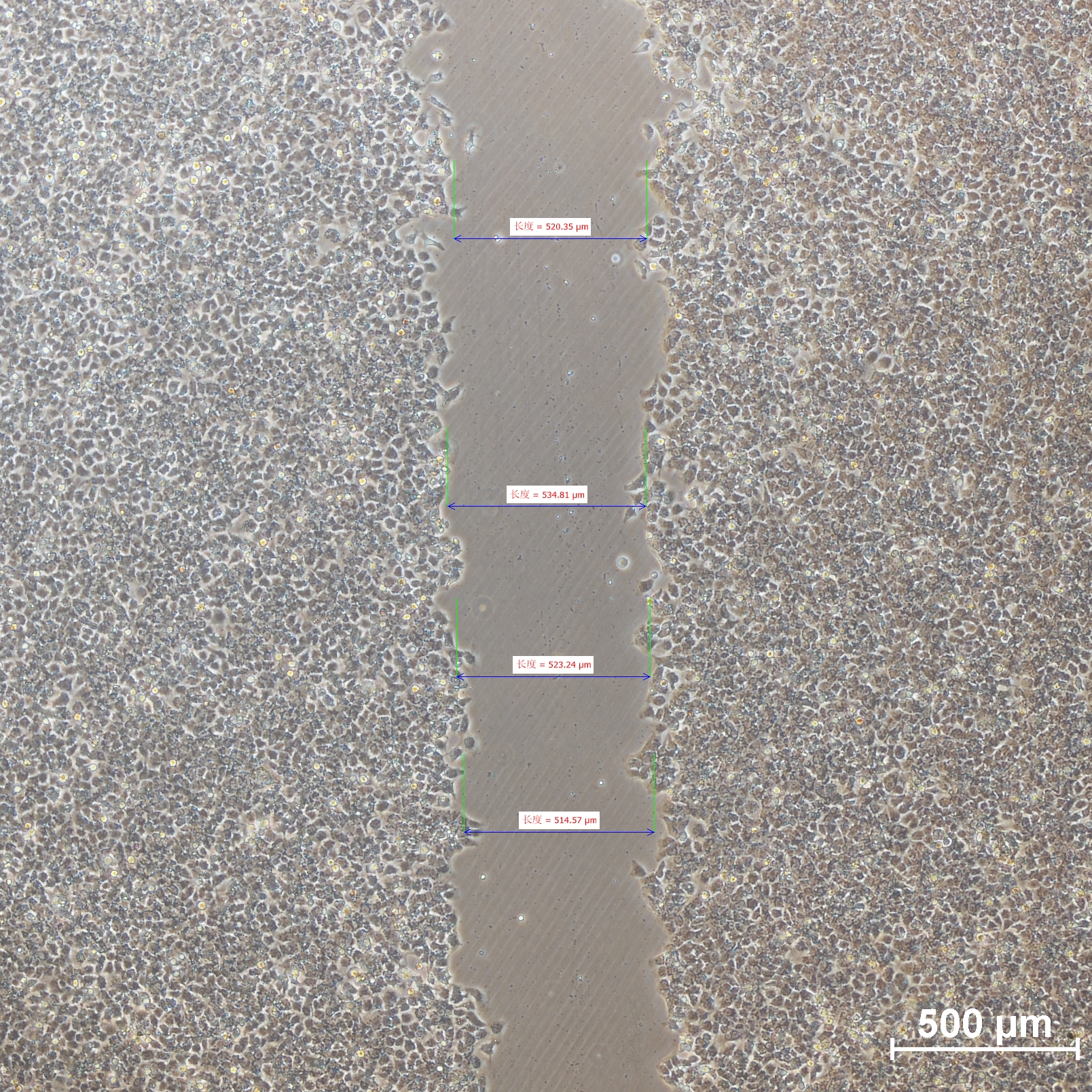

Supplement: Supplementary file 8 [file DataSheet6.zip › 0.25mMscratch width 24h/2.jpg]

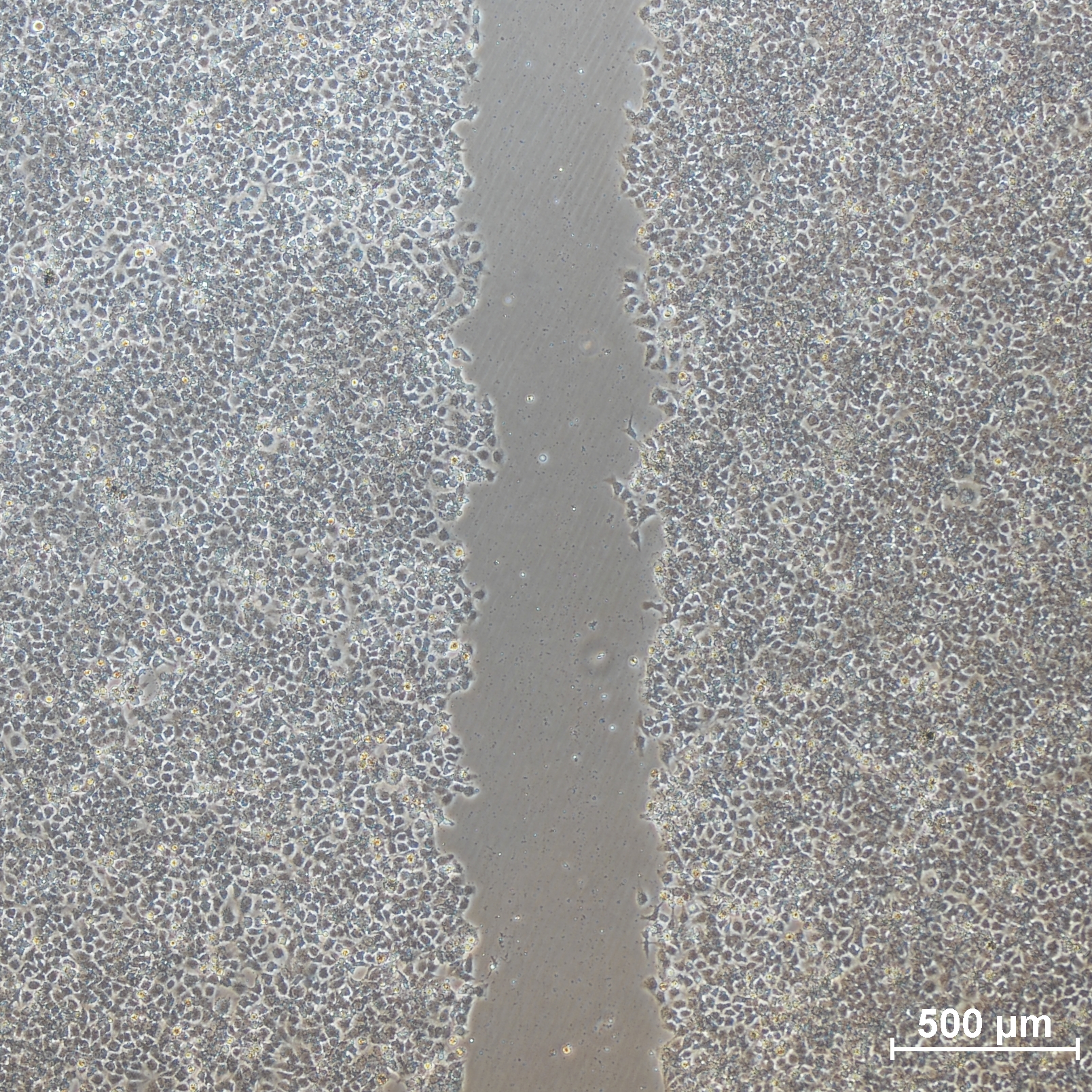

Supplement: Supplementary file 8 [file DataSheet6.zip › 0.25mMscratch width 24h/3.jpg]

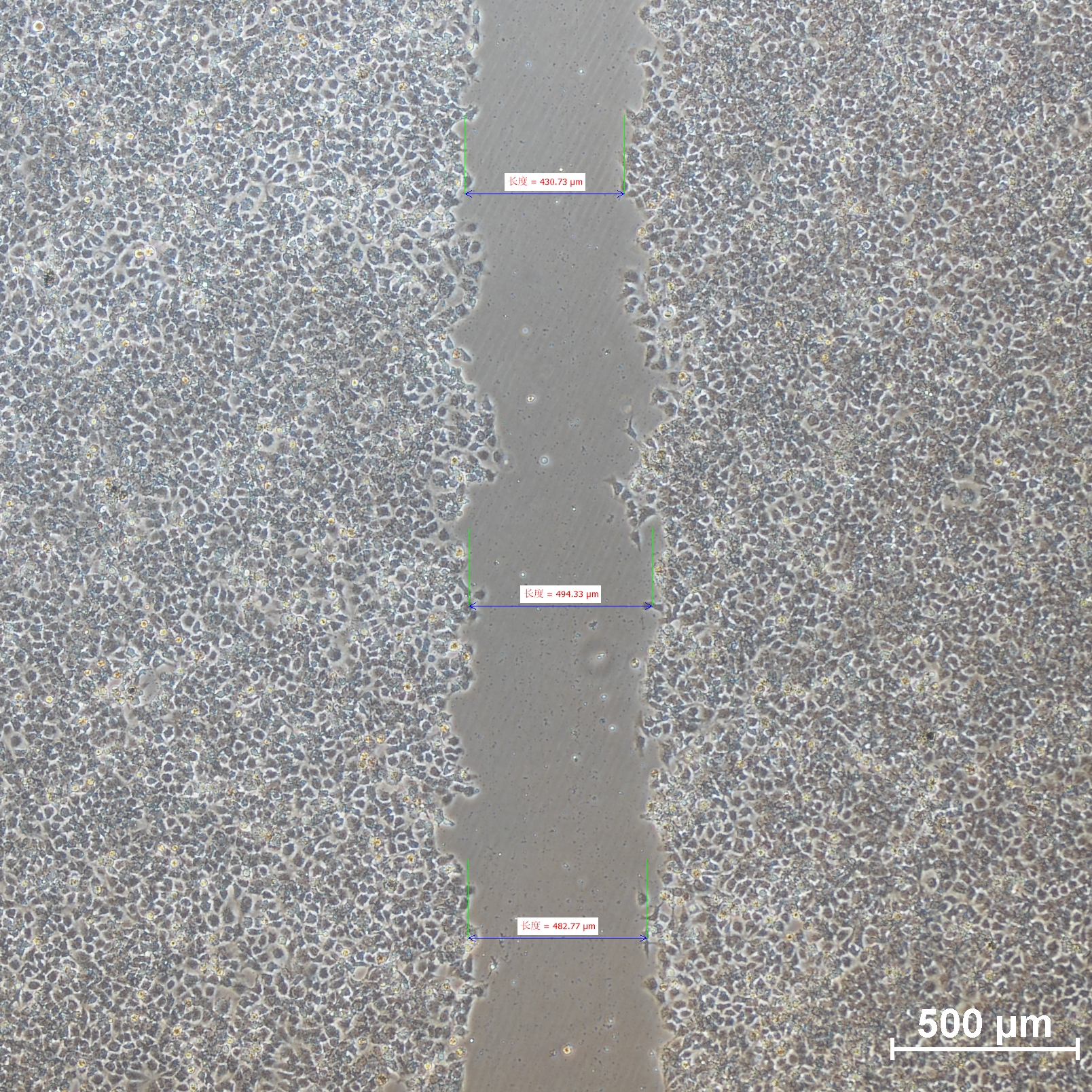

Supplement: Supplementary file 8 [file DataSheet6.zip › 0.25mMscratch width 24h/4.jpg]

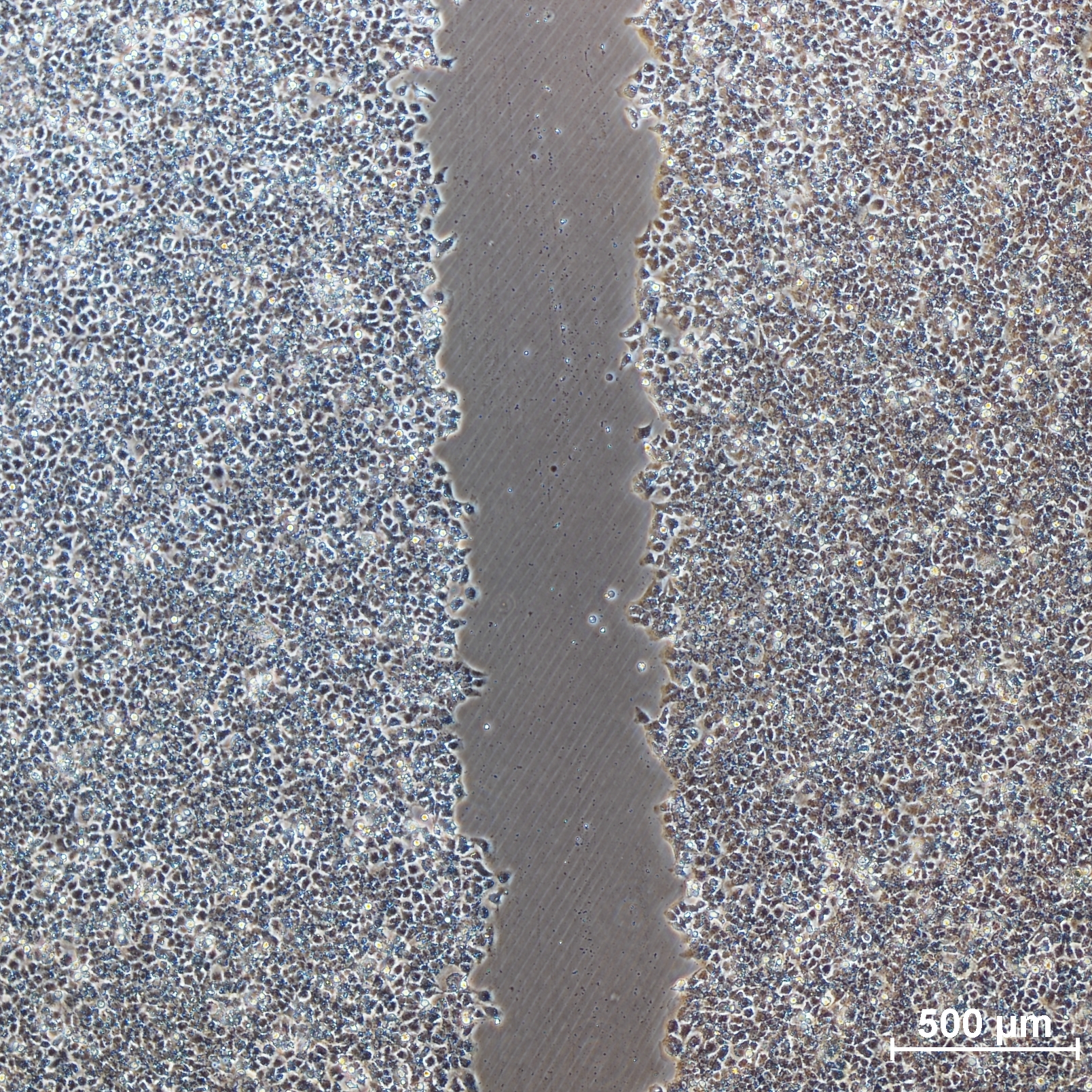

Supplement: Supplementary file 8 [file DataSheet6.zip › 0.25mMscratch width 24h/5.jpg]

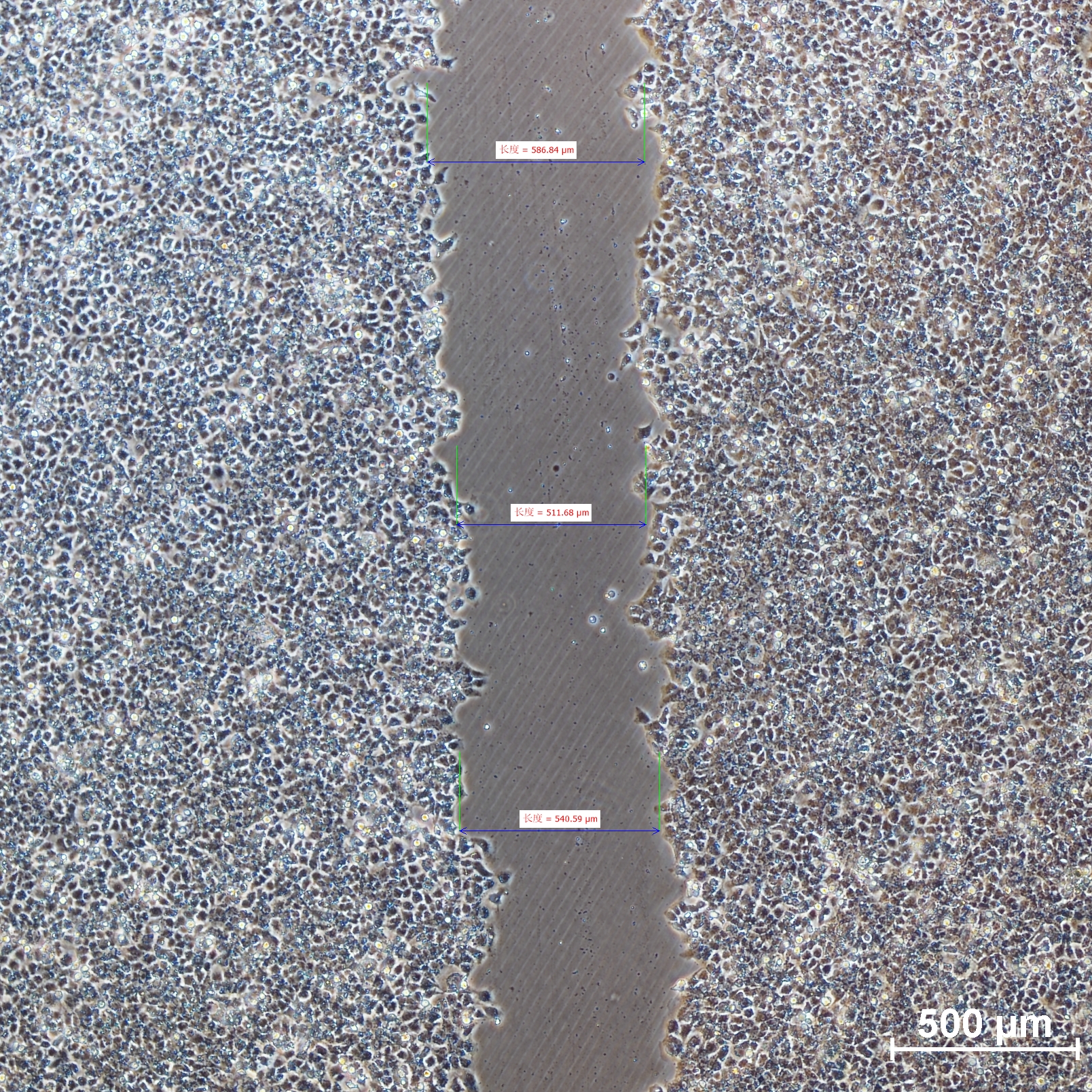

Supplement: Supplementary file 8 [file DataSheet6.zip › 0.25mMscratch width 24h/6.jpg]

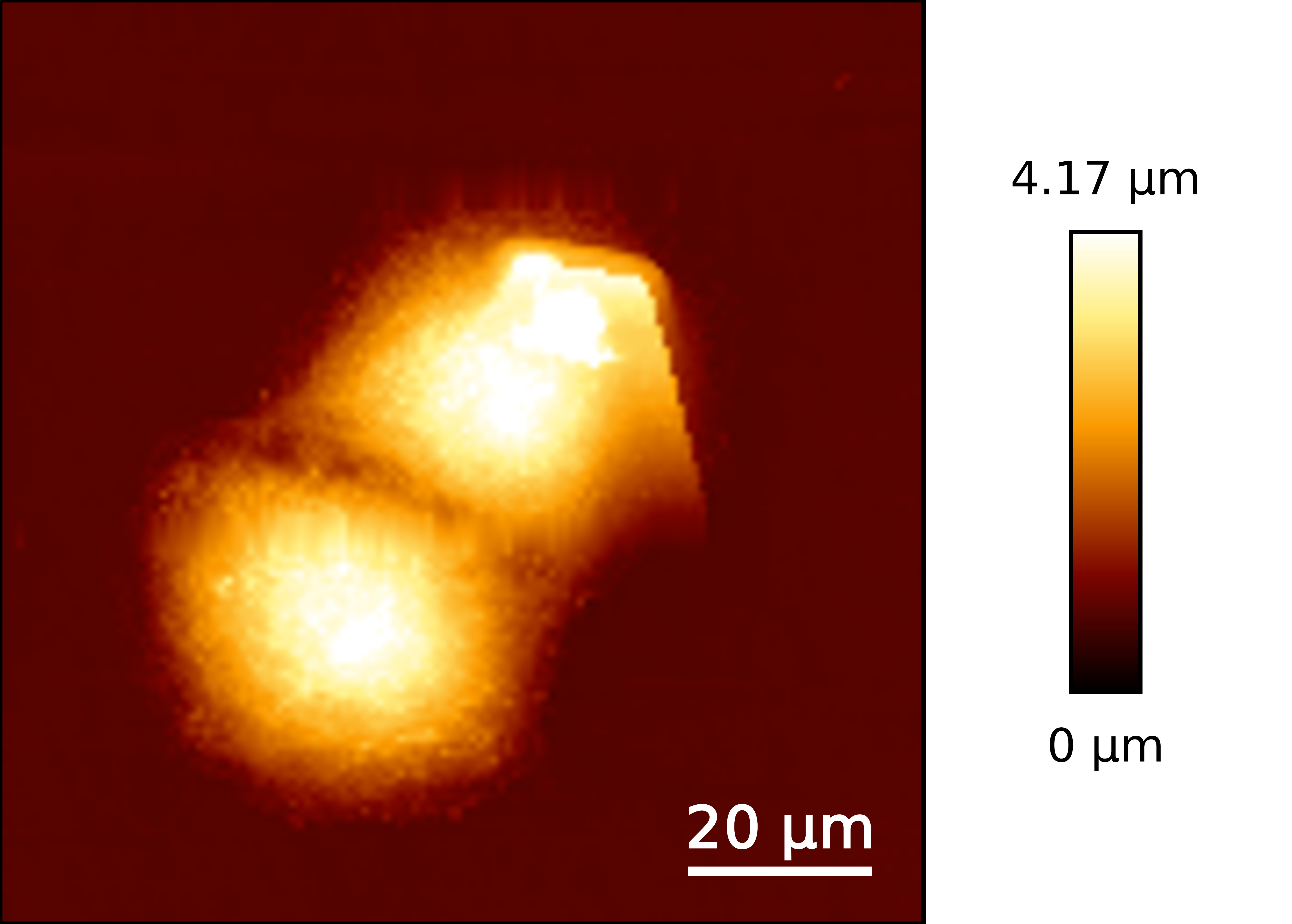

Supplement: Supplementary file 9 [file DataSheet7.zip › AFM/qi-fit-2025.07.03-10.59.10.958_reference-force-height-default-1.png]

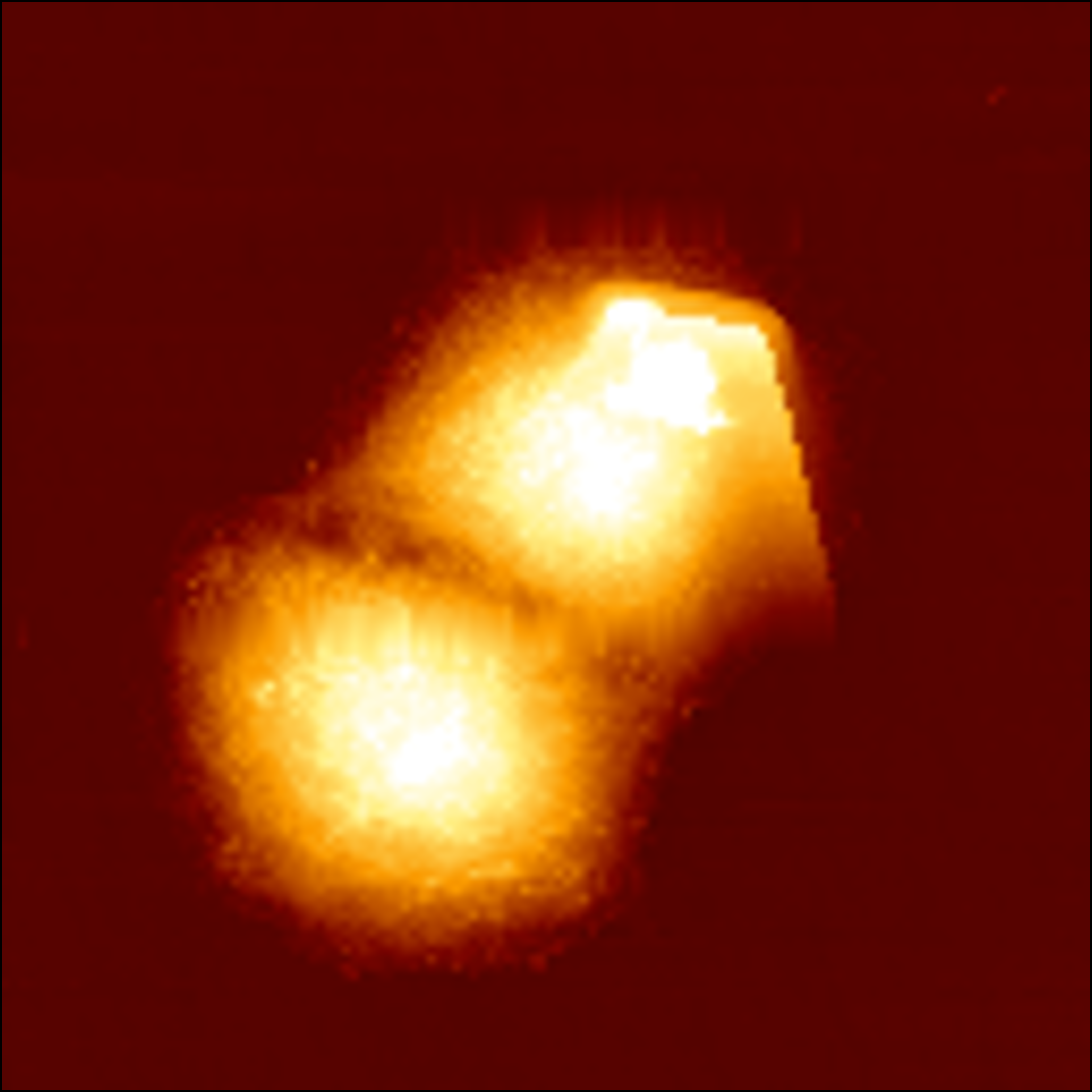

Supplement: Supplementary file 9 [file DataSheet7.zip › AFM/qi-fit-2025.07.03-10.59.10.958_reference-force-height-default.png]

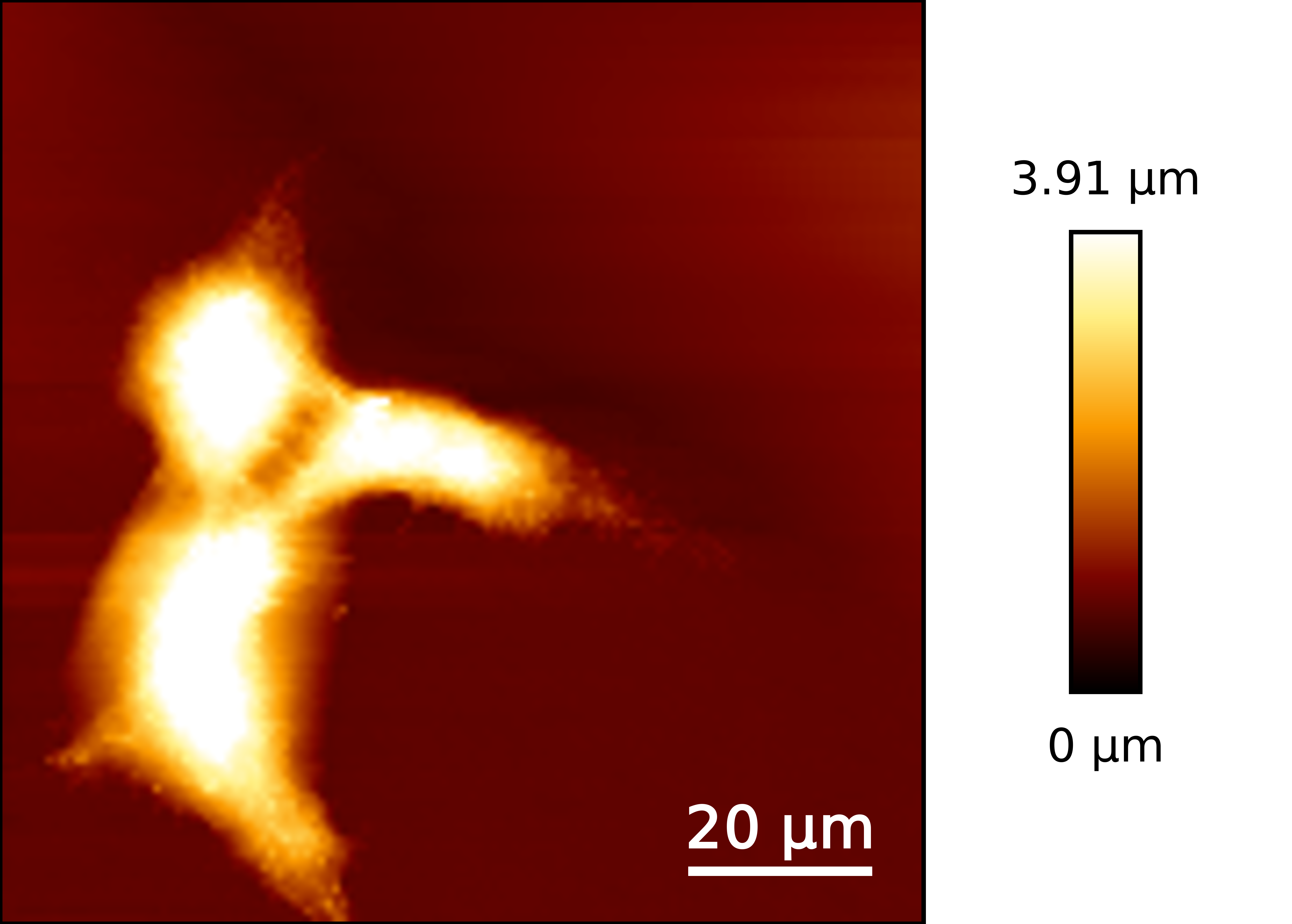

Supplement: Supplementary file 9 [file DataSheet7.zip › AFM/qi-fit-2025.07.03-14.42.59.719_reference-force-height-default-1.png]

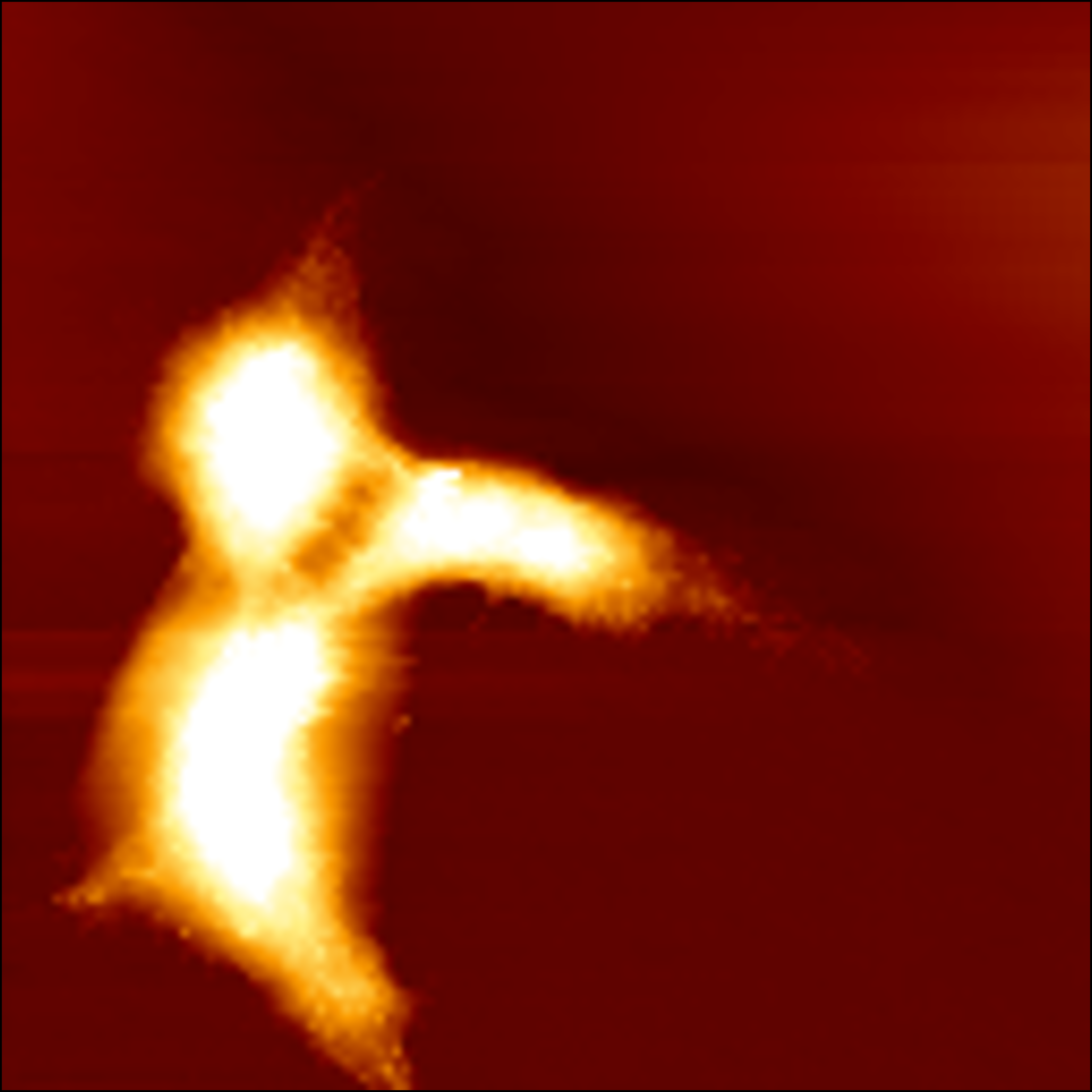

Supplement: Supplementary file 9 [file DataSheet7.zip › AFM/qi-fit-2025.07.03-14.42.59.719_reference-force-height-default.png]

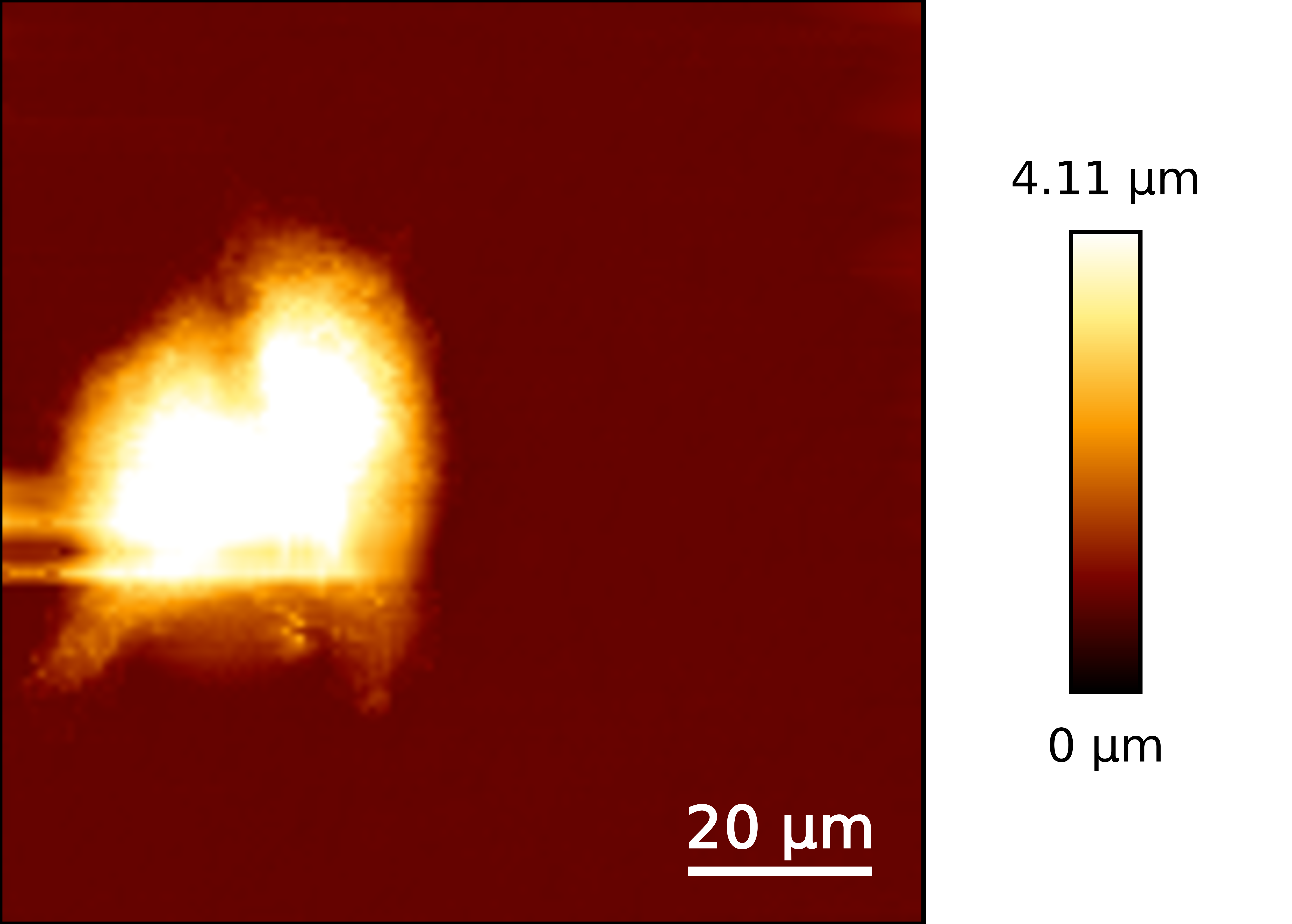

Supplement: Supplementary file 9 [file DataSheet7.zip › AFM/qi-fit-2025.07.03-16.22.40.826_reference-force-height-default-1.png]

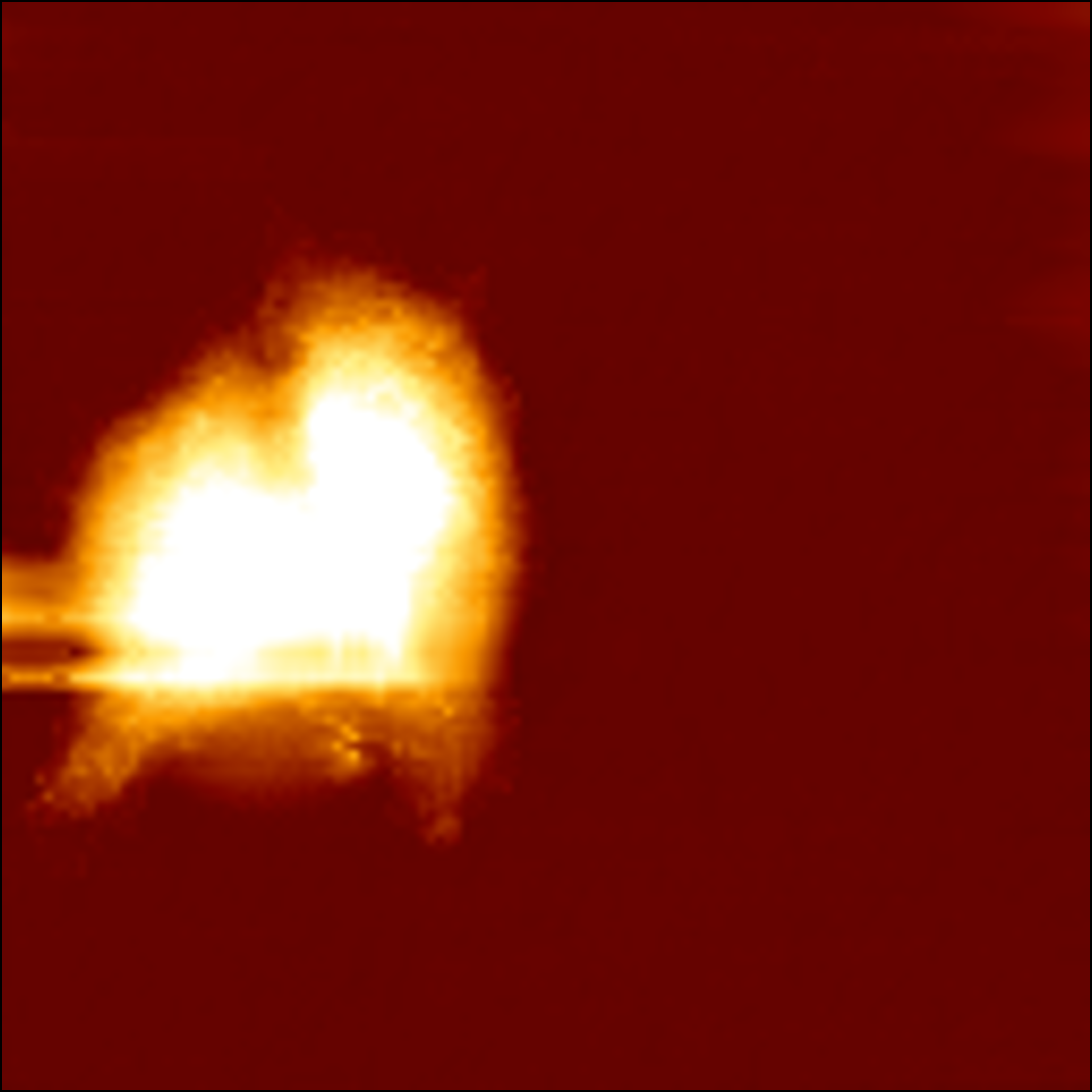

Supplement: Supplementary file 9 [file DataSheet7.zip › AFM/qi-fit-2025.07.03-16.22.40.826_reference-force-height-default.png]

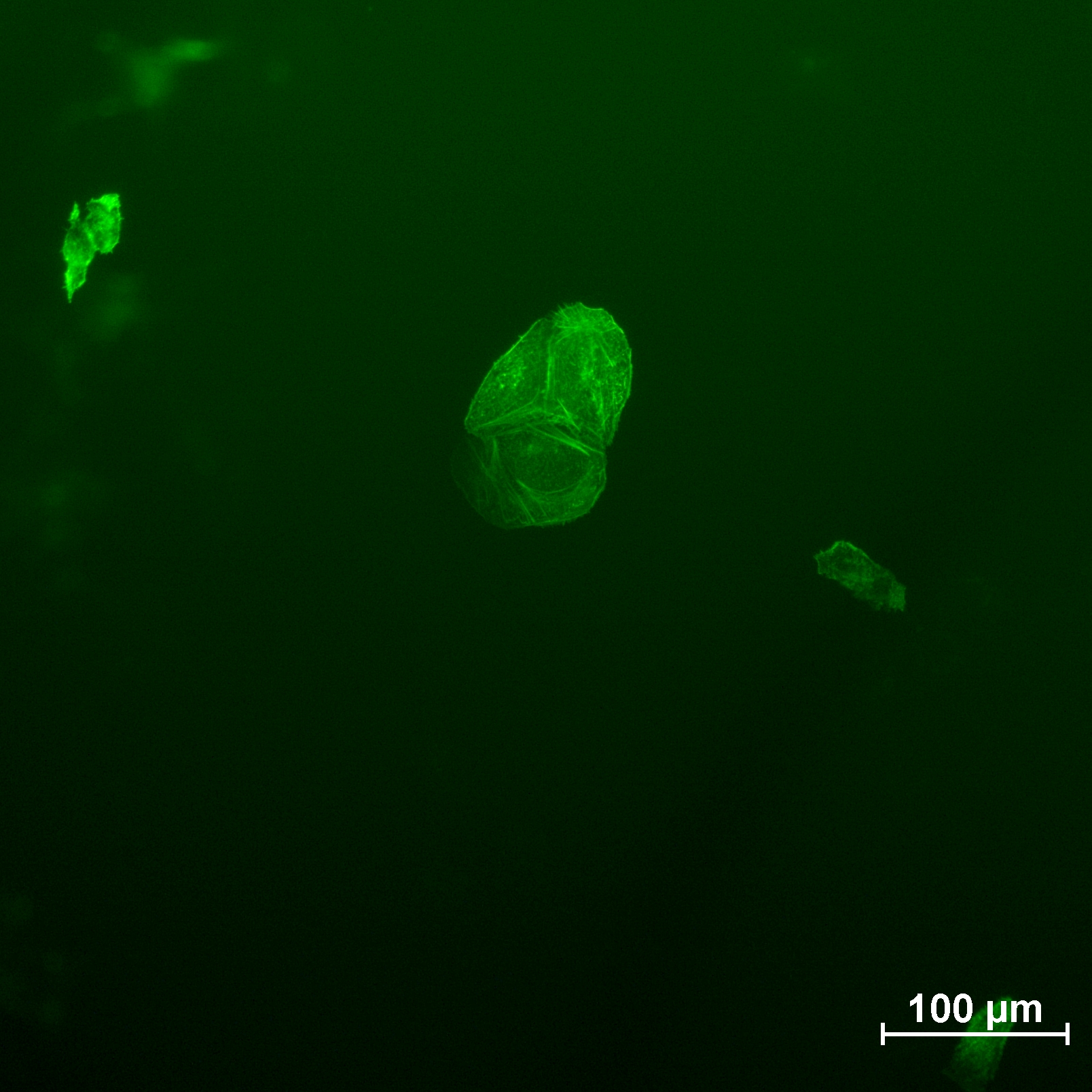

Supplement: Supplementary file 10 [file DataSheet8.zip › Cytoskeletal Damage/0/0.jpg]

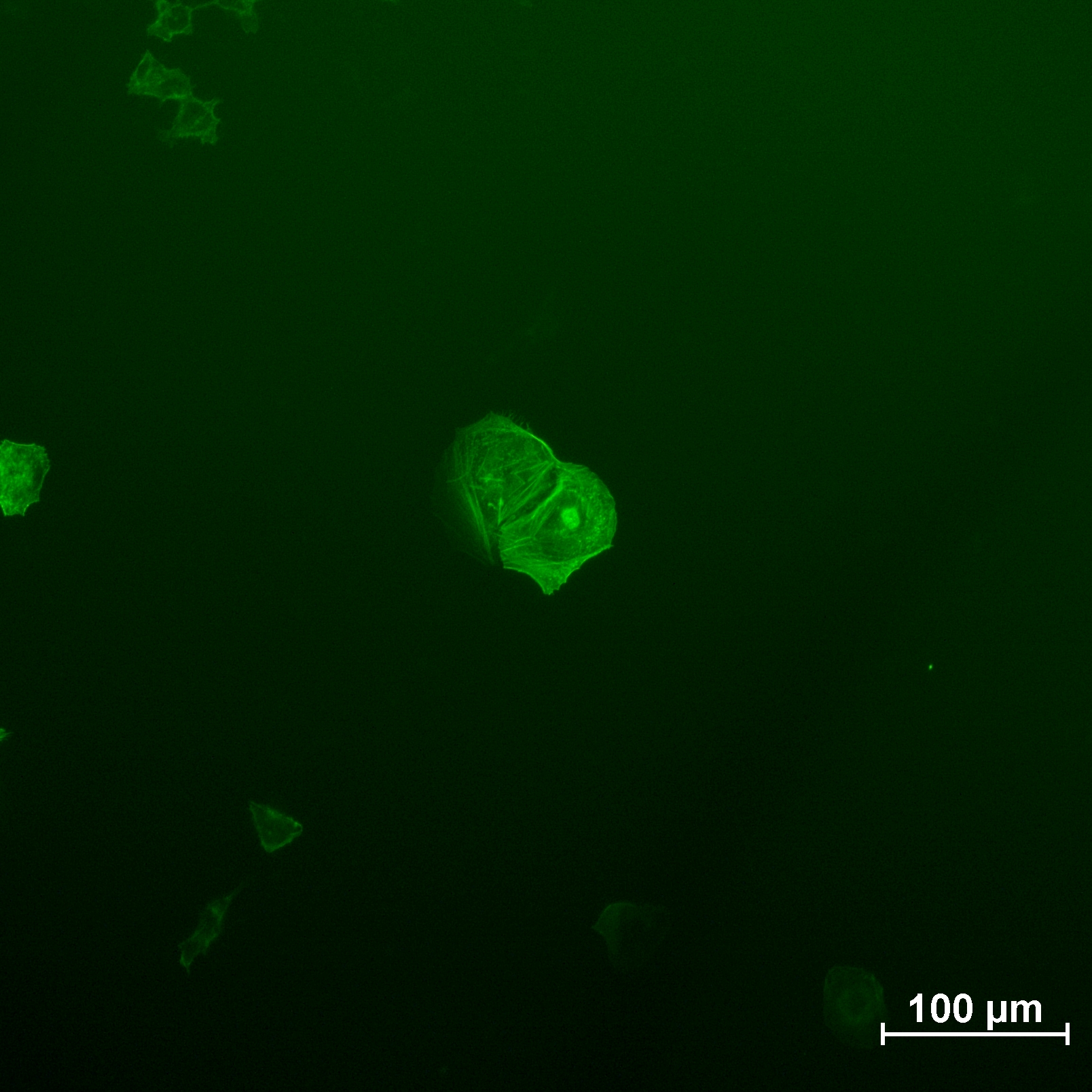

Supplement: Supplementary file 10 [file DataSheet8.zip › Cytoskeletal Damage/0/2.jpg]

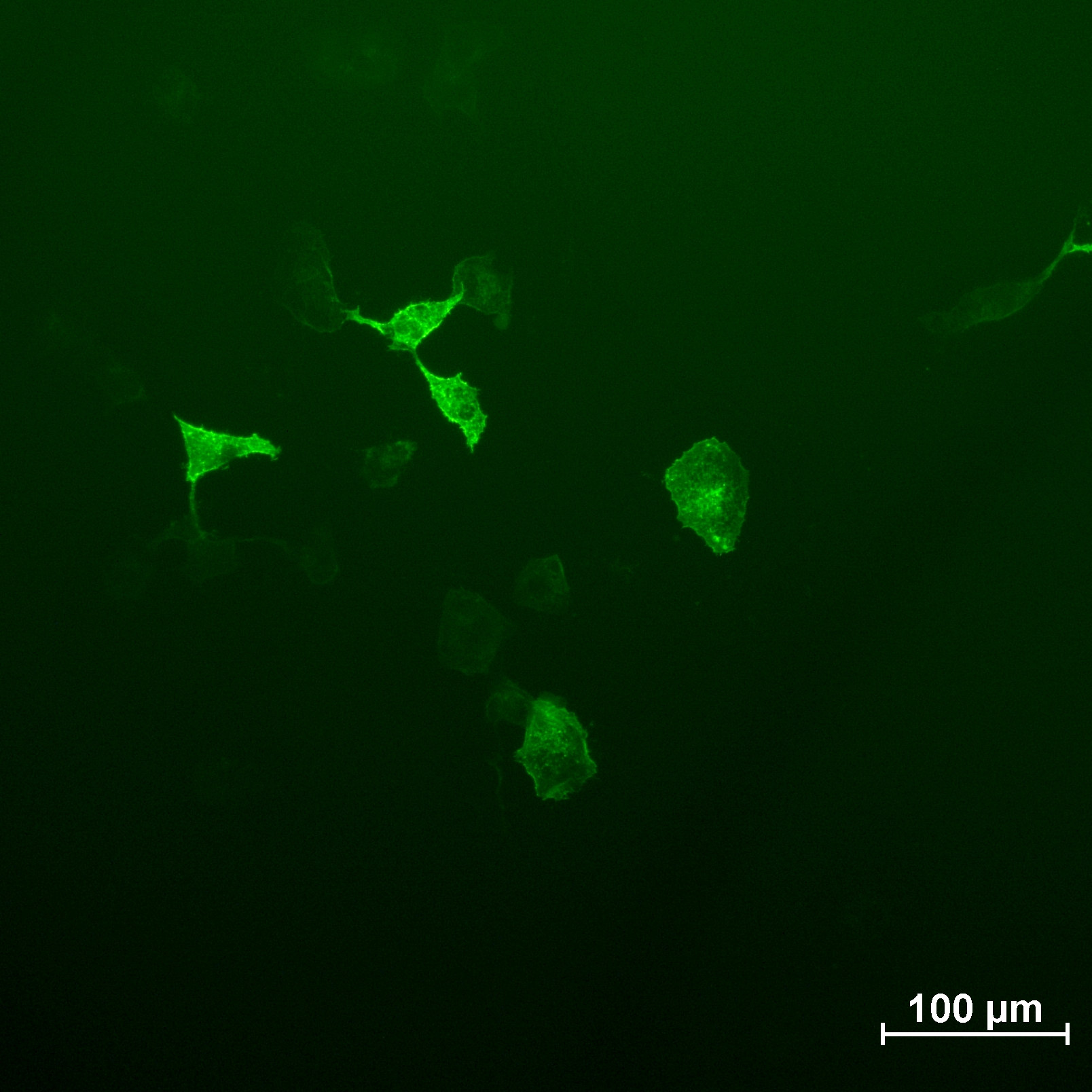

Supplement: Supplementary file 10 [file DataSheet8.zip › Cytoskeletal Damage/0/6-1.jpg]

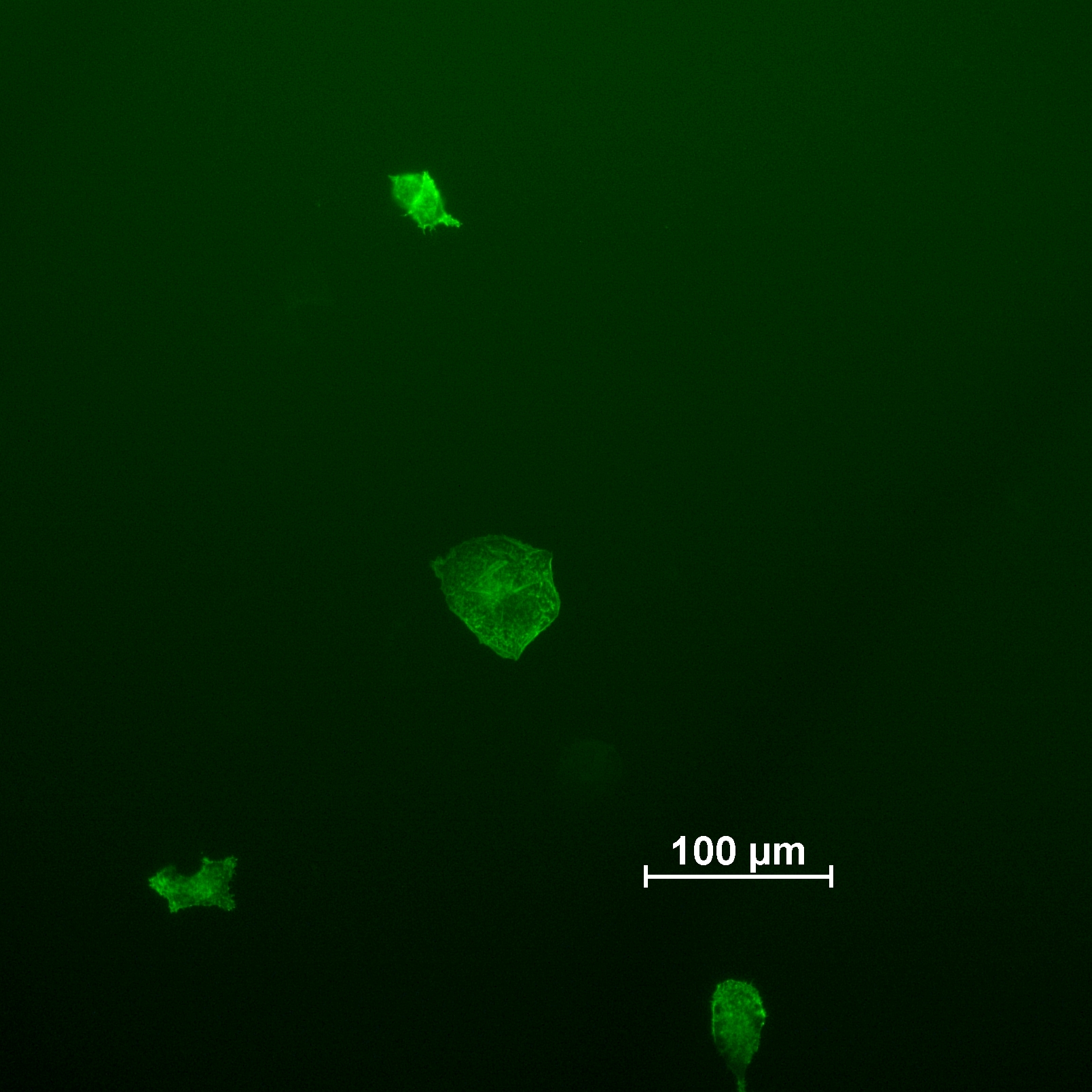

Supplement: Supplementary file 10 [file DataSheet8.zip › Cytoskeletal Damage/0/6.jpg]

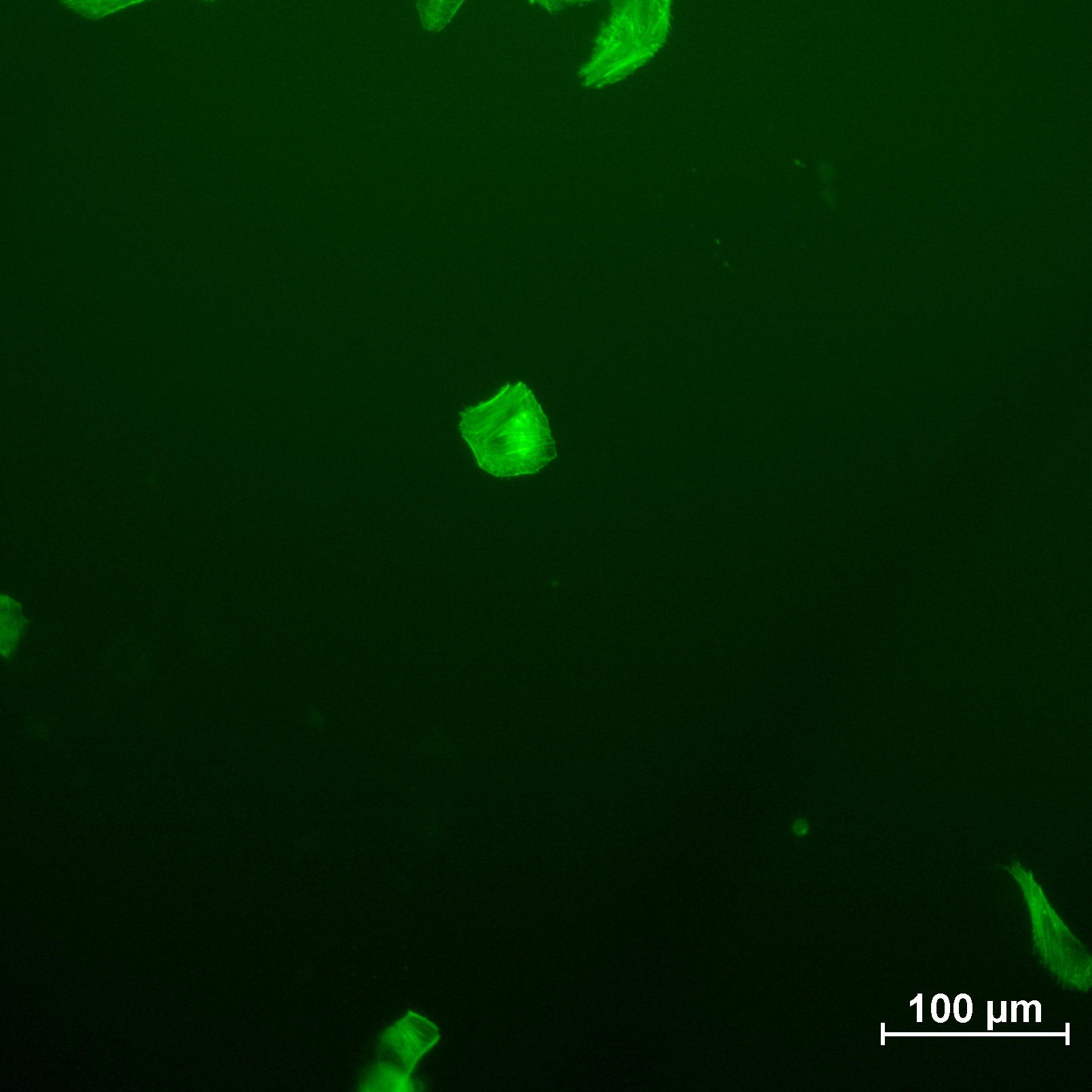

Supplement: Supplementary file 10 [file DataSheet8.zip › Cytoskeletal Damage/0.25/0.25.jpg]

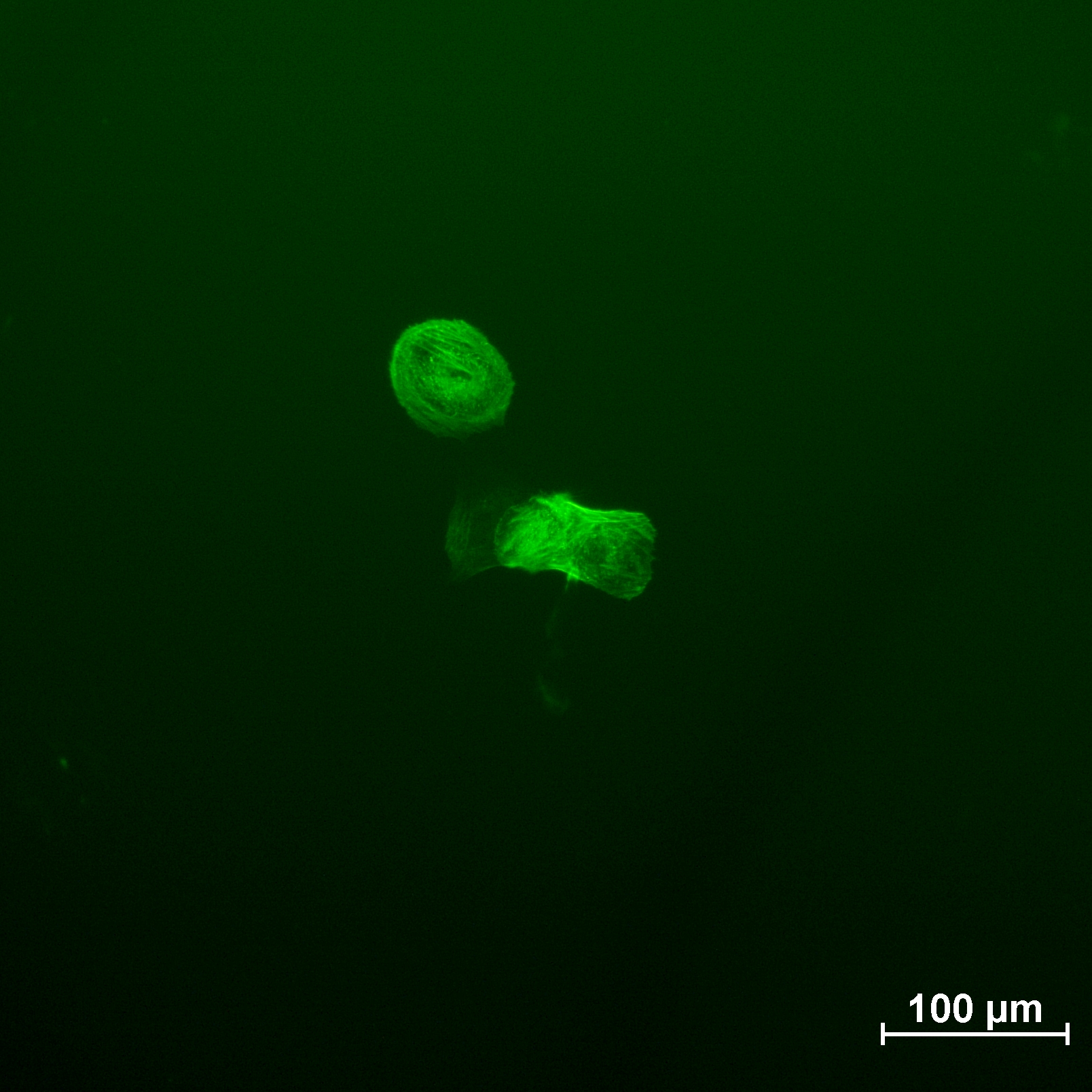

Supplement: Supplementary file 10 [file DataSheet8.zip › Cytoskeletal Damage/0.25/2.jpg]

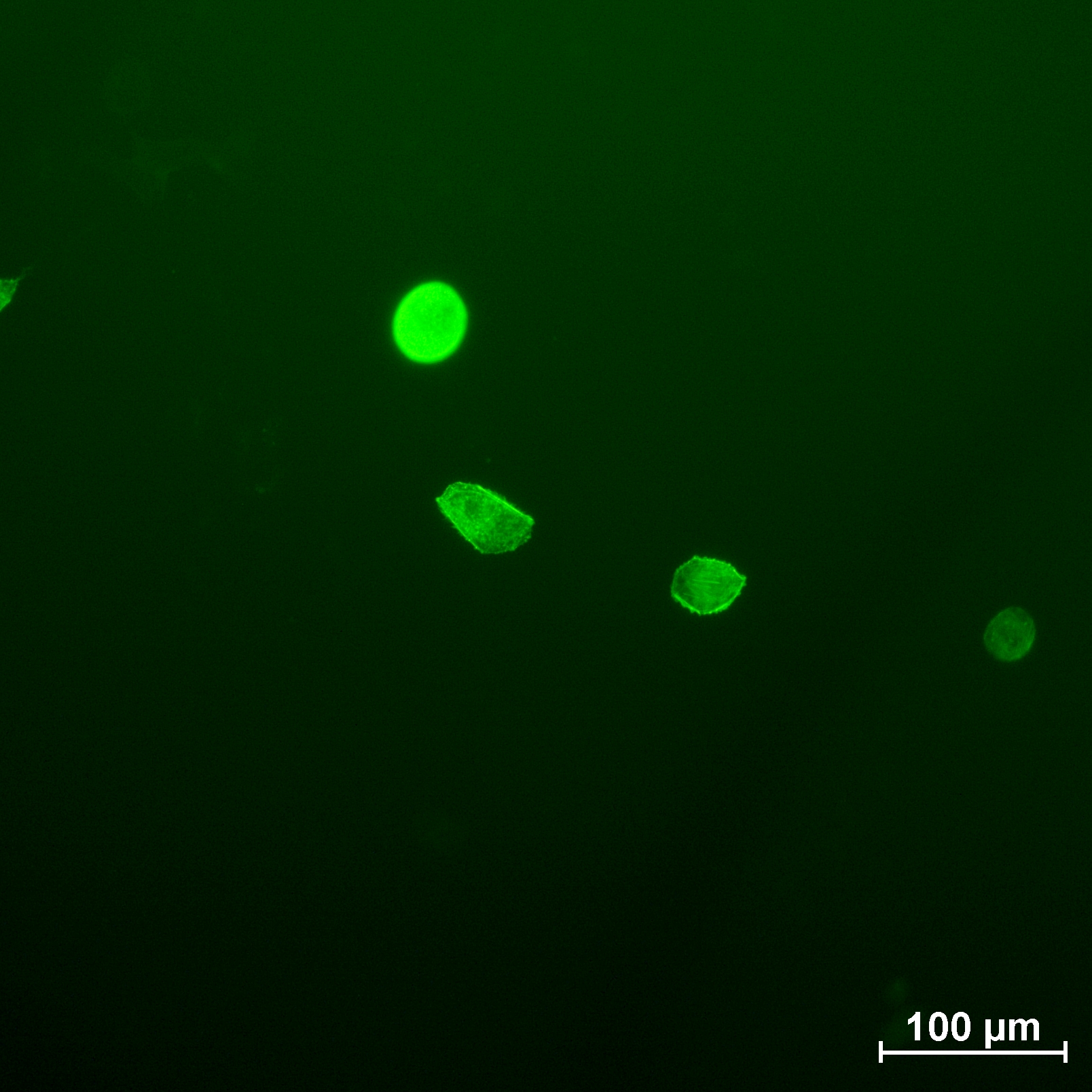

Supplement: Supplementary file 10 [file DataSheet8.zip › Cytoskeletal Damage/0.25/7.jpg]

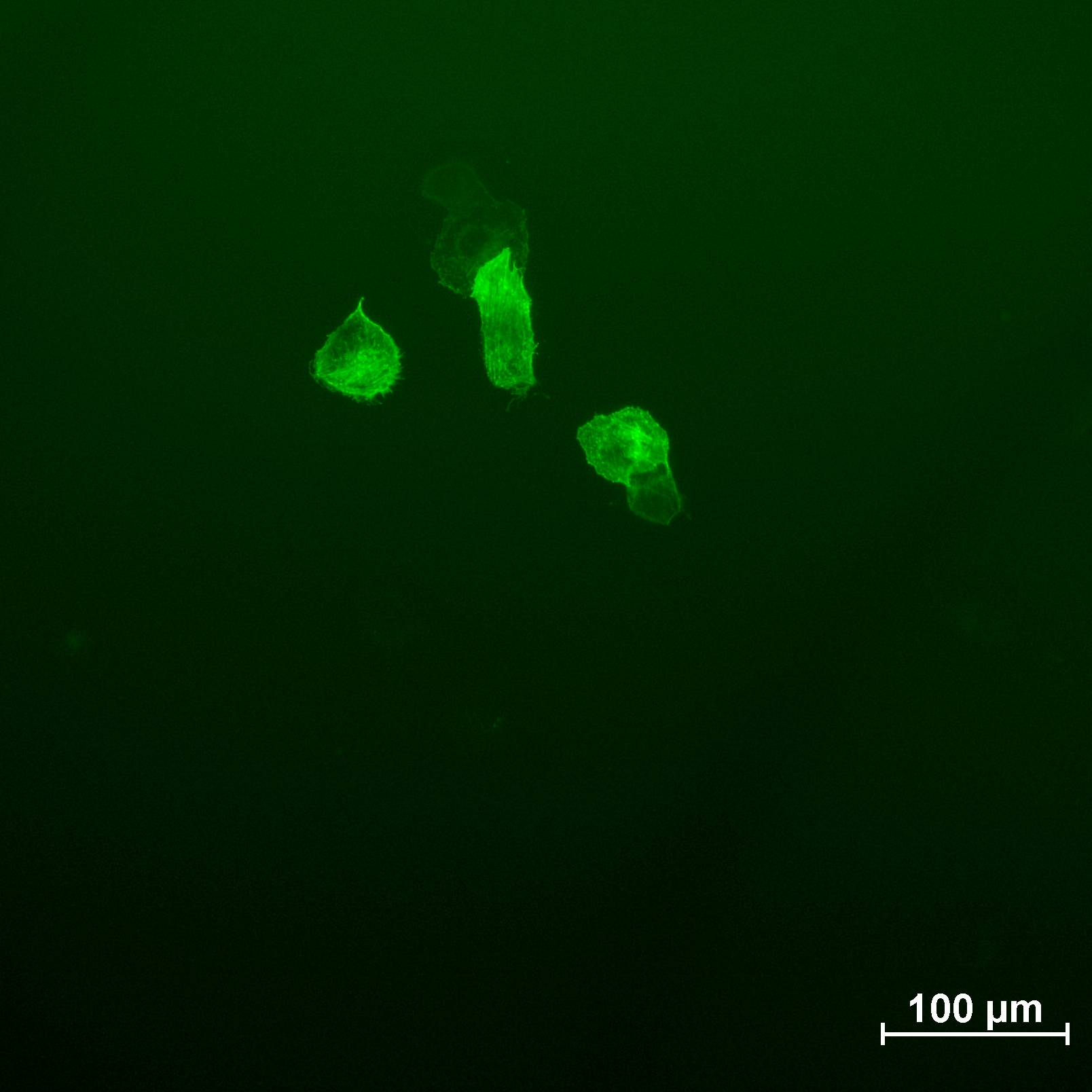

Supplement: Supplementary file 10 [file DataSheet8.zip › Cytoskeletal Damage/0.25/8.jpg]

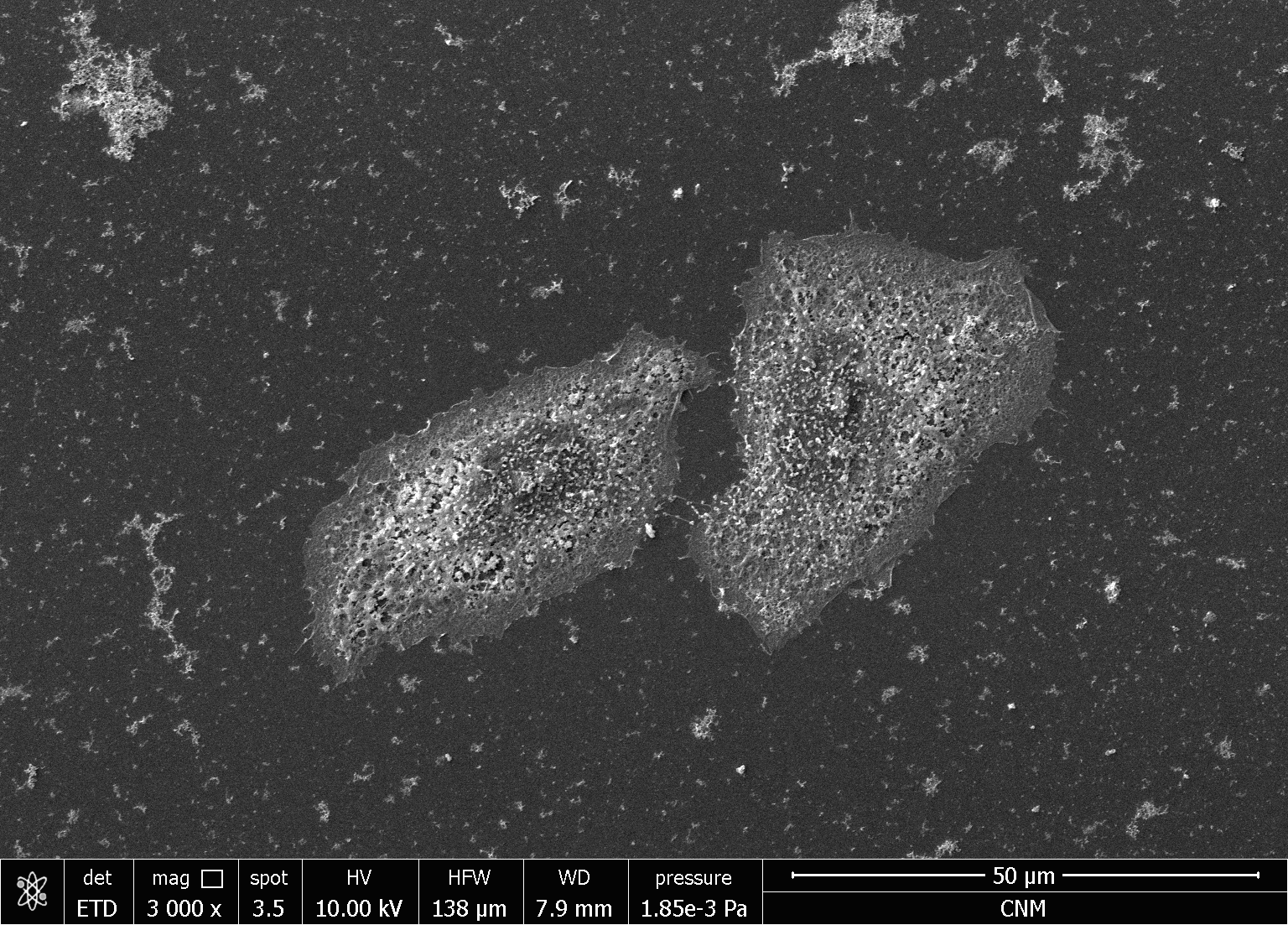

Supplement: Supplementary file 12 [file DataSheet10.zip › SEM20250613/0/1_006.tif]

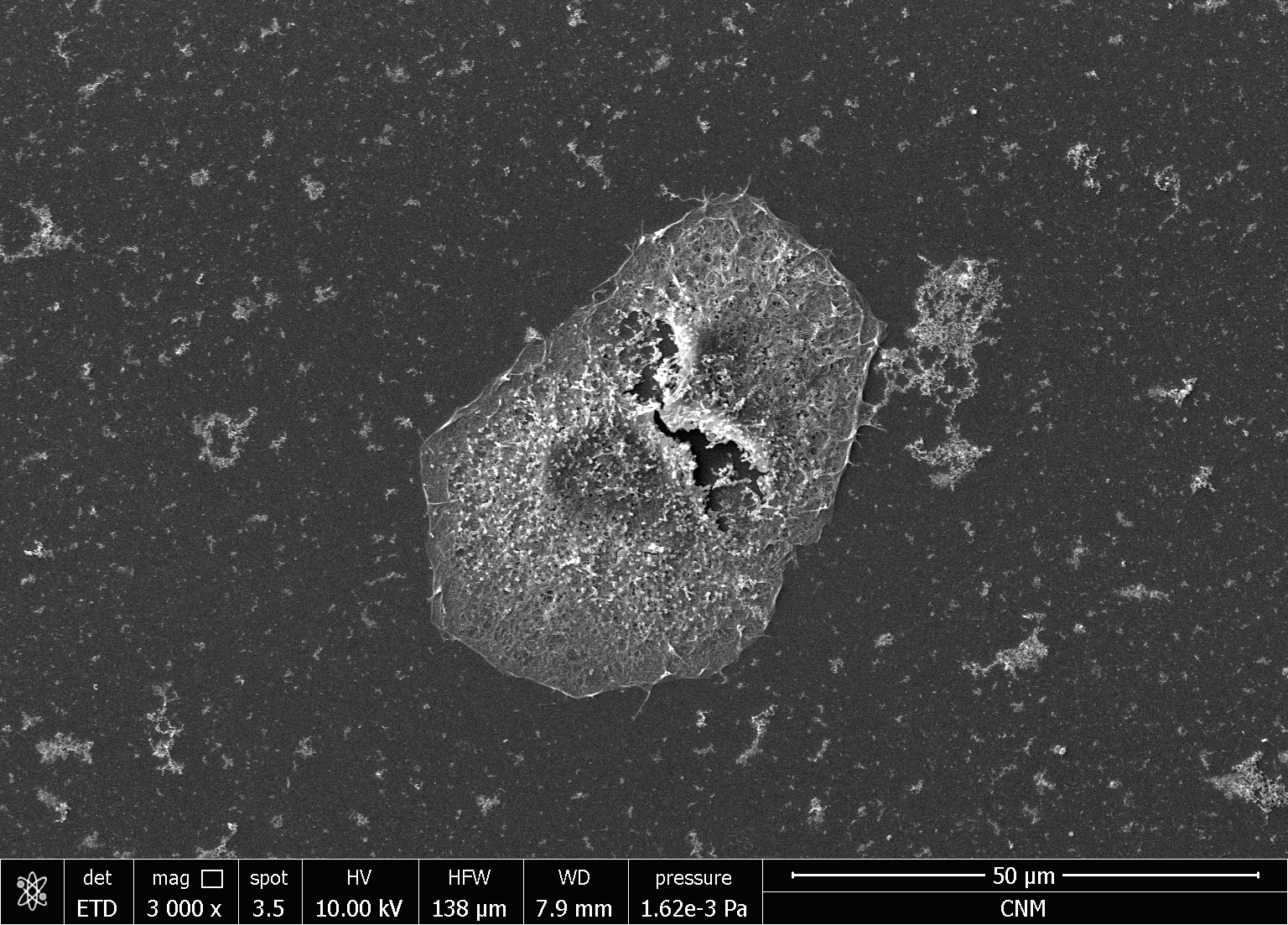

Supplement: Supplementary file 12 [file DataSheet10.zip › SEM20250613/0/1_008.tif]

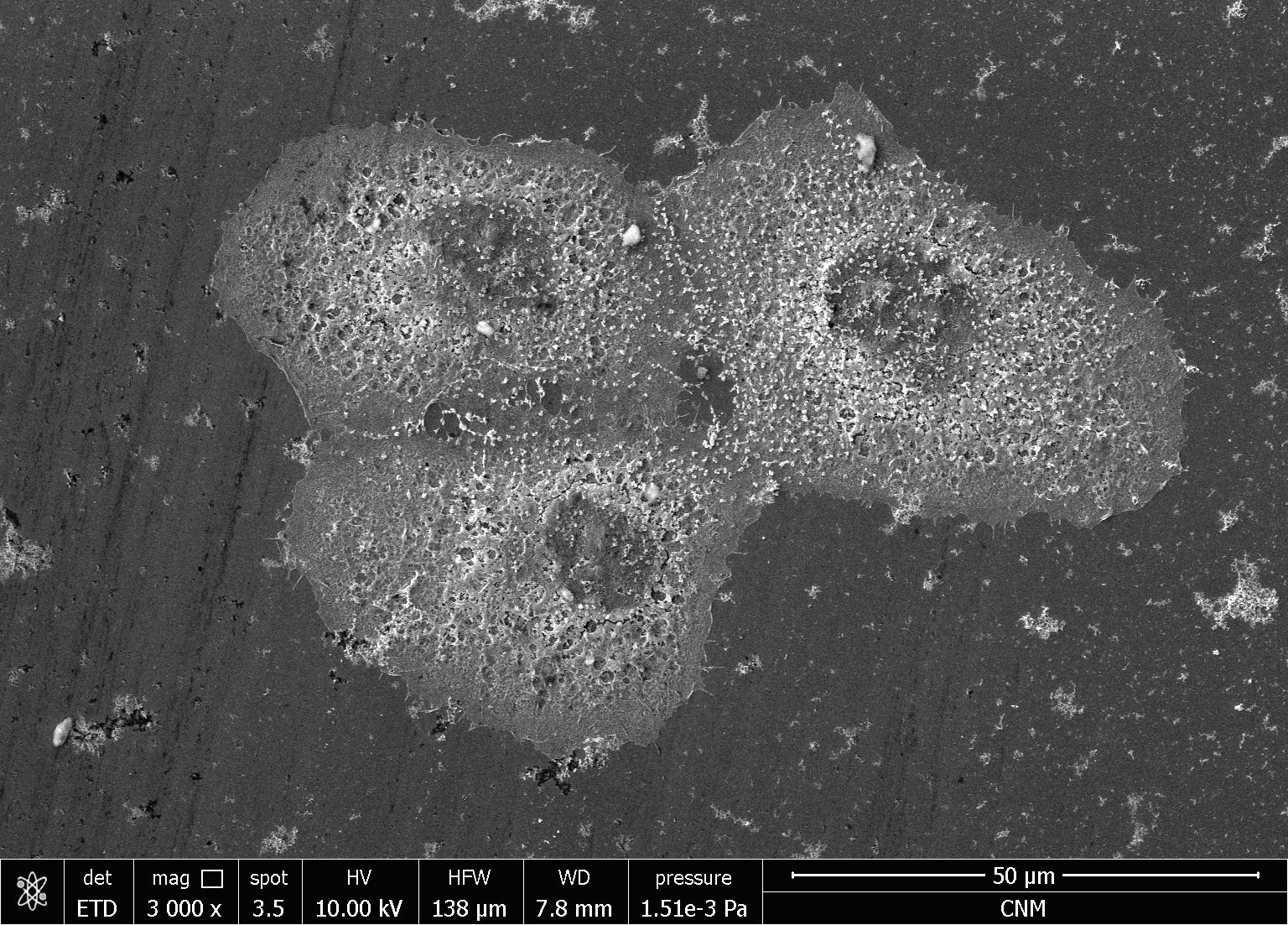

Supplement: Supplementary file 12 [file DataSheet10.zip › SEM20250613/0/1_009.tif]

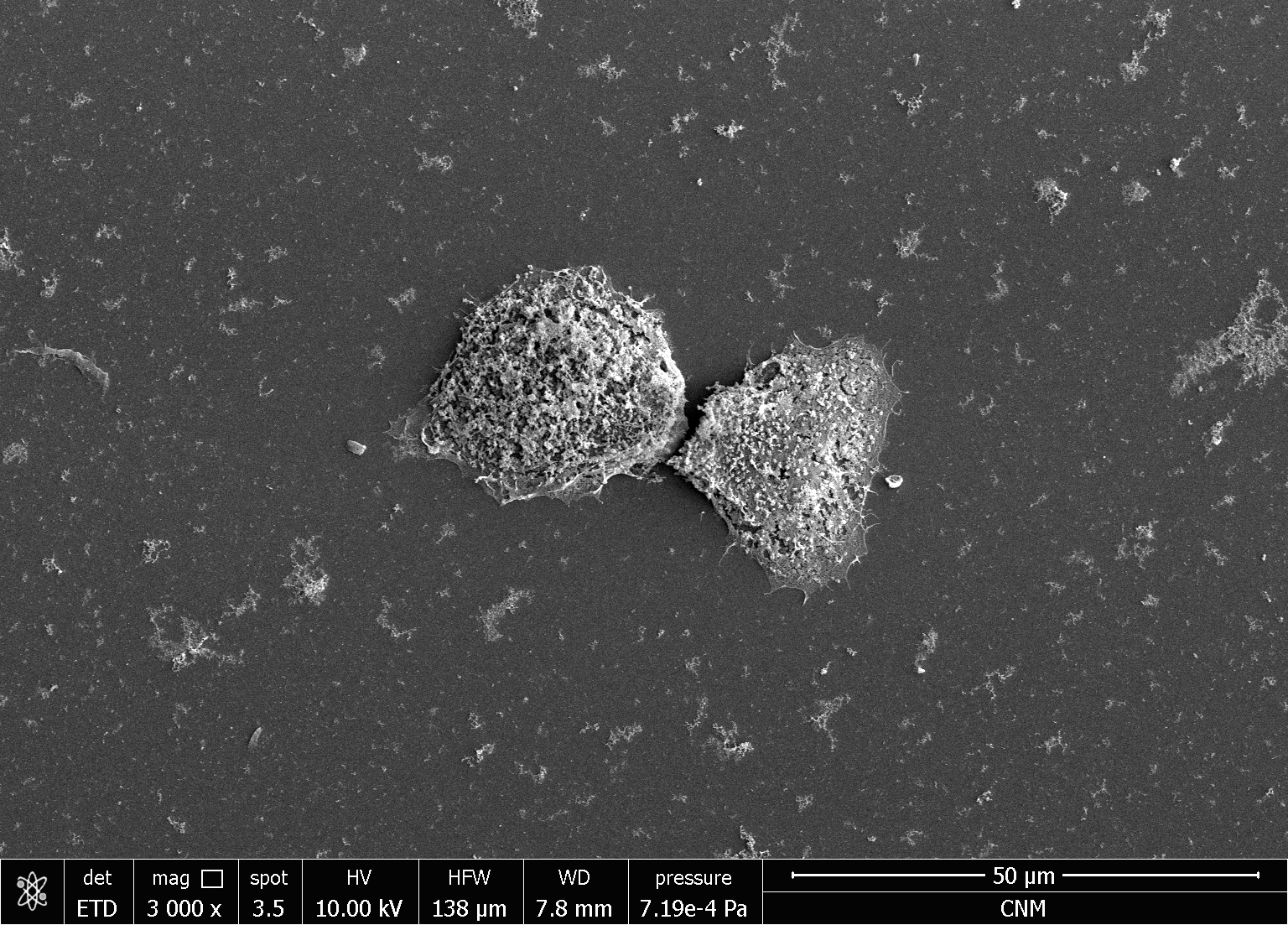

Supplement: Supplementary file 12 [file DataSheet10.zip › SEM20250613/0.15/1_001.tif]

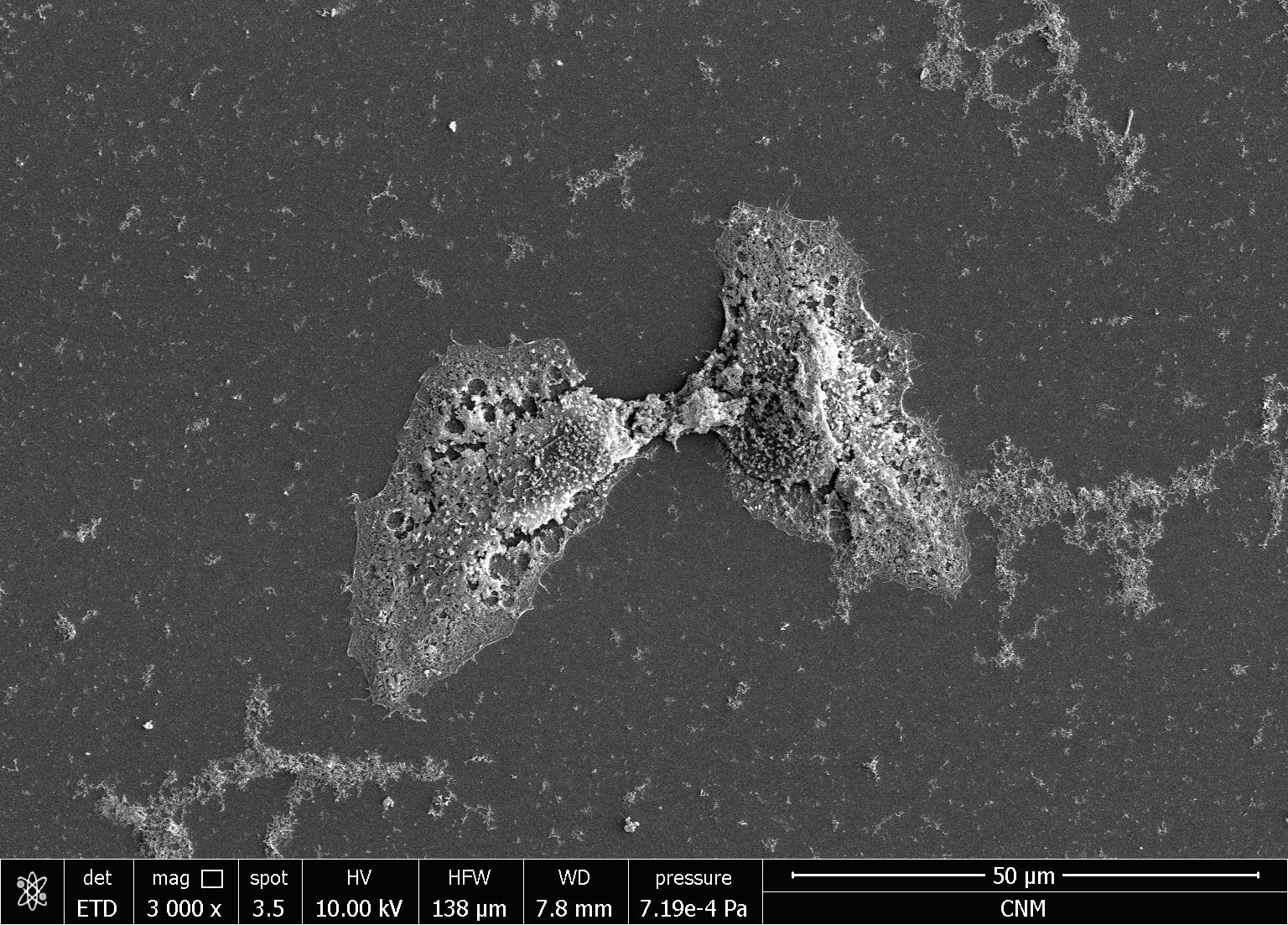

Supplement: Supplementary file 12 [file DataSheet10.zip › SEM20250613/0.15/1_002.tif]

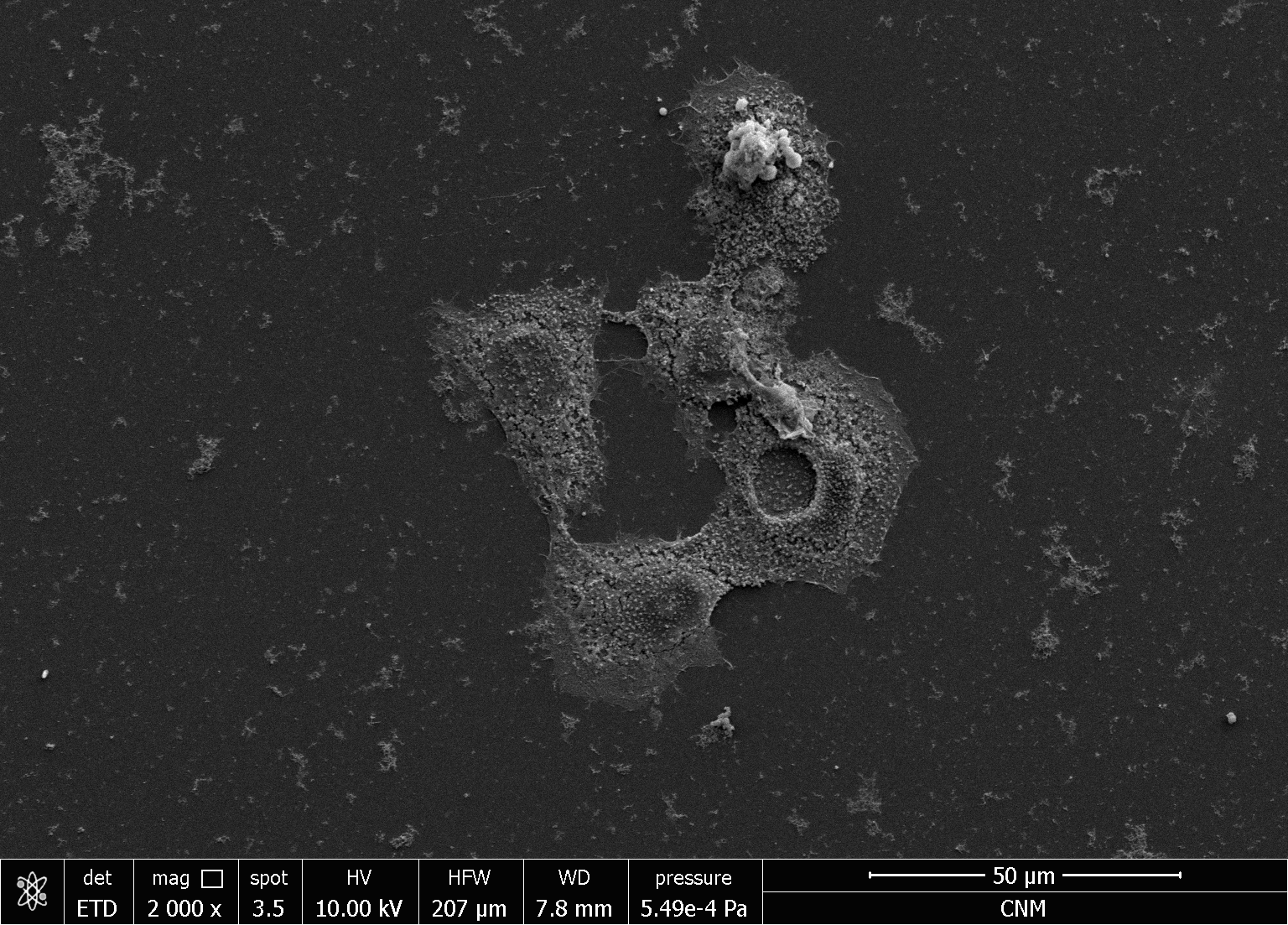

Supplement: Supplementary file 12 [file DataSheet10.zip › SEM20250613/0.15/1_007.tif]

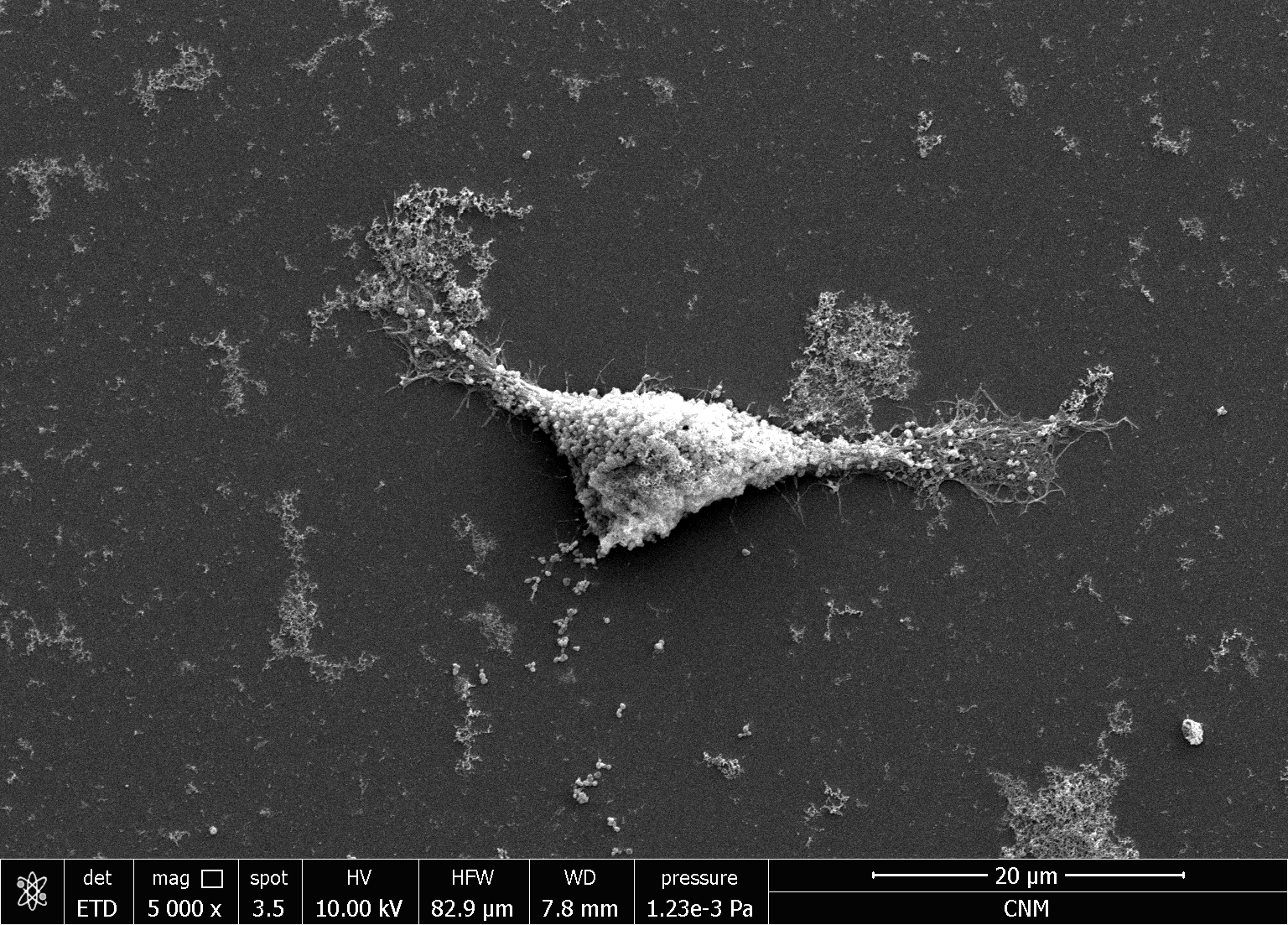

Supplement: Supplementary file 12 [file DataSheet10.zip › SEM20250613/0.2/1_00.tif]

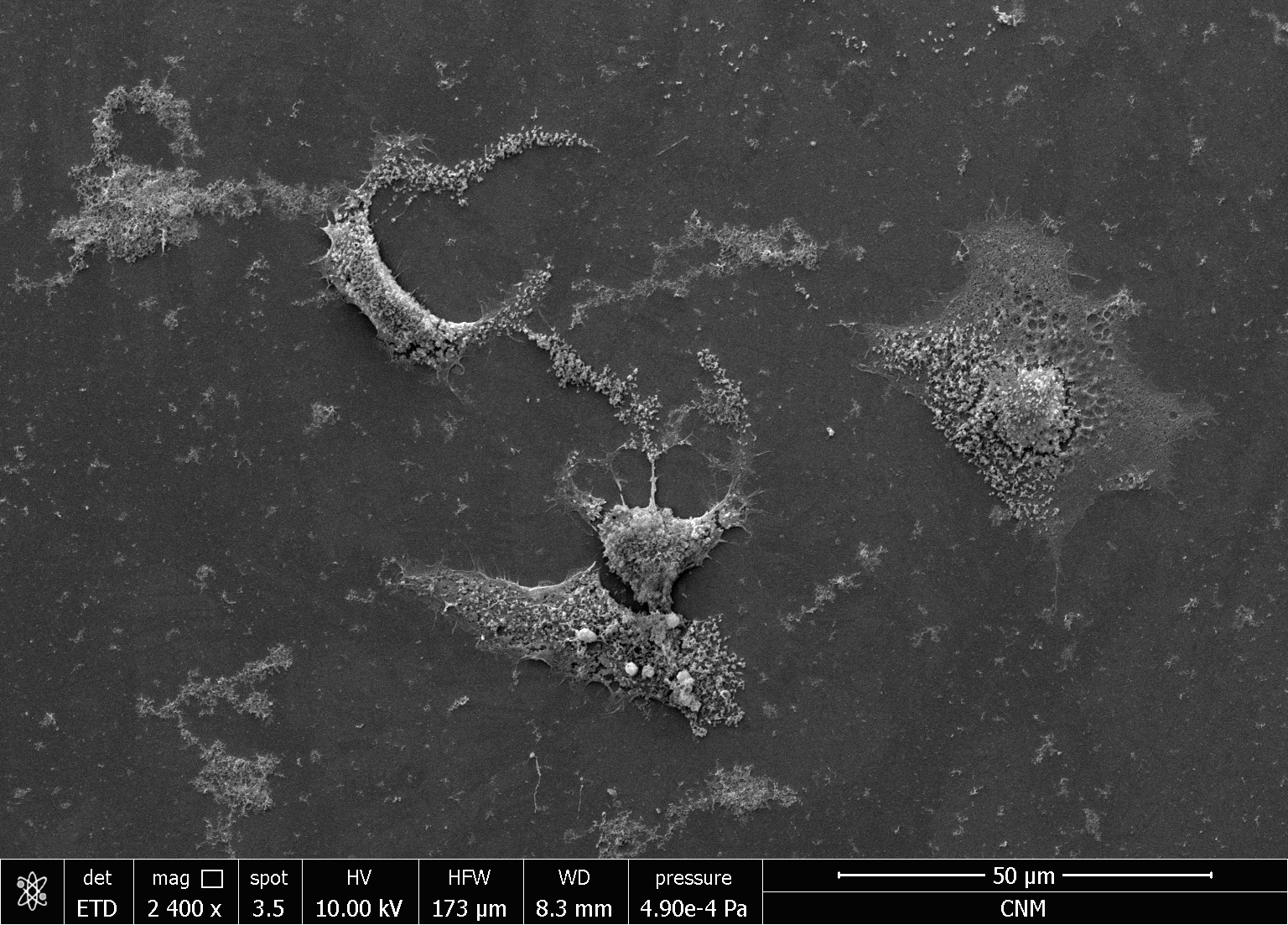

Supplement: Supplementary file 12 [file DataSheet10.zip › SEM20250613/0.2/1_008.tif]

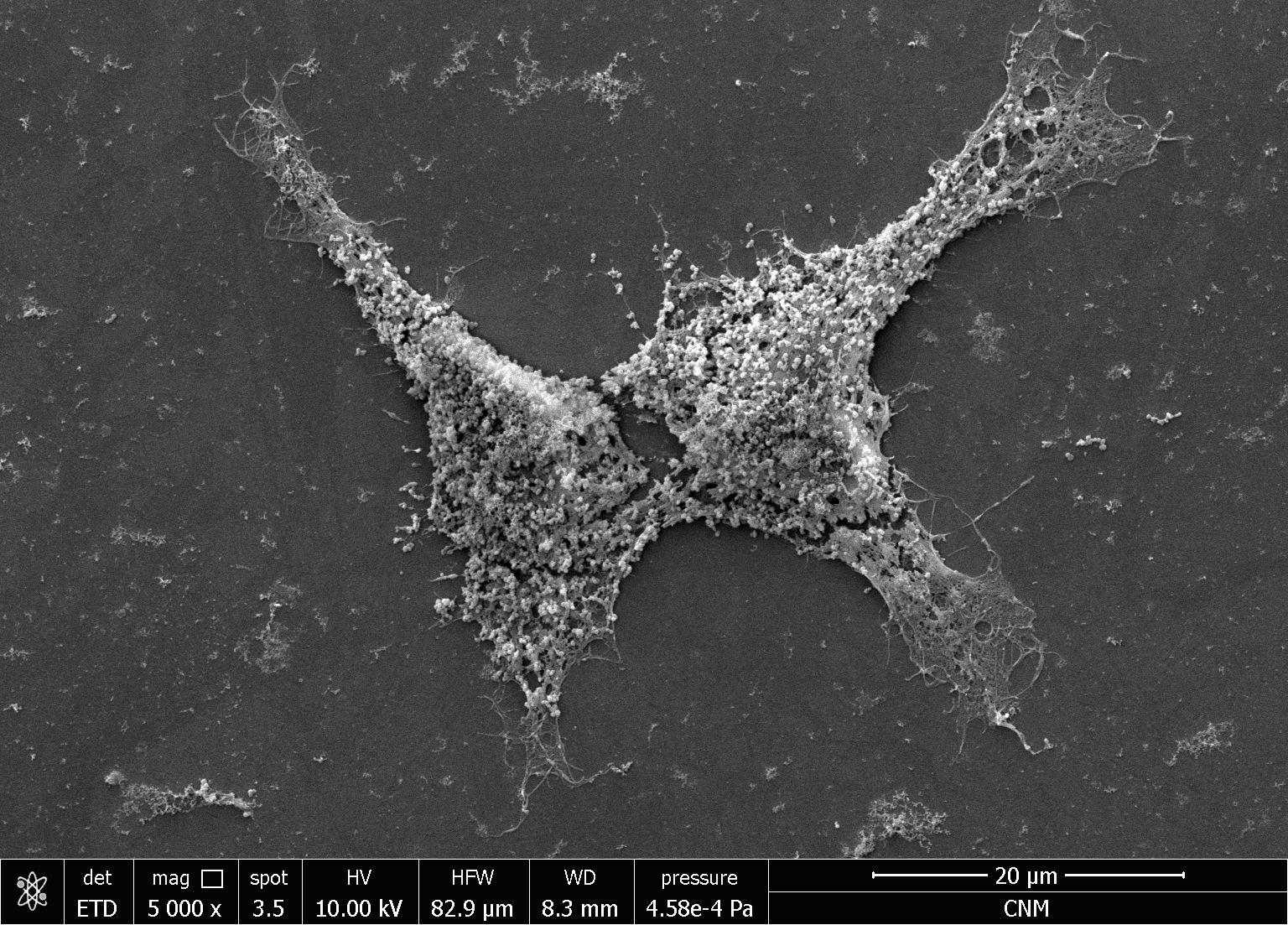

Supplement: Supplementary file 12 [file DataSheet10.zip › SEM20250613/0.2/1_009.tif]

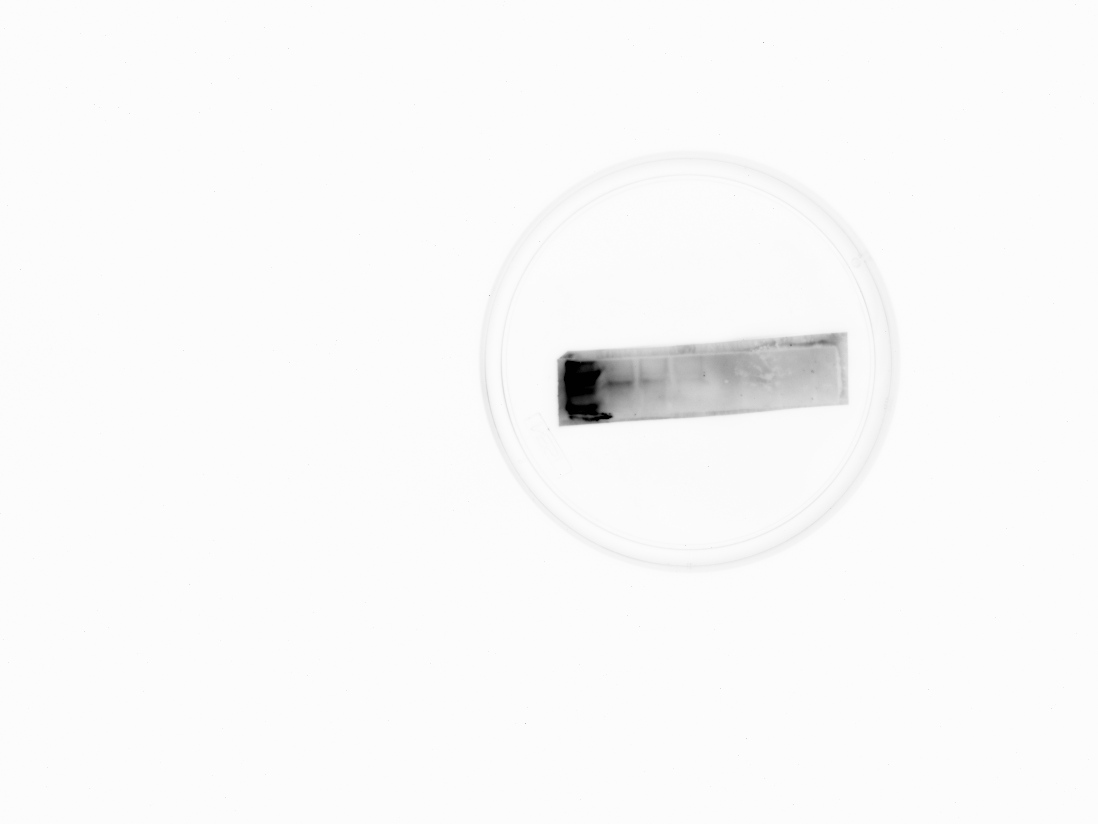

Supplement: Supplementary file 13 [file DataSheet11.zip › Werternblot/1.jpg]

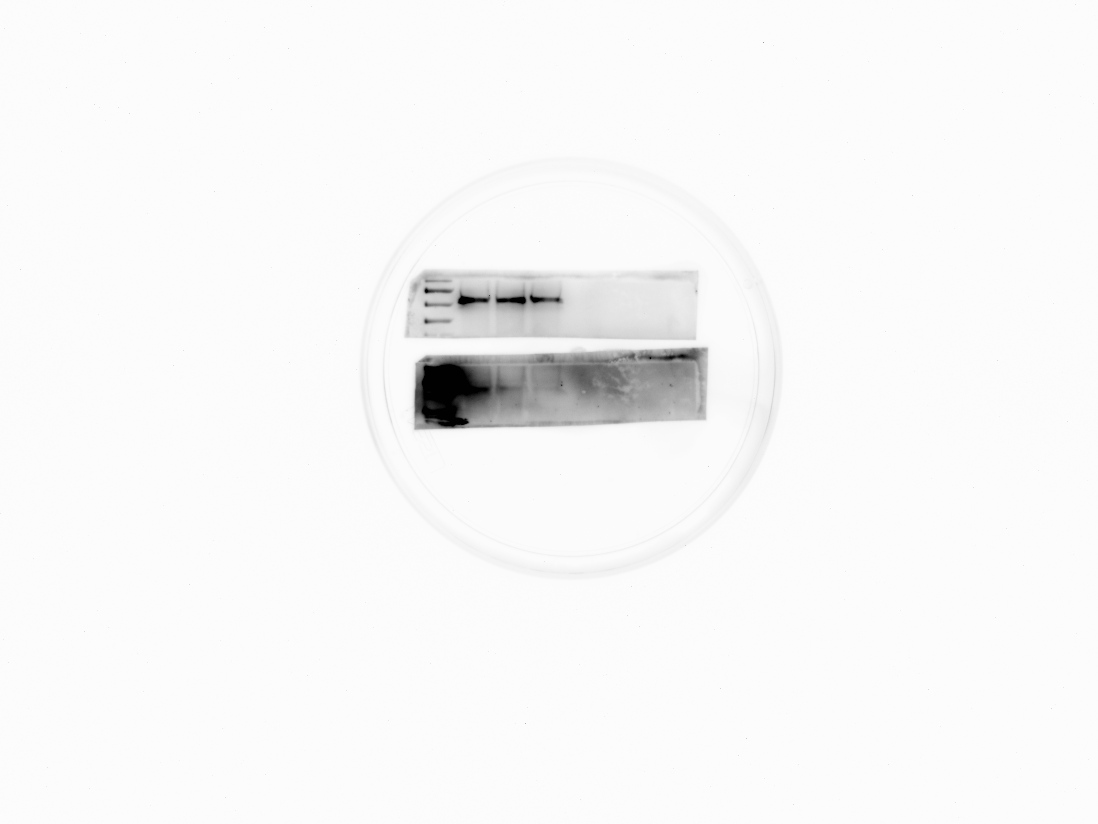

Supplement: Supplementary file 13 [file DataSheet11.zip › Werternblot/2.jpg]

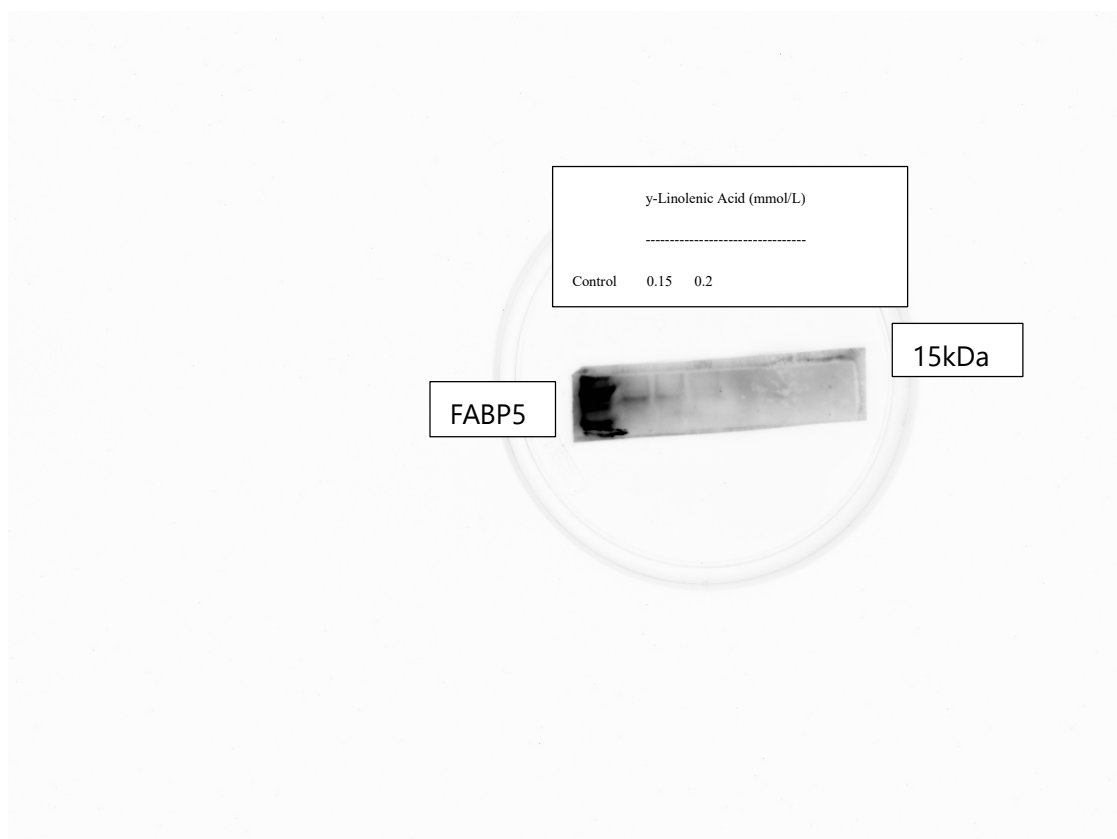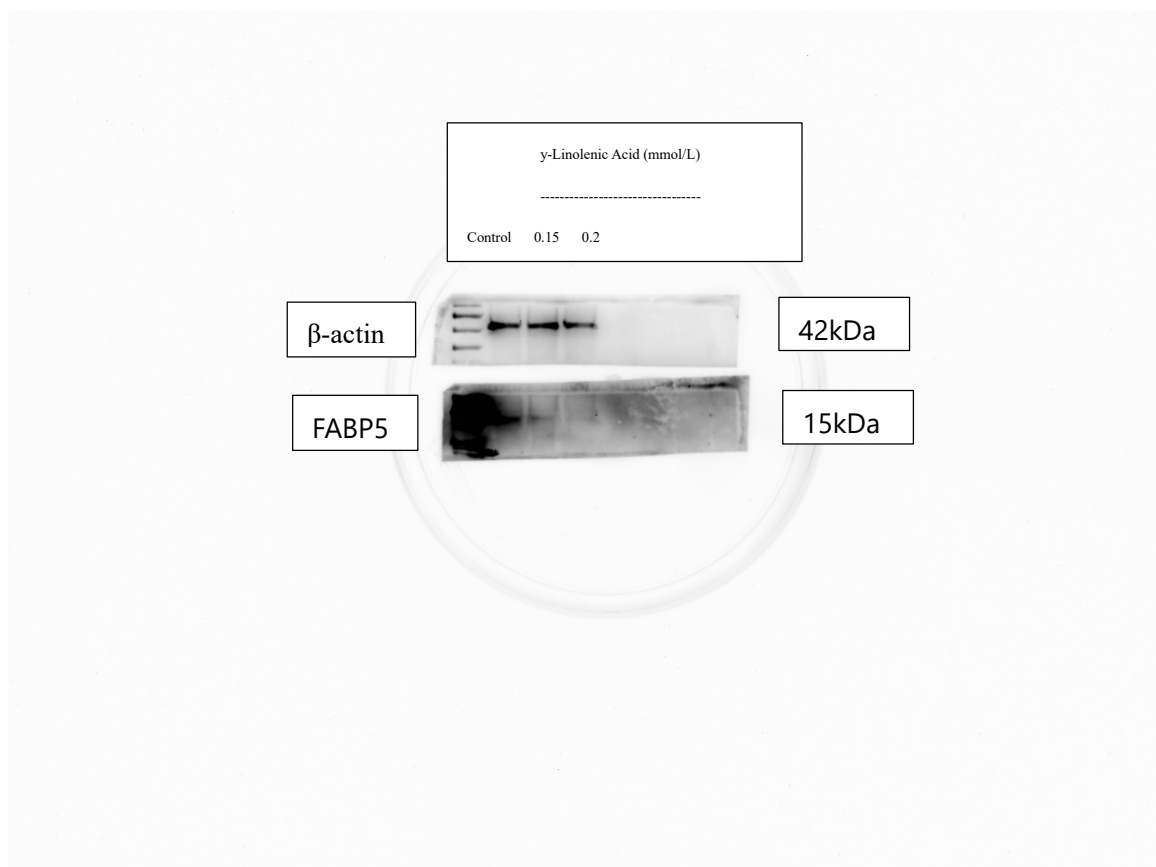

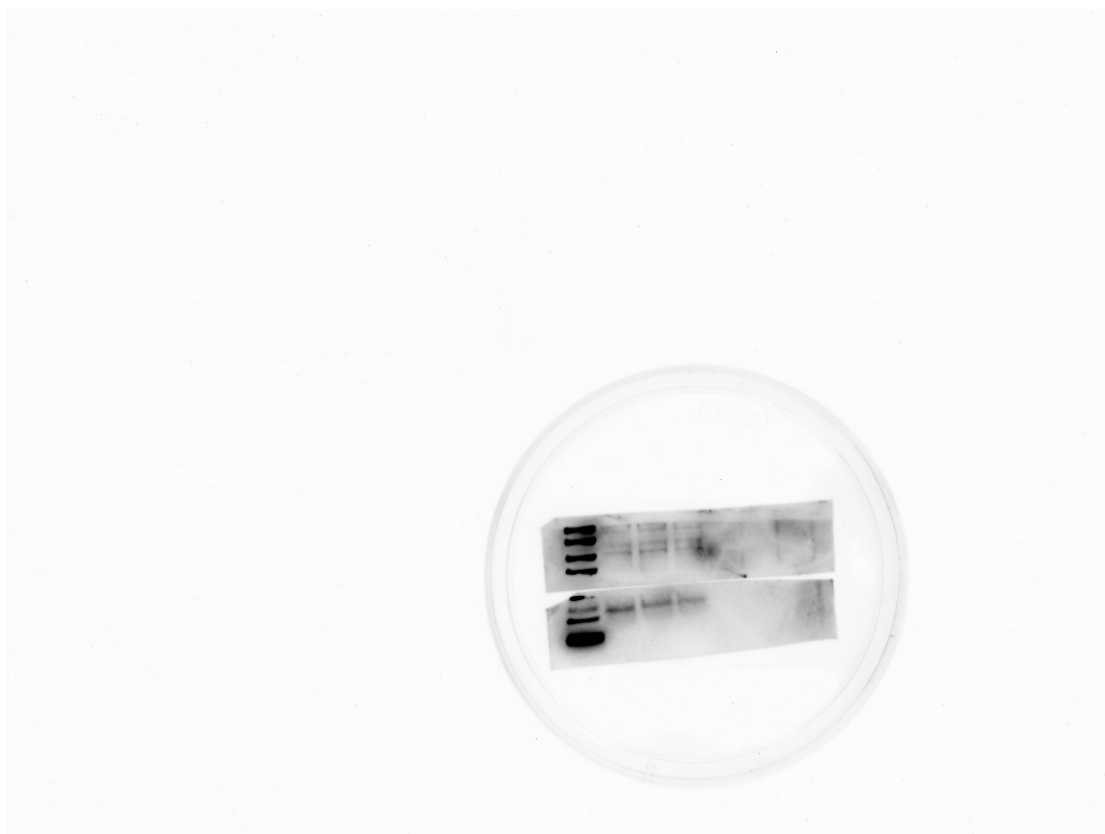

Supplement: Supplementary file 13 [file DataSheet11.zip › Werternblot/WB.pdf]
