# Supplementary material for: Identification and validation of γ-Linolenic acid as a natural FABP5 inhibitor in hepatocellular carcinoma through deep learning and experimental approaches
Source: Front Immunol. 2026 Jan 28;17:1700347. doi: 10.3389/fimmu.2026.1700347 (PMC12891133; doi:10.3389/fimmu.2026.1700347)
Supplement: Supplementary file 11 [file DataSheet9.zip › Flow Cytometry/25-Jul-2025-Layout-Batch(1).pdf]

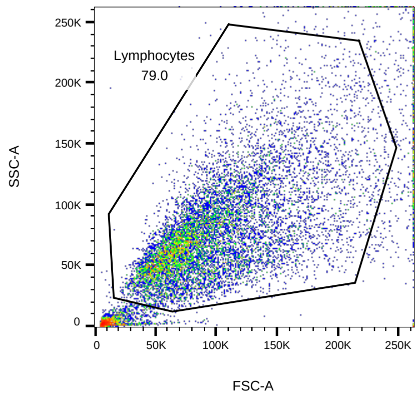

20250718_Tube_004_004.fcs
Ungated
12721

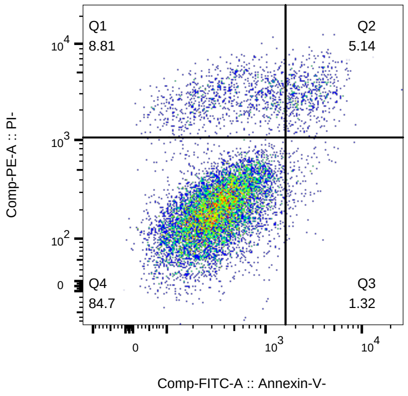

20250718_Tube_004_004.fcs
Lymphocytes
10052

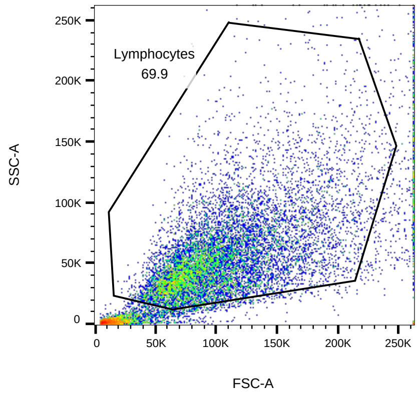

20250718_Tube_005_005.fcs
Ungated
14204

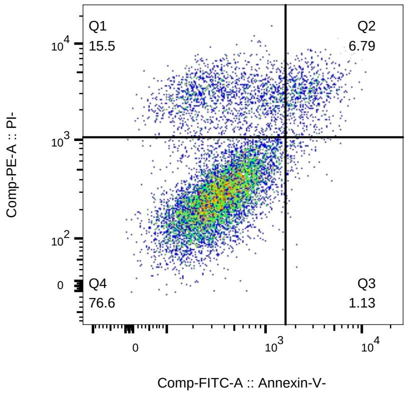

20250718_Tube_005_005.fcs
Lymphocytes
9924

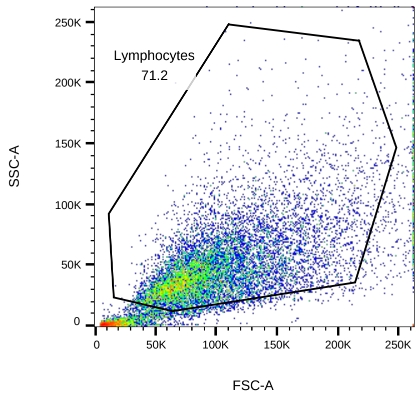

20250718_Tube_006_006.fcs
Ungated
13818

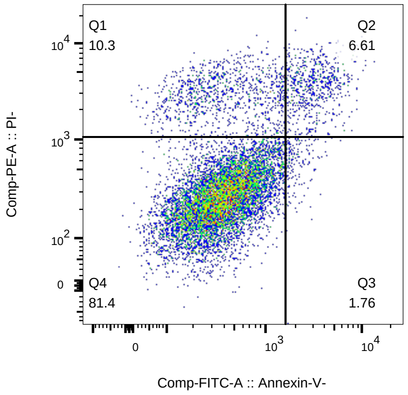

20250718_Tube_006_006.fcs
Lymphocytes
9836

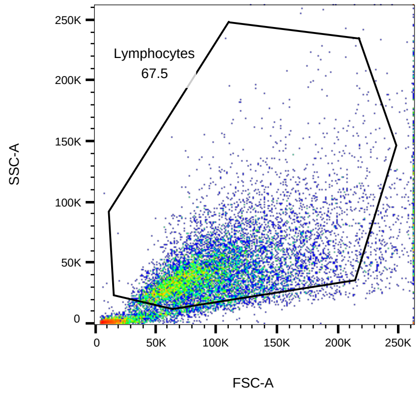

20250718_Tube_007_007.fcs
Ungated
13460

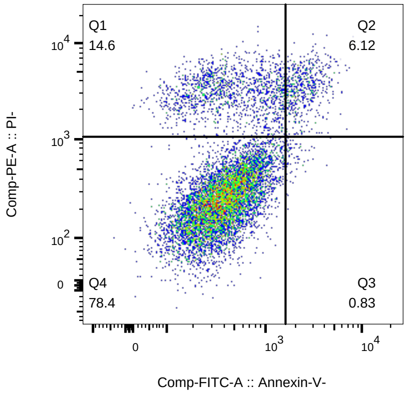

20250718_Tube_007_007.fcs
Lymphocytes
9083

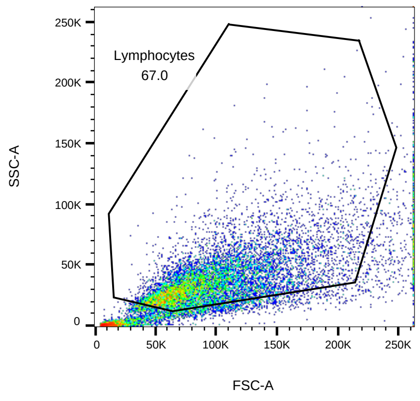

20250718_Tube_008_008.fcs
Ungated
13142

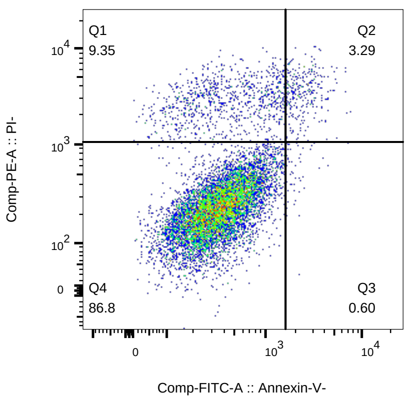

20250718_Tube_008_008.fcs
Lymphocytes
8802
